# Bioinspired “liquid-solid” biphasic dressing with exudate-gating transport, defense-attack antibacterial activity, and anti-adhesion property for exuding infected wound therapy

**DOI:** 10.1016/j.bioactmat.2026.04.001

**Published:** 2026-04-06

**Authors:** Zizhao Chen, Changming Wang, Yixuan Li, Chunsheng Guan, Mingzhang Li, Hao Shen, Malcolm Xing, Botao Song, Jiang Chang

**Affiliations:** a Key Laboratory of Synthetic and Natural Functional Molecule of the Ministry of Education, College of Chemistry and Materials Science, Northwest University, Xi'an, 710069, China; b Department of Orthopedics, Shanghai Sixth People's Hospital Affiliated to Shanghai Jiao Tong University School of Medicine, Shanghai Jiao Tong University, Shanghai, 200233, China; c Department of Mechanical Engineering, University of Manitoba, Winnipeg, MB, R3T 2N2, Canada; d Joint Centre of Translational Medicine, The First Affiliated Hospital of Wenzhou Medical University, Wenzhou, 325000, China; e Zhejiang Engineering Research Center for Tissue Repair Materials, Wenzhou Institute, University of Chinese Academy of Sciences, Wenzhou, 325000, China

**Keywords:** Exuding infected wounds, Liquid-solid biphase, Self-adaptive gating transport, Defense-attack antibacterial, Anti-adhesion

## Abstract

The management of heavily exuding infected wounds is complicated by excessive exudates, persistent bacterial colonization, and secondary trauma during dressing changes. Here, we develop a bioinspired “liquid-solid” biphasic dressing designed to address these challenges simultaneously. This dressing integrates a hylarana guentheri mucus-inspired medical antibacterial oil (guest phase) within a concave nanofiber membrane that mimics the structure of octopus suckers (host phase). This unique architecture enables an exudate-triggered self-adaptive gating mechanism, where the oil-filled concaves act as pressure-deformable gates. High hydrostatic pressure from excess exudate opens the gates for unidirectional transport, while low pressure maintains a closed state, thus dynamically regulating moisture and creating an optimal healing environment. The integrated oil, infused with thymol, establishes a synergistic “defend-and-attack” antibacterial barrier, preventing biofilm formation and neutralizing planktonic *E. coli* and *S. aureus*. The same oil layer provides a low-adhesion interface, enabling nearly painless dressing changes with minimal peeling force. In an infected wound model, the dressing accelerates healing, suppresses inflammatory cytokine expression, and reduces bacterial burden. This “liquid-solid” strategy offers a promising paradigm for developing next-generation smart biomaterials for complex wound care.

## Highlights

• The liquid-solid biphasic dressing is designed by drawing inspiration from nature.
• The biphasic dressing enables exudate-triggered gating transport behavior.
• The biphasic dressing facilitates defense-attack dual-modal antibacterial property.
• The biphasic dressing demonstrates excellent anti-adhesive feature.
• The biphasic dressing accelerates the healing of infected exuding wound.

## Graphical abstract

**Figure:**
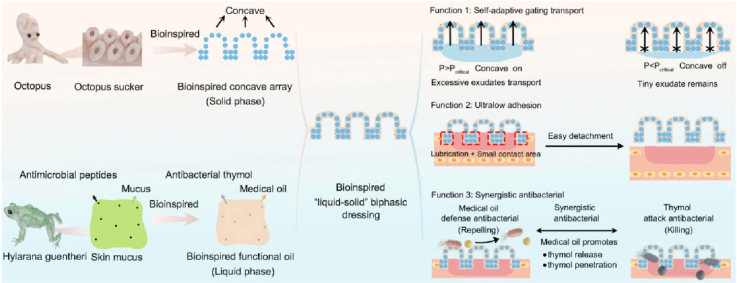

## 1 Introduction

Exuding infected wounds are often associated with intractable complications, including excessive exudates production and significant bacterial colonization, both of which markedly hinder the wound healing process [[1], [2], [3]]. The excessive accumulation of wound exudates can lead to tissue edema, periwound skin maceration, and an increase in wound size. In addition to these moisture-related complications, the bacteria within the wound site may result in infection and intensify local inflammatory responses. Certain bacterial strains are particularly capable of forming biofilms on the wound surface, leading to persistent and challenging infections that significantly impair healing [4]. The prolonged healing period necessitates frequent dressing changes, which may cause mechanical trauma to newly formed tissue and further delay recovery [5]. To date, a variety of advanced wound dressings, including drug-loaded hydrogels, nanozyme-based dressings, and hydrogen sulfide (H_2_S)-releasing systems, have been developed to address exuding infected wounds [[6], [7], [8]]. Nevertheless, most currently reported dressings exhibit functional limitations. Therefore, there is an urgent need for advanced multifunctional dressings that integrate effective moisture regulation, robust antibacterial activity, and anti-adhesive property.

Janus dressings composed of a thin hydrophobic nanofiber layer combined with a hydrophilic layer have been reported as promising solutions for managing excessive wound moisture [9]. The asymmetric wettability of these dressings facilitates exudate transport from the hydrophobic layer to the hydrophilic layer, while effectively preventing backflow to the wound bed [10]. Building upon this concept, we have found that nanofiber membrane with concave array structure to enable rapid unidirectional drainage of excessive exudates [11]. However, complete removal of exudates from the wound bed may potentially lead to excessive desiccation [12], and a completely dry wound microenvironment has been demonstrated to negatively impact the wound healing process, as such conditions are not conducive to cell migration and proliferation [13,14]. Therefore, dynamic regulation of wound moisture through the removal of excessive exudates while maintaining an optimal moisture level may represent a more effective strategy for promoting wound healing [15].

In addition to the problems of wound moisture management, exuding wounds are frequently accompanied by bacterial infection [16,17], as the excessive exudates, which are rich in proteins and glucose, provide an ideal microenvironment for bacterial proliferation [18]. Currently, the primary antibacterial strategies include the antibacterial agent-releasing approach [19], contact-killing mechanisms [20], and anti-fouling coatings [21]. Dressings with antibacterial agent-releasing properties may have high efficacy against planktonic bacteria [22]. Unfortunately, these released agents may exhibit limited penetration through bacterial biofilms, thereby failing to eradicate the embedded bacterial communities [23,24]. Contact-killing dressings rely on specific micro/nanostructures to exert antibacterial effects [25]. However, the mechanical stability of the microstructures during application is one of the problems for long term effectiveness of the approach. In recent years, liquid-infused porous surfaces have been developed for anti-biofouling application [26]. The underlying principle is that the lubricating oil layer can effectively inhibit bacterial adhesion, thereby reducing biofilm formation. However, due to the insufficient stability of the oil layer, the long-term antibacterial durability of these dressings is compromised. Moreover, such dressings cannot address wounds where bacteria have already adhered. By integrating additional antibacterial drug loading function into liquid-infused porous dressings, these materials may not only inhibit initial bacterial colonization through the lubricating effect but also eliminate adhered bacteria through controlled release of drugs, thereby achieving a synergistic antibacterial effect.

Prolonged wound healing often requires repeated dressing changes, which may cause damage to newly formed tissues. In recent years, on-demand removable dressings and superhydrophobic dressings have been specifically developed to minimize secondary trauma during wound management [27]. However, these advanced dressings typically involve complex application procedures and are only effective in preventing adhesion during the early stages of wound healing. Therefore, the development of novel strategies capable of maintaining long-term anti-adhesive property is of critical importance for the effective management of chronic wounds.

**Fig. 1:**
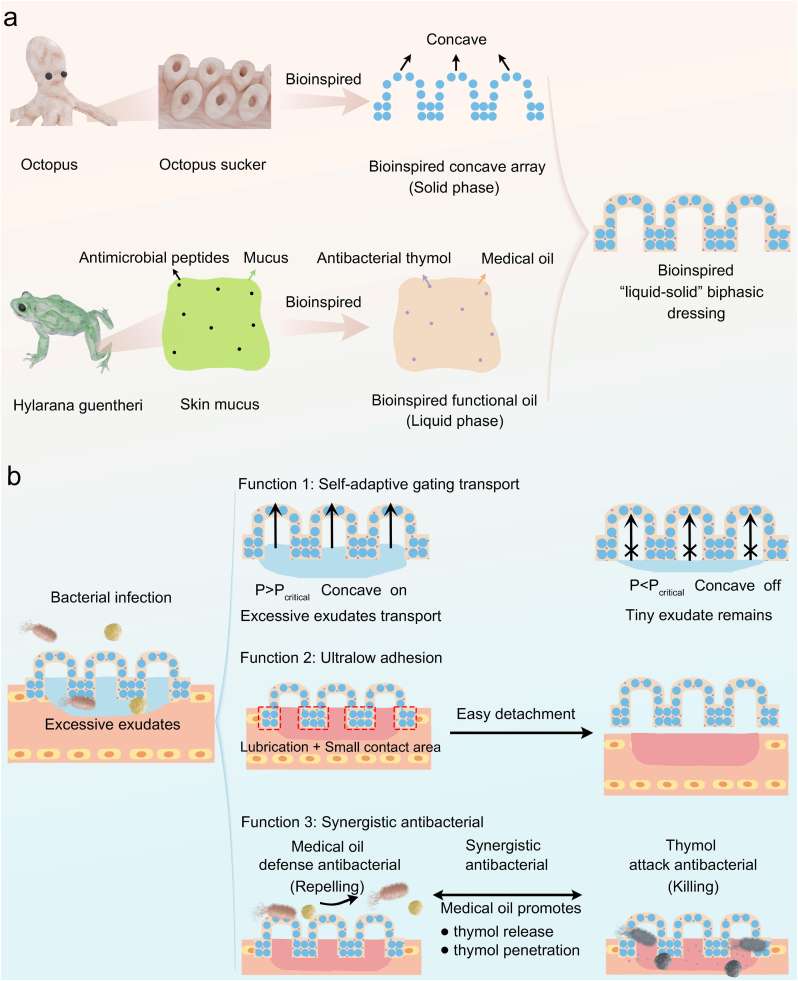
Designing concept of the bioinspired “liquid-solid” biphasic dressing for exuding infected wound repair. (**a**) The bioinspired design strategy inspired from octopus sucker and skin mucus. (**b**) The exceptional multifunction with self-adaptive liquid-gating transport, anti-adhesion ability, and dual-modal synergistic antibacterial feature for the treatment of exuding infected wound.

Natural organisms offer valuable inspiration for advanced wound dressing design through their unique structures, compositions, and functions. The octopus sucker (OS) possesses a distinctive concave array structure that confers exceptional suction performance, and we assume that, by emulating the structural and functional characteristics of OS, the dressing may enable effective liquid suction and enhanced exudate transport performance. In addition to OS, the hylarana guentheri frog (HG) may also present a promising biological model for dressing innovation. The liquid mucus secreted by its skin not only provides effective lubrication but also contains antimicrobial peptides that exhibit dual-modal antibacterial properties through both physical defense and chemical attack mechanisms [28]. These two distinct functions in the mucus closely align with key objectives in dressing design. Herein, we propose a novel “liquid-solid” biphasic dressing (OSHG) by stabilizing medical antibacterial oil inspired by the liquid mucus of HG within solid bioinspired OS nanofiber membrane featuring concave array (Fig. 1). Upon immobilization within the solid nanofiber matrix, the medical antibacterial oil may confer anti-adhesion property and dual-modal antibacterial function similar to the skin mucus. Furthermore, the medical oil contained within the concave structures may act as a pressure-responsive deformable gate, capable of selectively opening or closing in response to changes in exudate volume. When excessive exudates accumulate on wound bed, the elevated hydrostatic pressure forces the oil gate to open, allowing excess fluid to pass through. In contrast, under conditions of minimal exudate, the low hydrostatic pressure maintains the oil gate in a closed state, effectively preventing fluid loss. Thanks to this unique exudate-gating mechanism, the bioinspired biphasic dressing can dynamically regulate wound moisture microenvironment. Overall, this bioinspired biphasic design may realize exceptional multifunction, encompassing self-adaptive gating transport, dual-modal antimicrobial activity, and anti-adhesion behavior.

In this study, we first investigate the controlled assembly of the bioinspired “liquid-solid” biphasic dressing. Subsequently, its self-adaptive liquid-gating transport behavior is examined, and the underlying mechanism is systematically elucidated. Moreover, the anti-adhesion property is experimentally validated. The defense-attack dual-modal antibacterial efficacy of the dressing is evaluated. Finally, the therapeutic potential in promoting the healing of infected diabetic wounds is comprehensively assessed.

## 2 Experimental section

### 2.1 Materials

Polycaprolactone (PCL, Mn = 80000) was obtained from Sigma-Aldrich (Germany). N, N-Dimethylformamide (DMF, AR), tetrahydrofuran (THF, AR) and methylene blue were provided by Guangdong Guanghua Sci-Tech Co., Ltd. (China). Thymol was purchased from Shanghai Macklin Biochemical Technology Co., Ltd. (China). Sesame oil was purchased from Luhua Co., Ltd. (China). Vaseline was purchased from Hynaut Co., Ltd. (China). Badger oil was purchased from Xiangjiang Co., Ltd. (China).

### 2.2 Fabrication of the bioinspired biphasic dressing

The bioinspired OS membranes were fabricated via a template-assisted electrospinning technique at room temperature. 0.7 g PCL was added to 3 mL of DMF and 1 mL of THF in a sealed glass vial. The mixture was magnetically stirred at room temperature for 12 h to ensure complete dissolution and obtain a homogeneous, transparent spinning solution. The prepared solution was then transferred to a 5 mL plastic syringe fitted with a stainless-steel needle (18 gauge). The syringe was mounted on a programmable syringe pump with a controlled flow rate of 0.8 mL/h. A high-voltage power supply was connected to the metallic needle, applying a positive DC voltage of 7 kV. The collectors of custom-fabricated circular hole array templates were positioned at a fixed tip-to-collector distance of 10 cm. The circular hole array templates incorporated with varied geometric parameters: (i) hole diameter of 0.3, 0.5, and 0.7 mm; and (ii) hole spacing of 0.6, 1.0, and 1.2 mm. Electrospinning was conducted for a fixed duration of 15 min to deposit PCL nanofibers directly onto the circular hole array template. The as-spun membranes were carefully peeled from the templates and vacuum-dried at room temperature for 24 h to remove residual solvents prior to further use. Subsequently, the bioinspired OS membrane (1.5 × 1.5 cm) was immersed in 10 mL of medical antibacterial oil (thymol/sesame oil = 1:80 g/mL) for 5 s. Excess thymol oil was then removed by suspending the membrane for 4 h, resulting in the bioinspired biphasic dressing. In addition, the control groups were prepared. Briefly, the oil-loaded bioinspired OS membrane (OOM) was prepared by immersing the bioinspired OS membrane into sesame oil without thymol. The thymol-loaded bioinspired OS membrane (TOM) was also prepared by electrospinning of the spinning solution adding 6.1 mg of thymol to the spinning solution mentioned above.

### 2.3 Characterization

The optical microscope (XMA 1000, Chongqing Oupu Optoelectronic Technology, China) was employed to observe both the morphology of the bioinspired biphasic dressing and the bioinspired OS membrane. The field-emission scanning electron microscope (FE-SEM, SU8010, HITACHI, Japan) was utilized to observe the concave structure of the bioinspired OS membrane. Surface topography of the bioinspired OS membrane was investigated by using a profilometer (Dektak XTL, Bruck, Germany).

The bioinspired biphasic dressings (2.5 × 2.5 cm) were applied to the full-thickness dorsal wounds. The dimensions of the dressings were carefully measured on days 1 and 3 to assess dimensional stability. At the same time points, the mass of the dressings was measured to evaluate mass stability. On day 3, the dressings were observed under an optical microscope to examine the stability of concave microstructure and medical oil retention. The animal procedures were reviewed by the Institutional Animal Care and Use Committee of Shanghai Sixth People's Hospital (Approval No.: DWLL2025-0929), ensuring compliance with national principles of animal protection, animal welfare, and ethics.

The optical contact angle measuring instrument (DSA25, Bruck, Germany) was used to characterize the sliding angle of water on the bioinspired biphasic dressing. The bioinspired OS membrane was set as the control group. The dressing was placed on the stage and a 5 μL droplet was dispensed onto the surface. The dynamic contact angle of the bioinspired biphasic dressing was measured using the local tilting method. The sliding angle (θ_r_), advancing angle (θ_A_), and receding angle (θ_R_) of the droplet on the bioinspired biphasic dressing were obtained using the software Photoshop. The hysteresis contact angle (θ_H_) was calculated to evaluate the surface roughness of the bioinspired biphasic dressing.

(1) $$\theta_{H}=\theta_{A}\;‐\;\theta_{R}$$

For liquid absorption and swelling tests, the bioinspired biphasic dressing (2 × 2 cm) was immersed in 10 mL of rhodamine B-stained PBS solution. At predetermined time intervals, the dressing was carefully removed from the solution. The presence of red traces was examined to indirectly reflect the liquid absorption capacity of the dressing. Additionally, the length (l_x_) was measured at each time point. The swelling ratio was calculated using the formula:

(2) $$\text{Swelling}\;\text{ratio}\;\left(\%\right)=\left(l_{x}-l_{1}\right)/l_{1}\times 100\%$$

where l_1_ was the initial length of the dressing.

Transmittance spectra were acquired by using a UV-Vis spectrophotometer (UV1600, Shanghai Jinghua Scientific Instrument Co., China). Both the bioinspired biphasic dressing and the bioinspired OS membrane were cut into rectangular shapes with the size of 3 cm × 1 cm, fixed onto a hollow iron frame and tested with the visible light range (300-800 nm). The mechanical properties of the bioinspired biphasic dressing with the size of 1 cm (width) × 3 cm (length) were evaluated using a universal testing machine (UTM2103, Suns, China). The mean thickness of each sample was determined from measurements at three random locations within the test region using a digital caliper. The initial gauge length L_0_ between the clamps was set to 10 mm. The tensile test was conducted at a speed of 10 mm/min until the sample fractured, with the force (F) - displacement (L) data being acquired simultaneously. The stress-strain curve was derived from the force (F) and displacement (L) data from the universal testing machine. A denoted the effective contact area over which the force (F) was distributed on the object. The tensile strain and tensile stress were calculated as follows:

(3) Tensile strain = L/L_0_

(4) Tensile stress = F/A

The Young's modulus was evaluated from the initial linear elastic portion of the stress-strain curve. This region was subjected to a linear regression analysis in Origin software, and the modulus was calculated as the slope of the resulting fit. Each sample was tested three times.

### 2.4 Liquid-triggered gating transport

To investigate the draining capacity of the bioinspired biphasic dressing, a silicon artificial skin defect (10 × 10 × 2 mm) was prepared. Using a micropipette, 50 μL, 5 μL, and 1 μL of methylene blue-stained PBS solution were precisely deposited at the bottom of the defect. A square dressing (8 × 8 mm) assembled with a hydrophilic layer was carefully placed onto the liquid surface to monitor the liquid transport process. Notably, the dressing was deliberately undersized relative to the defect, ensuring complete contact with droplets without interference from the defect edge. Each experiment was repeated at least three times.

To elucidate the liquid transport pathway from the bioinspired biphasic dressing side to the hydrophilic side, rhodamine B was employed to label the liquid droplet, while curcumin was utilized to mark the bioinspired biphasic dressing. The entire liquid transport process was systematically recorded using a custom-developed fluorescence imaging system. In addition, the transport process from the hydrophilic side to the hydrophobic bioinspired biphasic side was also recorded. Furthermore, the liquid transport from the concave was observed using an optical microscope. To investigate the anti-backflow capacity of the bioinspired biphasic dressing, a full-thickness skin defect was established on the BALB/c mice. 50 μL of methylene blue dye solution was then added to the wound. After the dye solution was absorbed, the area of dye imprinted on the peri-wound was observed.

To quantitatively assess the liquid transport speed, 100 μL of dyed solution was added into the artificial skin defect (10 × 10 × 2 mm). The bioinspired biphasic dressings (8 × 8 mm) with varying concave diameters (0.3, 0.5, and 0.7 mm) and concave spacings (0.6, 1.0, and 1.2 mm) were assembled with a hydrophilic layer and then gently placed onto the liquid surface using a forcep, relying solely on its own gravity. Importantly, the operator's fingers and the forcep were strictly prevented from contacting the top surface of the dressing during the procedure to avoid applying any additional external force, thereby ensuring highly consistent contact pressure between the dressing and the artificial wound across all experiments. The liquid transport time (Δt) was recorded by a smartphone and the average transport speed was calculated using the formula:

(5) v=100/Δt

Different viscous solutions were also prepared to simulate wound exudates with varying viscosities [29]. Briefly, 8.2982 g of sodium chloride and 0.3684 g of calcium chloride dihydrate were dissolved in 1 L of deionized water and stirred at room temperature for 10 min to prepare a standard solution. By adding different masses (1, 33, 54, 69, 75, 88, 105, and 120 mg) of a thickening agent to 10 mL of the standard solution, solutions with different viscosities (1, 10, 20, 30, 40, 50, 60, and 70 mPa s) were obtained. The viscosities of these solutions were measured using a rheometer (MARS60, Thermo Fisher, USA). The liquid transport speed was calculated using the above mentioned formula (5). The above liquid transport experiments were conducted in a temperature- and humidity-controlled environment maintained at 20-25 °C and 40%-60% relative humidity. Each experimental group was repeated at least three times.

To investigate the dynamic moisture regulation function of the bioinspired biphasic dressing, a defect (10 × 10 mm) was created on fresh porcine skin purchased from a local supermarket. To simulate wounds with heavy or minimal exudate, 50 μL or 1 μL of methylene blue-stained PBS solution was applied to the defect, respectively. The initial resistance was measured using a high-sensitivity digital multimeter (Victor VC86E, Shenzhen, China) [30]. Subsequently, the dressing was carefully placed onto the defect without external pressure. After liquid transport completion, the resistance was measured again. This procedure was repeated three times for reproducibility.

### 2.5 *In vitro* cell viability assay

NIH 3T3 mouse fibroblasts (Beyotime Biotechnology Co. Ltd., China) and the bioinspired biphasic dressing were co-cultured into 96-well plates at 1 × 10^4^ cells in high-glucose Dulbecco's Modified Eagle Medium (DMEM) with 10% fetal bovine serum (FBS). Cells were incubated at 37 °C in a 5% CO_2_ environment for 24 h. Cell viability was assessed by the CCK-8 assay kit (Beyotime Biotechnology Co. Ltd., China). The cell viability was calculated as follow:

(6) $$\text{Cell}\;\text{viability}\;\left(\%\right)=\frac{{\text{Experimental}\;\text{group}}_{\text{OD}450}}{{\text{Blank}\;\text{control}}_{\text{OD}450}}\times 100\%$$

### 2.6 Bacteria and biofilm preparation

Both *E. coli* and *S. aureus* were preserved at −80 °C before use. Before each experiment, strains were revitalized overnight on blood agar plates and incubated in 5 mL tryptic soybroth (TSB, Haibo, Qingdao, China) or TSBG (TSB with 0.5% glucose, for biofilm experiments) for 24 h at 37 °C. Subsequently, the bacterial solutions were fine-tuned to the required concentrations in TSB or PBS for antibacterial experiments.

### 2.7 *In vitro* “defense-attack” synergistic antibacterial performance

The bioinspired biphasic dressing with the size of 1 cm × 3 cm was placed on a glass slide inclined at 23°. The commercial anti-adhesive Vaseline gauze™ and the bioinspired OS membrane were used as control groups. 35 μL of rhodamine B solution was dripped onto the membrane and the sliding behavior of the dye solution was observed and recorded. The Confocal Laser Scanning Microscope (CLSM, A1, Nikon, Japan) was used to quantify the contaminant area. Surface energy was determined using the Owens-Wendt method.

According to the CLSI guidelines, the microdilution method was employed to determine the minimum inhibitory concentration (MIC) of thymol solution against *S. aureus* and *E. coli*. Thymol solution was serially diluted in Mueller-Hinton broth (MHB) and co-cultured with bacteria in 96-well plates. Sterile MHB was used as a negative control. The MIC was defined as the lowest concentration that inhibited visible bacterial growth.

For the investigation of thymol release, bioinspired biphasic dressing and antibacterial TOM dressing (1.5 × 1.5 cm) were immersed in 10 mL of ultrapure water within test tubes. The samples were continuously agitated in an orbital shaker at 37 °C and 100 rpm for 24 h. At predetermined timepoints of 4, 6, 12, and 24 h, 5 mL of the released solution were collected for subsequent analysis. Notably, following each sampling event, equivalent volumes of ultrapure water were replenished to ensure constant volume conditions throughout the release testing process. The absorbance of the released solution was measured at 272 nm, and the concentration of thymol released into the aqueous phase was subsequently quantified using a pre-established standard curve.

The *in vitro* antibacterial activity of the bioinspired biphasic dressing was evaluated against *E. coli* and *S. aureus* using the colony-forming unit (CFU) plating method. In brief, bacterial suspensions (10^6^ CFU/mL) were co-incubated with the bioinspired biphasic dressing at 37 °C for 24 h in sterilized TSB. Then, 100 μL of the bacterial suspension was spread onto blood agar plates. After 18 h of incubation, the number of colonies on the plates was evaluated to access the effect of bacterial growth inhibition.

Biofilms were formed on a 24-well plate containing the bioinspired biphasic dressing by adding bacterial suspension, followed by incubation with TSBG at 37 °C for 24 h. Non-adherent bacteria were then removed by gently rinsing three times with PBS. The resulting biofilm was examined for three-dimensional structure and bacterial viability using CLSM. In brief, the biofilm was stained with a LIVE/DEAD BacLight Bacterial Viability Kit (Invitrogen, USA) and incubated in the dark at room temperature for 30 min. Excess dye was then removed with PBS. Live bacteria fluoresced green, while dead bacteria fluoresced red. For crystal violet assay, the biofilm was fixed with methanol and stained with a 0.2% crystal violet solution at room temperature for 30 min. After gently washing with PBS to remove excess dye, the stained biofilm was pictured. The biofilm inhibition rate of each group was quantitatively analyzed by CFU plating method.

### 2.8 Anti-adhesion performance evaluation

40% (w/v) gelatin solution was poured into the polytetrafluoroethylene (PTFE) molds to simulate the viscous exudates. The bioinspired biphasic dressing with the size of 2 cm × 3 cm was then applied to the gelatin solution surface at 37 °C for 15 min. Both the commercial gauze and the commercial anti-adhesive Vaseline gauze™ with the same size were used as control groups. After removing the dressings, the gelatin residue on these dressing was observed. A 90° peeling test was conducted at a speed of 10 mm/min by using a universal testing machine to quantitatively characterize the interfacial adhesive force. The peeling energy was calculated as follow, whereas F was the peeling force and b represented the width of the dressing.

(7) $$\text{Pe}e\text{ling}\;\text{energy}=\frac{2F}{b}$$

*In vivo* anti-adhesion properties were evaluated by creating wound with the diameter of 1 cm on the back of mice. The bioinspired biphasic dressing was applied to the wound. Both the commercial gauze and the commercial anti-adhesive Vaseline gauze™ were used as control groups. After 30 min of coverage, the peeling curves of the three groups were obtained with the aid of the universal testing machine. The gradual detachment of the dressings after 4 days of coverage was also observed. Further, the bioinspired biphasic dressing was repeatedly peeled 100 times from the skin of mice. The peeled skin tissue was harvested, fixed in 4% paraformaldehyde, dehydrated, and embedded in paraffin. Sections of 4 μm thickness were prepared using a Leica SP1600 microtome (Leica, Germany). Hematoxylin and Eosin (H&E) staining was performed to examine the inflammation in the local skin.

### 2.9 Synergistic mechanism among the three functions

To study the effect of liquid transport speed on thymol delivery, 200 μL of PBS was added to the porcine skin defect. The bioinspired biphasic dressings with different liquid transport speeds (1.21, 11.20, and 13.68 μL/s) were assembled with a hydrophilic layer to form a bilayered structure. The bilayered dressing was carefully placed onto the porcine skin defect. The thymol release concentration was subsequently quantified using a UV-Vis spectrophotometer. To further investigate whether the medical oil interfered with the contact between thymol and bacteria, the bioinspired biphasic dressing (the OS membrane loaded with medical oil and thymol) and the control group (the OS membrane loaded with PBS and thymol) were gently placed onto pre-established *S. aureus* and *E. coli* biofilms, followed by co-cultivation and subsequent CFU analysis.

### 2.10 *In vivo* bacteria-infected diabetic wound healing

To establish the infected diabetic wound model, healthy male BALB/c mice (8 weeks old) were intraperitoneally injected with streptozotocin (STZ) at a dose of 50 mg/kg for five consecutive days. Two weeks post-injection, mice with blood glucose levels ≥16.7 mmol/L were confirmed as diabetic. Following anesthesia, the dorsal skin was shaved and disinfected, and a full-thickness circular wound was created on the back. Subsequently, a 50 μL suspension of *S. aureus* (1 × 10^6^ CFU/mL) was inoculated into the wounds to induce infection. The bioinspired biphasic dressing, featuring exudate-triggered gating transport and dual-modal “defense-attack” antibacterial performance, was carefully applied to the wounds. Additionally, exudate management dressings (the NOD dressing with continuous exudate transport capability and the NCD dressing without exudate transport function) and antibacterial dressings (the OOM dressing with single-defense antibacterial activity and the TOM dressing with single-attack antibacterial activity) were used as controls. Moreover, four commercial dressings (a Vaseline gauze dressing™, a hydrogel dressing™, a foam dressing™, and a silver-containing dressing™) and the pristine bioinspired OS membrane were also included as control groups. Digital images were captured on days 0, 3, 7, 10, and 14 post-surgeries to monitor infection status and calculate wound healing rates across different treatment groups.

### 2.11 Histochemical staining analysis

At designated time points, mice from each group were euthanized and wound skin tissues from days 3, 7, and 14 were harvested and fixed in 4% paraformaldehyde. The tissues were then dehydrated and embedded in paraffin. Sections of 4 μm thickness were prepared by using a microtome. H&E staining and Masson's trichrome staining were performed. The optical microscope (Leica, Germany) was used to observe the pathological sections, assessing epithelialization, collagen deposition, and hair follicle number in the wound skin tissues.

### 2.12 Assessment of wound tissue water content

Wound tissue samples were collected from all groups on days 3, 7, and 14. Wet tissue weights were measured using an electronic balance, followed by drying at 60 °C for 48 h to determine dry weights. Normal mouse skin tissues were served as the control group.

### 2.13 Immunofluorescence analysis

Wound skin tissue sections collected on day 3 were subjected to immunofluorescence staining. The sections were incubated with primary antibodies against TNF-α, IL-6, CD31, α-SMA, CD3, F4/80 and Ly6G, followed by incubation with corresponding fluorescently labeled secondary antibodies. The cell nuclei were stained with DAPI. The stained sections were imaged using the fluorescence microscope (Leica, Germany), and quantitative analysis was performed using the software Image J.

### 2.14 *In vivo* antibacterial performance

Wound exudate was collected from all groups of mice on days 3 and 7 to assess bacterial load. Specifically, the exudate was diluted and cultured in sterilized TSB at 37 °C for 6 h. Then, 100 μL of each TSB solution was spread onto blood agar plates. Bacterial colonies were counted after 18 h of incubation at 37 °C. Poly-N-acetylglucosamine (PNAG), a major component of the extracellular polysaccharide matrix in *S. aureus* biofilms, can be specifically bound by wheat germ agglutinin (WGA), allowing for its quantitative detection. Briefly, tissue samples were homogenized, and biofilm extracellular polysaccharides were extracted. The extracts were then incubated with FITC-conjugated WGA in the dark for 10 min, and the fluorescence intensity was measured using a microplate reader.

### 2.15 Statistical analysis

All experiments were repeated at least three times, and the results were subjected to statistical analysis. Data was presented as mean ± standard deviation. Statistical analysis was performed using GraphPad Prism 8. One-way ANOVA and two-sided Student's t-tests were used to assess statistical significance. A p-value ≤0.05 was considered statistically significant and denoted as ∗; p ≤ 0.01 was considered highly significant and denoted as ∗∗; p ≤ 0.001 was considered very highly significant and denoted as ∗∗∗.

## 3 Results and discussion

### 3.1 Preparation and characterization of the bioinspired biphasic dressing

**Fig. 2:**
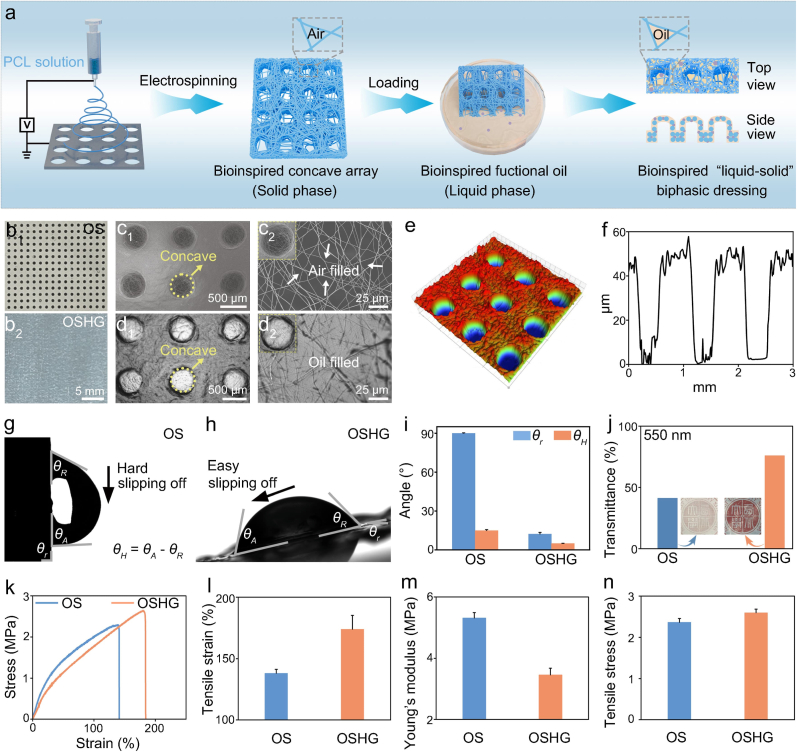
Preparation and characterization of the bioinspired “liquid-solid” biphasic OSHG dressing. (**a**) Schematic diagram of preparing the bioinspired biphasic dressing. Photos of (**b1**) the bioinspired OS membrane and (**b2**) the bioinspired biphasic dressing. Images of the bioinspired concave structure of (**c1**) the bioinspired OS membrane and (**d1**) the bioinspired biphasic dressing. Images of a single concave of (**c2**) the bioinspired OS membrane and (**d2**) the bioinspired biphasic dressing. (**e**) Three-dimensional image and (**f**) cross-sectional profile of the bioinspired OS membrane. Photos of sliding angle (θ_*r*_) of (**g**) the bioinspired OS membrane and (**h**) the bioinspired biphasic dressing. (**i**) Statistical analysis of hysteresis contact angle (*θ_H_*) (n = 3). (**j**) Transmittance of the bioinspired OS membrane and the bioinspired biphasic dressing at 550 nm. The insets were photos of the bioinspired OS membrane and the bioinspired biphasic dressing on a red logo. (**k**) Stress-strain curves of the bioinspired OS membrane and the bioinspired biphasic dressing. (**l**) Tensile strain, (**m**) Young's modulus, and (**n**) tensile stress of the bioinspired OS membrane and the bioinspired biphasic dressing (n = 3).

In this study, we first prepared the bioinspired OS membrane composed of the PCL nanofibers by template assisted electrospinning technology, then infused with thymol loaded sesame oil (medical antibacterial oil) to obtain the bioinspired biphasic dressing (Fig. 2a). The results revealed that the bioinspired OS membrane featured concave array structure and small pores were also observed among the nanofibers (Fig. 2b1, 2c1 and 2c2). The unique 3D concave array structure of the bioinspired OS membrane was further verified using surface profilometry (Fig. 2e and f). After being modified with the medical antibacterial oil, the concave arrays could be also observed in the bioinspired biphasic dressing, and the medical oil was found to be filled into the pores within the bioinspired OS membrane (Fig. 2b2, 2d1 and 2d2). We further investigated the stability of medical oil within the bioinspired biphasic dressing. The results indicated that approximately 70% of the medical oil remained in the bioinspired biphasic dressing after immersion in PBS solution for 72 h (Figs. S1 and S2). In this study, the dressing was changed every three days. The high oil retention rate observed at 72 h reflected the oil retention performance of the dressing throughout the 14-day treatment, thereby ensuring continuous functions throughout the entire treatment period. The dressing stability on rat dorsal wounds was also studied. The results indicated that the dressing maintained consistent structure, favorable mass stability, and stable retention of medical oil on rat dorsal wounds for 3 days (Figs. S3 and S4). The bioinspired OS membrane was made by lipophilic PCL, due to the lipophilic nature and porous structure of the bioinspired PCL nanofiber membrane, the medical oil was able to wet the nanofibers and easily fill into the small pores by capillary forces [31]. As the confinement effect of the small pores, the bioinspired OS membrane showed strong affinity to the medical oil.

We then carefully evaluate the lubrication property of the bioinspired biphasic dressing. The results indicated that, compared with the bioinspired OS membrane without loading medical oil, the bioinspired biphasic dressing exhibited significantly lower sliding angle and hysteresis contact angle, respectively (Fig. 2g–i). This was attributed to the medical oil reducing the interfacial resistance between water droplet and the dressing surface [32]. Owing to the lubricating effect of the dressing and the immiscibility between the medical oil and water, the bioinspired biphasic dressing exhibited excellent liquid repellency (Fig. S5a). Concurrently, its dimensions remained stable throughout 72 h of incubation in PBS without significant swelling (Fig. S5b).

Unexpectedly, the bioinspired biphasic dressing exhibited greater transparency compared to the bioinspired OS membrane. As shown in Fig. 2j, the red logo was clearly visible through the bioinspired biphasic dressing, whereas it was obscured through the bioinspired OS membrane. Besides, it was also found that, the bioinspired biphasic dressing demonstrated a higher transmittance than the bioinspired OS membrane at 550 nm (Fig. S6). The reason was that after the medical oil was infused into the pores, light scattering was significantly diminished, thereby enabling light to pass through the bioinspired biphasic dressing [33]. The high transparency characteristic of the bioinspired biphasic dressing enabled medical professionals to monitor the wound in real-time, thereby ensuring timely and appropriate care. In addition, it was also observed that the bioinspired biphasic dressing exhibited better mechanical property than the bioinspired OS membrane (Fig. 2k–n). It was reported that the medical oil exhibited plasticity, which could enhance the mobility of molecular chains [34]. This characteristic made the nanofiber membrane more susceptible to plastic deformation under external force, thereby improving its mechanical property. This excellent mechanical property made the dressing not only suitable for static wounds, but also suitable for active joint wounds. Overall, owing to the integration of the bioinspired OS membrane and bioinspired medical antibacterial oil, the bioinspired “liquid-solid” biphasic dressing demonstrated exceptional lubrication property, high transparency, and superior mechanical performance.

### 3.2 Self-adaptive liquid-gating transport performance

**Fig. 3:**
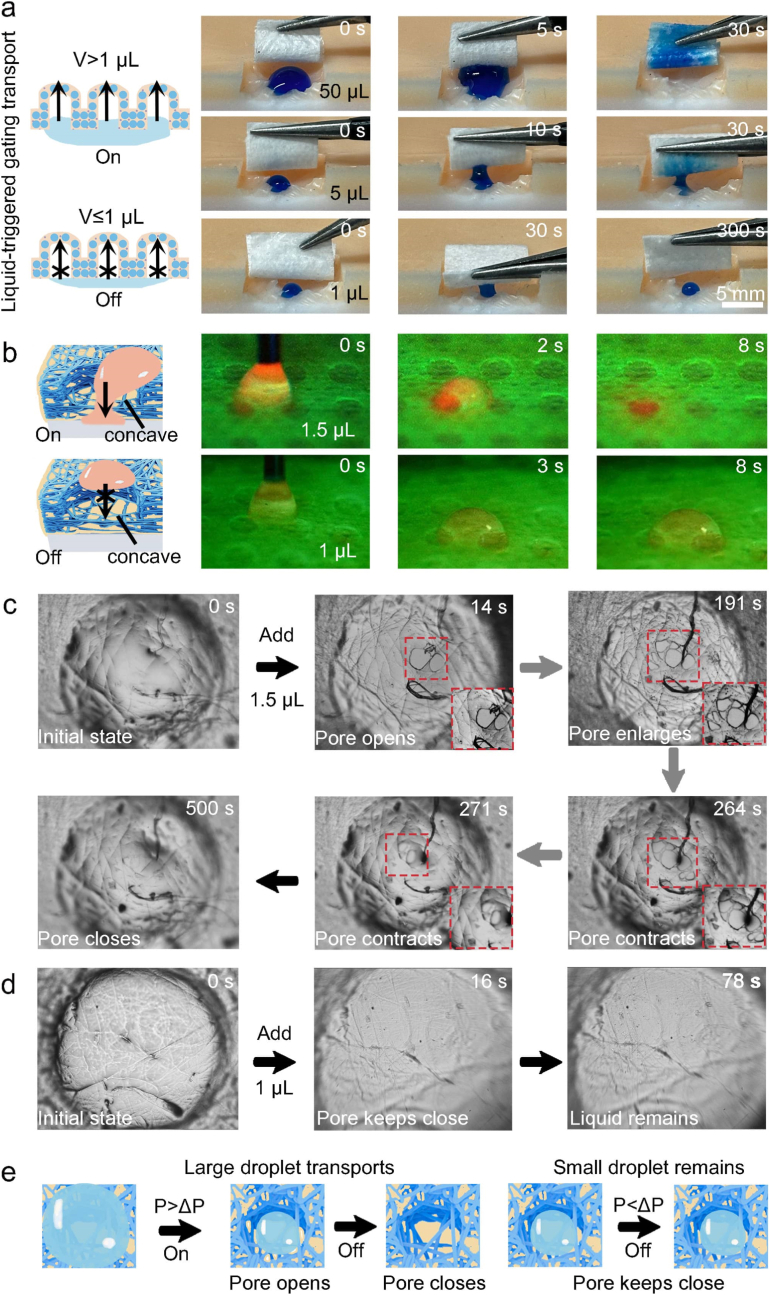
The self-adaptive liquid-gating transport performance of the bioinspired biphasic OSHG dressing. (**a**) The bioinspired biphasic dressing could drain large liquid droplet while hindering small liquid droplet. (**b**) Transport pathway of a large liquid droplet and a small liquid droplet from the bioinspired biphasic dressing, respectively. The detailed transport process of (**c**) a large liquid droplet and (**d**) a small liquid droplet from a single concave, respectively. (**e**) Mechanism of liquid-triggered gating transport performance. Prior to the investigation of liquid transport process, the bioinspired biphasic dressing was assembled with a hydrophilic commercial nonwoven fabric to form bilayered structure.

Both excessive and insufficient exudates hindered healing process; thus, controlling wound moisture balance and maintaining a mildly moist microenvironment was proven to be beneficial for wound healing. It was of great importance to develop dressings that could both remove excessive exudates and retain tiny amounts of exudate in the wound. Thus, we first focused on investigating how liquid volumes influenced the draining process of the bioinspired biphasic OSHG dressing. The bioinspired biphasic dressing with high hydrophobicity was assembled with a hydrophilic commercial nonwoven fabric to form bilayered structure, then water droplets with different volumes were dripped onto the bioinspired biphasic dressing. The results revealed when the droplet volume was 50 μL, the bioinspired biphasic dressing could rapidly drain the liquid within 30 s. By contrast, the bioinspired biphasic dressing could not drain the liquid with volume less than 1 μL (Fig. 3a). These results indicated that liquid volume was the critical factor to determine the draining ability of the bioinspired biphasic dressing.

To more clearly observe the transport pathway of liquid from the bioinspired biphasic dressing, we labeled the droplets with red dye and the bioinspired biphasic dressing with green dye, respectively. Subsequently, we utilized the microscopy to examine the detailed liquid transport pathway under ultraviolet light irradiation. As depicted in Fig. 3b and Movie S1, S2, when the volume surpassed 1 μL, the red droplet was able to traverse the concave structure of the bioinspired biphasic dressing and enter the underlying hydrophilic layer. While when the volume was less than 1 μL, the small droplet remained stationary on the surface of the bioinspired biphasic dressing and was unable to pass through the concave structure. We further deeply observed the liquid transport profile in a single concave. As depicted in Fig. 3c and Movie S3, the concave was initially filled with medical oil. Upon dripping a larger droplet exceeding 1 μL, pores began to open within the concave and progressively expanded after 14 s, enabling the liquid to gradually permeate through the concave structure. Interestingly, once the liquid was entirely transported, pores in the concave gradually closed. In contrast, when a small droplet with a volume of less than 1 μL was applied, no pores were observed within the concave, and the droplet remained intact after 78 s (Fig. 3d and Movie S4). Overall, these results demonstrated that the bioinspired biphasic dressing exhibited unique liquid-triggered gating transport function (Fig. 3e).

Supplementary data related to this article can be found online at https://doi.org/10.1016/j.bioactmat.2026.04.001

The following are the Supplementary data related to this article:

Multimedia component 2 Multimedia component 2

Multimedia component 3 Multimedia component 3

Multimedia component 4 Multimedia component 4

Multimedia component 5 Multimedia component 5

To further elucidate the underlying mechanism of the liquid-triggered gating transport property, the Laplace pressure theory was proposed to analyze the forces exerted by droplet on the oil-infused concave cavity. The physical model was constructed based on the following assumptions [35]: (1) The nanofibers exhibited a rigid morphology with intact structural integrity; (2) The pore size distribution and nanofiber surface wettability demonstrated uniform characteristics; (3) The pores within the nanofiber membrane could be approximated as a uniform array of capillaries.

The liquid-gating transport process was determined by two forces, including the motivated hydrostatic force (P) generated by water and the resistant transmembrane pressure (ΔP) caused by oil [36].

The transmembrane pressure (ΔP) was corresponded to the Laplace pressure theory:

(8) $$\Delta P=\frac{2\gamma \left|\cos\;\theta_{\alpha }\right|}{r}$$

where r was the pore size, γ was the interfacial tension between the oil and water, and θ_α_ was the contact angle of water on the bioinspired biphasic dressing.

The hydrostatic pressure (P) generated by liquid droplet could be obtained by formula 9:

(9) P=ρgh

where ρ was the water density, g was gravitational acceleration, and h was the height of the liquid droplet.

As shown in Fig. S7, we could build the relationship between the height (h) and volume (V) of the droplet:

(10) $$h={\left[\frac{2+\cos\;\theta }{3\pi \left(1‐\cos\;\theta \right)}\right]}^{\frac{1}{3}}{\;V}^{\frac{1}{3}}$$

where θ was the cone angle between the central axis and the droplet boundary to the sphere's center. The detailed derivation could be found in the Supporting information.

Combined with (9), (10), formula 11 could be deduced:

(11) $$P={\left[{\left(\frac{2+\cos\;\theta }{3\pi \left(1‐\cos\;\theta \right)}\right)}^{\frac{1}{3}}\rho g\right]\;V}^{\frac{1}{3}}$$

where θ was the cone angle between the central axis and the droplet boundary to the sphere's center, ρ was the water density, and g was gravitational acceleration.

According to formula 11, hydrostatic pressure (P) was directly proportional to the volume (V) of the liquid droplet. As the liquid volume (V) increased, hydrostatic pressure (P) increased and eventually exceeded the transmembrane pressure (ΔP), thereby activating the opening of the pores in the concave and enabling water transport. Furthermore, owing to the high mobility of the oil and the capillary action, the oil could refill the pores, thereby causing the pores to close [37]. For the small droplet, the hydrostatic pressure (P) was smaller than the transmembrane pressure (ΔP), and water droplet was unable to push away the oil. Consequently, the pores in the concave remained closed and the liquid could not pass through the concave.

As the bioinspired biphasic dressing was capable of draining significant amounts of fluid, we further investigated the effect of concave structure on liquid transport speed. The results demonstrated that the bioinspired biphasic dressing with concave structure exhibited a liquid transport speed twice as fast as that of the flat dressing without concave structure (Fig. S8). The results also indicated that the liquid transport speed increased with an increase in concave diameter and a decrease in concave spacing (Figs. S9 and S10). The fast liquid transport speed was governed by a concave-mediated parallel channel flow mechanism. Geometrically, the concave array partitioned the bulk liquid into discrete microdroplets, with each microdroplet entering an individual concave cavity to establish parallel transport pathways. According to the principle of parallel hydraulic resistance, the total flow rate increased with channel number. Consequently, this parallel channel architecture enabled multi-cavity collaborative transport, resulting in fast liquid transport speed.

Then, we explored the influence of medical oil on liquid transport speed. The bioinspired biphasic dressing loading with sesame oil exhibited the fastest transport speed. When loading with vaseline, the bioinspired biphasic dressing failed to drain liquid (Fig. S11). The viscosity of the medical oil might be the key factor affecting the transport speed [38,39], shown in Fig. S12.

Wound exudate composition is complex, and in addition to serous exudate, viscous exudate is also commonly encountered in clinical practice. To more accurately mimic the actual wound microenvironment, we investigated the effect of exudate viscosity on liquid transport speed. The results demonstrated that transport speed decreased progressively with increasing liquid viscosity, from 11.99 μL/s at 1 mPa s to 0.84 μL/s at 40 mPa s (Fig. S13). Transport persisted at 50-60 mPa s, but failed completely at 70 mPa s. Thus, the bioinspired biphasic dressing effectively transported exudates with viscosities up to 60 mPa s.

We further investigated the anti-backflow performance of the bioinspired biphasic dressing. As illustrated in Figs. S14 and S15 and Movie S5, S6, when a large droplet was deposited onto the hydrophobic bioinspired biphasic dressing side, it could be effectively transported through the concave structures from the hydrophobic bioinspired biphasic dressing layer to the hydrophilic layer. Conversely, when droplet was deposited onto the hydrophilic layer, it only diffused laterally within this layer and was unable to penetrate the hydrophobic bioinspired biphasic dressing layer. To further validate the anti-backflow capability of the bioinspired biphasic dressing under conditions involving large volumes of liquid, a rat wound model was employed. PBS stained with methylene blue was introduced into the wound bed. After treatment with conventional gauze, a prominent blue imprint was observed around the wound edge (Fig. S16), indicating a significant risk of peri-wound maceration due to lateral liquid diffusion within the gauze. Interestingly, no such blue trace was detected around the wound edge when the bioinspired biphasic dressing was utilized (Fig. S17). These findings demonstrated that the bioinspired biphasic dressing effectively prevented liquid backflow.

Supplementary data related to this article can be found online at https://doi.org/10.1016/j.bioactmat.2026.04.001

The following are the Supplementary data related to this article:

Multimedia component 6 Multimedia component 6

Multimedia component 7 Multimedia component 7

Finally, we validated the dynamic moisture regulation function of the bioinspired biphasic dressing by employing bioelectrical impedance to monitor moisture levels in porcine skin defects. The results demonstrated that for porcine skin defect with 50 μL of PBS, the initial resistance was 97 kΩ; following dressing application, resistance significantly increased to 2518 kΩ. In contrast, for simulated wound with 1 μL of PBS, the initial resistance was 2511 kΩ, and resistance remained stable without significant change after dressing coverage (Figs. S18 and S19). These findings indicated that, under high-exudate condition, the dressing effectively reduced wound moisture through active liquid drainage, achieving an optimal moist state; whereas under low-exudate condition, the dressing preserved minimal exudate without removal, preventing excessive wound desiccation. The 1 μL of exudate could be uniformly distributed across a 1 cm^2^ wound surface, forming a water film approximately 10 μm thick. This thickness sustained a continuous, physiologically hydrated microenvironment that actively promoted keratinocyte migration, preserved the bioactivity of endogenous growth factors, and supported re-epithelialization [40].

### 3.3 Defense-attack synergistic antibacterial performance

Good biocompatibility is a primary requirement for the application of wound dressings. Therefore, prior to assessing antibacterial performance, the effect of the bioinspired biphasic dressing on cell viability was evaluated using the CCK-8 assay. As shown in Fig. S20, after co-cultivation with the bioinspired biphasic dressing, high survival rate was observed in NIH 3T3 cells, indicating the excellent biocompatibility of the dressing.

Exuding wounds are frequently associated with severe bacterial infection, whereas single antibacterial mode is insufficient to effectively suppress bacterial growth. Consequently, the bioinspired biphasic dressing incorporating medical antibacterial oil with both lubrication and antibacterial property was designed to exhibit dual-modal antibacterial properties. The lubrication characteristic of the bioinspired biphasic dressing might potentially prevent bacterial adhesion as a defensive mechanism, while the antibacterial agent thymol in the medical oil might release and eliminate bacteria as an offensive mechanism, thereby achieving the desired synergistic defense-attack dual-modal antibacterial effect.

**Fig. 4:**
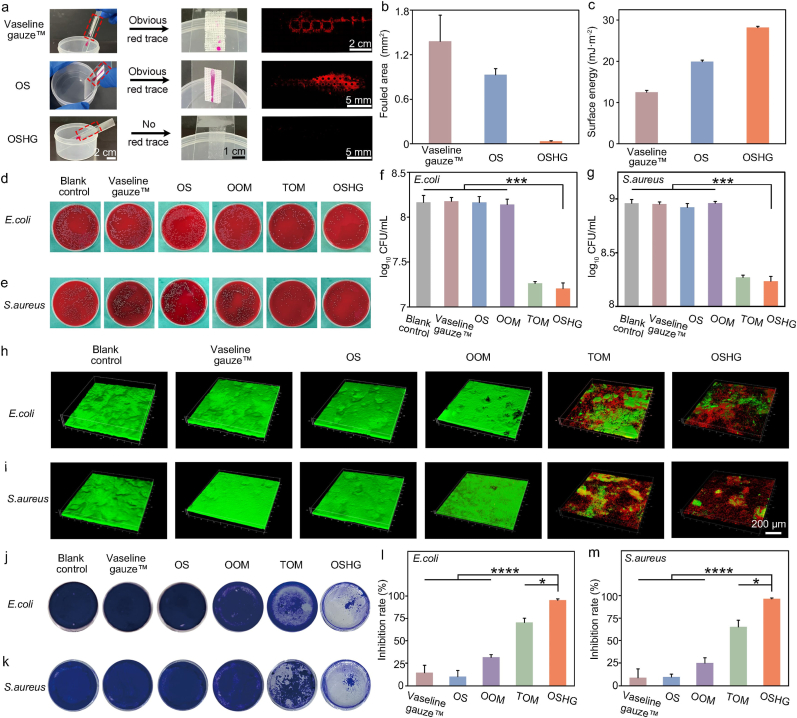
“Defense-attack” dual-modal synergistic antibacterial performance of the bioinspired biphasic OSHG dressing. (**a**) Lubrication feature of the commercial anti-adhesive Vaseline gauze™, the bioinspired OS membrane, and the bioinspired biphasic dressing, respectively. (**b**) Statistical analysis of the contaminant area (n = 3). (**c**) Surface energy of the three different dressings. Representative blood agar plate images of the (**d**) *E. coli* and (**e**) *S. aureus* after different treatment, respectively. The quantitative data of the growth of (**f**) *E. coli* and (**g**) *S. aureus* after different treatment, respectively (n = 3). Reconstructed 3D images of (**h**) *E. coli* and (**i**) *S. aureus* through Live/Dead (green/red) staining, respectively. The crystal violet staining images of (**j**) *E. coli* and (**k**) *S. aureus*. The biofilm inhibition rate of (**l**) *E. coli* and (**m**) *S. aureus* after different treatment, respectively (n = 3). (OOM: The bioinspired OS membrane loading only with medical oil. TOM: The bioinspired OS membrane loading only with thymol. OSHG: The dressing loading with both medical oil and thymol).

Firstly, rhodamine B, known for its strong staining property, was applied to various samples to evaluate their lubrication-associated anti-fouling capability. As shown in Fig. 4a, significant red residues were observed on the commercial anti-adhesive Vaseline gauze™ and the bioinspired OS membrane, whereas no such residue was detected on the bioinspired biphasic dressing. Quantitative analysis further confirmed that the contaminated area on the bioinspired biphasic dressing was minimal, accounting for only 3% of that on the commercial anti-adhesive Vaseline gauze™ and 2% of that on the bioinspired OS membrane (Fig. 4b). The low surface energy of the bioinspired biphasic dressing contributed to its superior anti-fouling performance (Fig. 4c) [41]. The unique anti-fouling property could effectively inhibit bacterial adhesion.

Then, the thymol-based attack antibacterial model was investigated. The MIC of thymol solution against *S. aureus* and *E. coli* was measured, as shown in Fig. S21. Additionally, the medical oil was found to promote dissolution of lipophilic thymol, which facilitated its rapid release and consequent antibacterial activity (Fig. S22). We then found that the antibacterial properties of the bioinspired biphasic dressing were much better than those of dressing OOM, and slightly better than those of dressing TOM (Fig. 4d–g). Previous studies had indicated that thymol was capable of disrupting the integrity of bacterial cell membranes, enhancing their permeability, and leading to the efflux of critical intracellular components such as proteins, nucleic acids, and nutrients. In this study, as the OOM dressing was not impregnated with thymol, and therefore did not exhibit any antimicrobial activity. By contrast, as both the bioinspired biphasic dressing and the TOM dressing could release thymol, favoring for achieving good antibacterial effect.

Finally, the anti-biofilm formation capability of the bioinspired biphasic dressing was systematically investigated. The CLSM imaging revealed that, compared with the bioinspired OS membrane, the OOM group revealed observable biofilm defects, confirming the biofilm-loosening effect of medical oil. In contrast, the TOM group exhibited superior anti-biofilm ability, as evidenced by large-area biofilm defects and red-stained dead bacteria. Staining images of the bioinspired biphasic group revealed intense red fluorescence, a thinner structure, and a more dispersed distribution of the biofilm, indicating significant disruption of its formation (Fig. 4h and i). Furthermore, both the crystal violet staining results and CFU analysis confirmed the superior anti-biofilm activity of the bioinspired biphasic group compared to the OOM and TOM groups (Fig. 4j–m). To elucidate the underlying mechanism, we quantified antibacterial efficacy against both biofilm-embedded bacteria and their planktonic counterparts within the biofilm system. As shown in Fig. S23 and Fig. 4j–m, medical oil alone did not reduce the total bacterial burden but decreased biofilm-embedded bacteria while increasing the planktonic fraction, indicating biofilm disruption without direct bactericidal activity. Thymol alone reduced the total bacterial count, but showed limited efficacy against biofilm-embedded bacteria. Notably, co-treatment with medical oil and thymol markedly reduced both biofilm-embedded and planktonic bacteria, resulting in a significantly lower total bacterial burden than thymol alone, indicative of a synergistic antibacterial effect. This effect was attributed to the ability of medical oil to enhance the antibacterial activity of thymol, through improving its solubility and loosening the biofilm structure, thereby increasing its efficacy against biofilm-associated bacteria.

### 3.4 Anti-adhesion performance

**Fig. 5:**
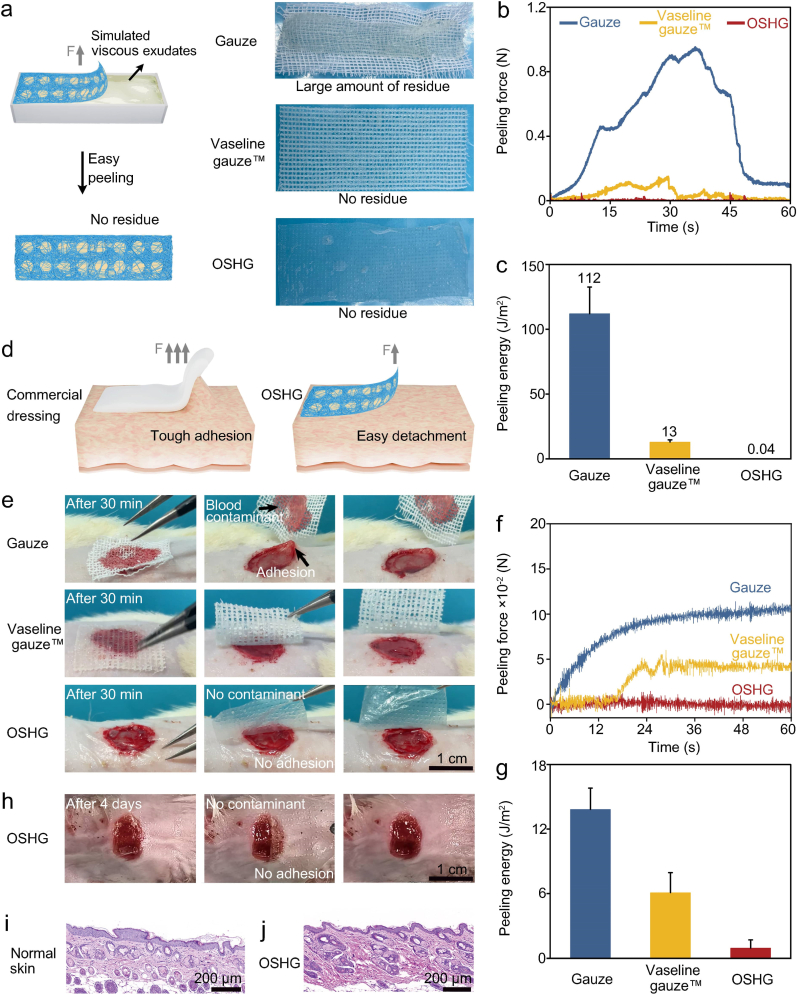
The anti-adhesion performance of the bioinspired biphasic OSHG dressing. (**a**) Appearance of the bioinspired OS membrane, commercially available anti-adhesive Vaseline gauze™, and the bioinspired biphasic OSHG dressing after being peeled from the simulated viscous exudates, respectively. Peeling force of the dressings when in contact with (**b**) simulated viscous exudates and (**f**) mice wounds (n = 3). Peeling energy of the dressings when in contact with (**c**) simulated viscous exudates and (**g**) mice wounds (n = 3). (**d**) Schematic illustration of the strong adhesion by the commercial dressing and the facile detachment achieved by the bioinspired biphasic dressing. Photos of the peeling process (**e**) after 30 min of contact and (**h**) following 4 days of contact. H&E staining of (**i**) the normal skin and (**j**) the skin after 100 cycles of repeated peeling.

Exuding infected wounds, characterized by slow healing, required repeatedly dressing exchanges. However, exudate was rich in viscous substances such as fibrin, which could cause dressings to adhere tightly to the wound [42], thus dressings with anti-adhesion property were highly desirable. To test the *in vitro* anti-adhesion effect, gelatin solution was used to simulate viscous exudates, as gelatin possessed an interface adhesion characteristic similar to exudates due to its extensive hydrogen bond network [43,44]. As shown in Fig. 5a, significant gelatin residue was remained after removing the commercial gauze, indicating that the peeling process caused damage on the wound surface. In contrast, no gelatin residue was observed on either commercial anti-adhesive Vaseline gauze™ or the bioinspired biphasic OSHG dressing, demonstrating their good anti-adhesion property. Statistical analysis revealed that the bioinspired biphasic dressing exhibited the lowest peeling force and peeling energy compared to both gauze and commercial anti-adhesive Vaseline gauze™ (Fig. 5b, c, 5d).

To evaluate the *in vivo* anti-adhesion performance, a full-thickness skin defect model was established on mice back. After covering for 30 min, the bioinspired biphasic dressing was removed from the wound. Fig. 5e showed that the gauze exhibited tough adhesion and the removal process resulted in deformation of the wound edge. In contrast, both commercial Vaseline gauze™ and the bioinspired biphasic dressing were easily removed. The bioinspired biphasic dressing also exhibited the lowest peeling energy, with a value of only 0.94 J/m^2^, which was significantly lower than that of the gauze and the anti-adhesive Vaseline gauze™ (Fig. 5f and g). Furthermore, even after prolonged coverage of 4 days, the bioinspired biphasic dressing remained easily removable (Fig. 5h). We further compared the *in vivo* peeling energy of the bioinspired biphasic dressing with that of previously reported anti-adhesion dressings. The results indicated that the peeling energy of our dressing (0.94 J/m^2^) was significantly lower than those of bilayered dressings (>40 J/m^2^) and foam dressings (>120 J/m^2^) (Table S1), demonstrating the superior anti-adhesion performance of our bioinspired dressing.

To further investigate whether long-term repeated adhesion and detachment of the bioinspired biphasic dressing would induce local inflammatory responses, a repeated peeling experiment was conducted on mouse dorsal skin. The results indicated that no visible damage to the skin was observed after 100 repeated peeling cycles (Fig. S24). Additionally, H&E staining analysis further confirmed that the repeated adhesion and detachment process did not cause inflammatory reactions in the local skin tissue (Fig. 5i and j).

The synergistic mechanism among the three functions (liquid transport, antibacterial, and anti-adhesion) was carefully analyzed. We first investigated the effect of liquid transport speed on thymol delivery for antibacterial purposes. The results indicated that the three bioinspired biphasic dressings, with liquid transport speeds of 1.21, 11.20, and 13.68 μL/s, achieved drug delivery percentages to the simulated wound bed of 71.47%, 77.48%, and 82.76%, respectively (Fig. S25). This result demonstrated that faster liquid transport speed favored higher thymol delivery efficiency at the wound bed. To further investigate whether the medical oil for anti-adhesion interferes with the contact between thymol and bacteria, we evaluated the antimicrobial efficacy of two membranes: bioinspired biphasic OSHG (the OS membrane loaded with medical oil and thymol) and the OS membrane loaded with PBS and thymol. The results indicated that the bioinspired biphasic OSHG group demonstrated superior antibacterial efficacy compared to the control (Fig. S26). Compared with PBS, the lipophilic drug thymol dissolved more readily in medical oil, enabling greater thymol release and contact with bacteria, thereby achieving an enhanced antibacterial effect.

### 3.5 Promotion of the healing of infected diabetic wounds

**Fig. 6:**
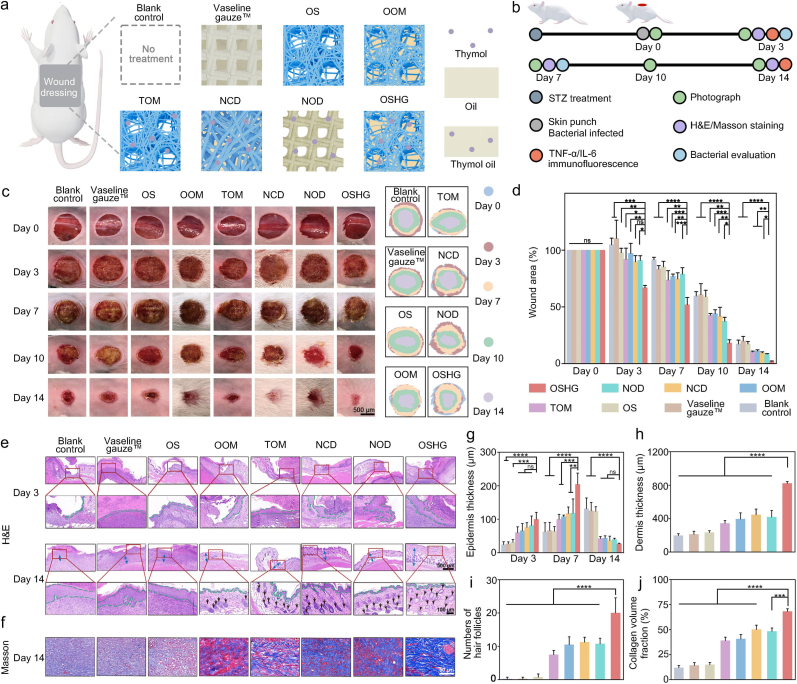
The effect of the bioinspired biphasic OSHG dressing on the healing of bacterial-infected diabetic wounds. Schematic illustration of (**a**) treatment schedule and (**b**) experimental process. (**c**) Photographs of wounds in different groups. (**d**) Statistical analysis of wound areas in different groups (n = 3). (**e**) H&E staining results on days 3 and 14 (green dotted line and blue arrow: epidermal layer; black arrow: hair follicle). (**f**) Masson staining images on day 14. (**g**) Epidermal thickness in different groups on days 3, 7, and 14 (n = 4). (**h**) Dermal thickness in different groups on day 14 (n = 4). (**i**) Number of hair follicles on day 14 (n = 4). (**j**) Collagen deposition on day 14 (n = 3). (NOD: The dressing with ongoing exudate transport capability. NCD: The dressing without exudate transport function. OOM: The dressing with a single-defense antibacterial activity. TOM: The dressing with a single-attack antibacterial activity. OSHG: The dressing with exudate-triggered gating transport and “defense-attack” antibacterial performance.)

To validate the pro-healing efficacy, a full-thickness wound model infected with *S. aureus* was established on the back of diabetic mice (Fig. 6a and b). The progress of wound healing was monitored on days 0, 3, 7, 10, and 14 (Fig. 6c). By day 14 (Fig. 6d), the bioinspired biphasic group achieved a wound healing rate of 98%, representing a 19% and 23% increase compared to the blank control group and the commercial Vaseline gauze™ group, respectively. Moreover, we compared the wound-healing efficacy of the bioinspired biphasic group with two commercial exudate management dressings: a hydrogel dressing that provides moisture and a foam dressing that absorbs exudate. We also evaluated the pro-healing effect of the NOD dressing with ongoing exudate transport capability and the NCD dressing without exudate transport function. The results demonstrated that our dressing achieved superior wound-healing outcomes compared with both two commercial dressings as well as the NOD and NCD dressings (Fig. 6c and d and Fig. S27). Finally, we compared our dressing with commercial silver-containing dressing, the OOM dressing with a single-defense antibacterial mechanism, and the TOM dressing with a single-attack antibacterial mechanism. The results showed that our dressing promoted significantly faster wound healing than all control groups (Fig. 6c and d and Fig. S28).

H&E staining was further used to assess re-epithelialization process. The results indicated that the damaged epidermis in mice of the bioinspired biphasic group was progressively repaired over time, and this repair occurred at a faster rate than in the other groups (Fig. 6e, Fig. S29). The bioinspired biphasic group also exhibited a higher level of angiogenesis and greater dermal thickness compared to the other groups (Fig. S30). Collagen deposition and follicular neogenesis served as critical histological hallmarks of successful dermal regeneration and restored biomechanical competence during tissue remodeling. As shown in Fig. 6f–j, both the collagen deposition and the number of newly formed hair follicles of the bioinspired biphasic group was the highest among all the groups. Overall, all these results demonstrated that the bioinspired biphasic dressing could promote re-epithelialization, angiogenesis, collagen deposition, and hair follicle formation, thereby accelerating the wound healing process.

**Fig. 7:**
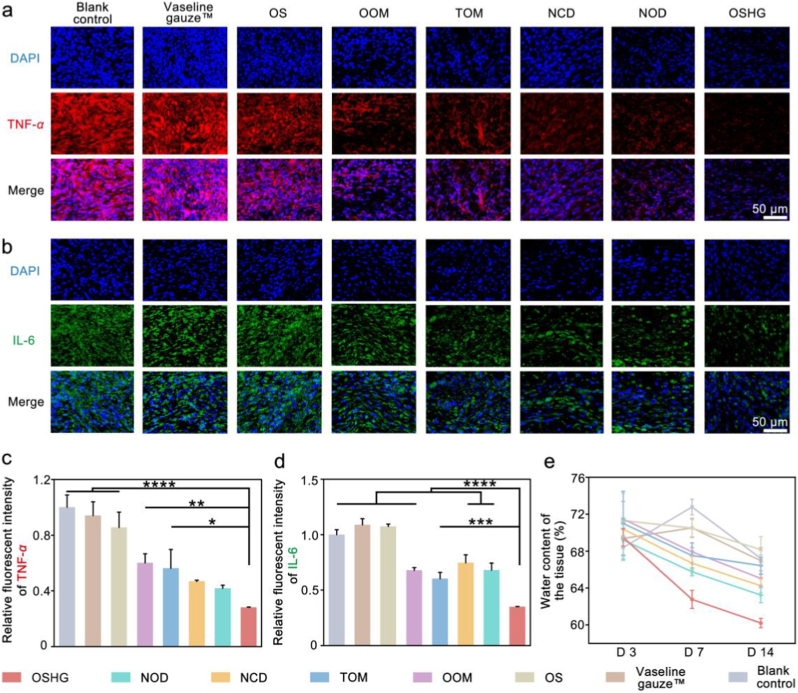
Immunofluorescence staining results and wound tissue water content after different treatments. Immunofluorescence staining results of (**a**) TNF-α (red) and (**b**) IL-6 (green) on day 3 (n = 3). Relative fluorescence intensity of (**c**) TNF-α and (**d**) IL-6 (n = 3). (**e**) Wound tissue water content results (n = 3).

Excessive exudate can lead to tissue edema and impair wound microcirculation [45]. Herein, the effect of exudate-triggered gating transport on wound healing was investigated. As shown in Fig. 7a and c, the bioinspired biphasic dressing group exhibited the lowest TNF-α fluorescence intensity, representing 39% and 31% reductions compared to the NOD and NCD groups, respectively. Similarly, the bioinspired biphasic dressing group demonstrated the lowest IL-6 fluorescence intensity (Fig. 7b and d). On day 14, the bioinspired biphasic dressing group maintained the lowest expression levels of both TNF-α and IL-6 (Figs. S31–S34). In the bioinspired biphasic dressing group, the infiltration of various inflammatory cells was also significantly reduced (Fig. S35). Subsequently, wound tissue water content was assessed, revealing that the bioinspired biphasic dressing group exhibited the lowest level (Fig. 7e). Collectively, these findings demonstrated that the bioinspired biphasic dressing effectively mitigated inflammation by efficiently removing wound exudate and reducing tissue edema, thereby establishing a moist and favorable microenvironment conducive to healing.

**Fig. 8:**
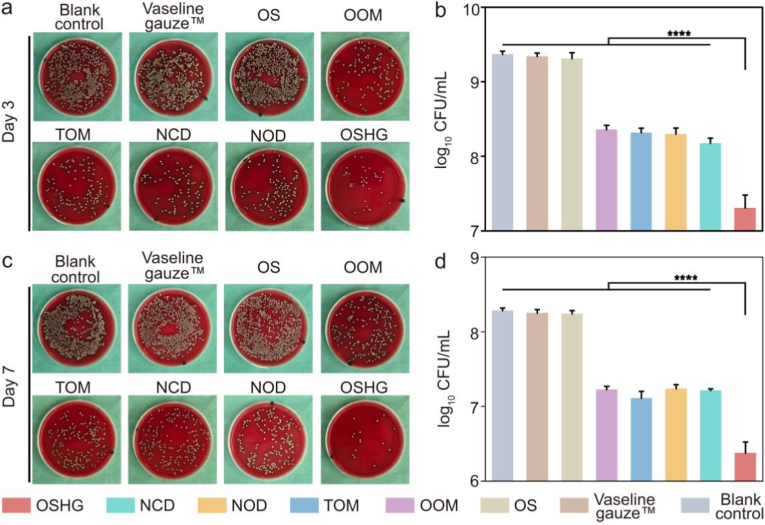
*In vivo* antibacterial performance. Representative images of *in vivo* bacterial colonies harvested from wounds on (**a**) day 3 and (**c**) day 7. Quantitative analysis of antibacterial efficacy for different groups on (**b**) day 3 and (**d**) day 7 (n = 4).

The antibacterial performance of the bioinspired biphasic dressing on wound healing was systematically evaluated. After 3 days of treatment, the antibacterial efficacy of the bioinspired biphasic group reached nearly 99%, significantly outperforming the OOM and TOM groups (Fig. 8a and b). On day 7, the bioinspired biphasic dressing continued to demonstrate superior antibacterial performance (Fig. 8c and d). Additionally, the bioinspired biphasic dressing demonstrated potent *in vivo* anti-biofilm capability (Fig. S36). The excellent antibacterial property was closely associated with the “defense-attack” synergistic antibacterial mechanism.

Owing to its multifunctional design, the bioinspired biphasic dressing also held potential for alleviating the hypoxic microenvironment. Briefly, the dressing could effectively and continuously remove excessive exudate, reduce edema in the wound and surrounding tissues, and thereby enhance local oxygen delivery efficiency [46]. Simultaneously, during exudate clearance, abundant pro-inflammatory factors were also removed, facilitating neovascularization and improving subsequent oxygen supply. Additionally, in infected wounds, bacterial growth, proliferation, and metabolism caused significant oxygen consumption. The antibacterial function of the dressing substantially reduced bacterial load and biofilm formation, alleviating the local hypoxic environment [47,48].

The bioinspired biphasic dressing was also proved to promote vascularization and re-epithelialization, with the mechanisms potentially attributed to the following aspects. First, active drainage of excessive exudate maintained a moist microenvironment for endothelial cell proliferation and reduced tissue edema, ensuring adequate oxygen and nutrient supply for angiogenesis [49]. Second, clearance of pro-inflammatory factors (e.g., TNF-α, IL-1β) alleviated persistent inflammation and accelerated transition to the proliferative phase [9]. Third, the antibacterial effect ensured unobstructed re-epithelialization. Collectively, this dressing promoted angiogenesis and re-epithelialization through moisture regulation, inflammation modulation, and antibacterial action.

Clinically, exuding infected wounds are often associated with excessive exudates production and bacterial colonization, both of which significantly impede the wound healing process. Moreover, chronic wounds require frequent dressing changes due to ongoing treatment needs, and the adhesion between dressings and wound bed further complicates the healing process by causing additional tissue damage during removal. Thus, the development of multifunctional dressings with self-adaptive liquid-gating transport, antibacterial activity, and anti-adhesion characteristics is highly urgent.

Till now, various dressings have been designed for wound exudate management. However, conventional commercial dressings present a moisture management paradox. Briefly, hydrogels maintain hydration but pose maceration risks [50], while foams absorb exudate effectively yet might desiccate the wound bed [14]. To achieve improved exudate control, dressings capable of directional exudate transport have been further developed. Conventional designs generally fall into two main categories: hydrophobic-hydrophilic Janus bilayer structures [9,51] or vertically aligned pore structures [52,53]. In these dressings, liquid transport is driven by the wettability gradient between hydrophobic and hydrophilic Janus layers or by the capillary force of vertically aligned pores. However, continuous drainage of exudate may lead to excessive wound dehydration, which is detrimental to wound healing.

To tackle the above challenges, a trilayer-structured gating transport dressing was recently designed, which consisted of a bottom hydrophobic nanofiber layer, a top hydrophilic gauze layer, and an intermediate thermo-responsive hydrophobic-hydrophilic switchable layer [15]. By applying external thermal stimulation, the intermediate layer was switched from hydrophobic to hydrophilic state to enable liquid transport, while when the thermal stimulus was removed, the intermediate layer was changed to the hydrophobic state again and liquid transport ceased. In summary, the liquid transport process of this multilayer dressing was controlled by passive liquid-gating and external heating, and for determining the switching timing of the heating source, manually removing the dressing was required to observe the wound exudate volume.

Distinct from the above structural designs, our study introduced a novel concave structure into the dressing, endowing it with active gating transport characteristics: when the exudate volume was high, the concave channels automatically opened to drain excess liquid, and when the exudate volume was low, the channels automatically closed to continuously maintain a moist wound microenvironment. The entire self-adaptive gating transport process occurred spontaneously, and required neither external heating sources nor frequent dressing removal. Furthermore, in comparison to single-channel sequential slow liquid transport in conventional flat oil-infused membranes, the unique concave array structure in our study also gave rise to a concave-mediated parallel channel flow model, which enhanced liquid transport speed by multi-channel parallel fast transport (Fig. S37a).

Concurrently, the newly designed concave array structure endowed the dressing with ultralow adhesion properties. Previously reported liquid-infused surfaces relied solely on internal lubricating oil to reduce tissue adhesion [54]. In contrast, our bioinspired biphasic dressing with concave array structure could reduce tissue adhesion not only through the lubricating effect of the internal medical oil, but also through the concave geometry that reduced contact area with the wound surface, thereby minimizing physical adhesion risk.

Our design also achieved new functional synergy through the combination of medical oil and thymol, which was different from the conventional single antibacterial function of thymol-loaded dressings and the conventional dual antimicrobial-loaded dressings without synergistic effects [55,56]. Briefly, thymol possessed broad-spectrum antibacterial activity, exerting offensive antibacterial action; while medical oil inhibited bacterial adhesion through its lubricating effect, thereby exerting defensive antibacterial action. Notably, the two active components also exerted a synergistic antibacterial effect, with the medical oil potentiating the activity of thymol (Fig. S37b).

Benefiting from the innovation of the concave structure design and the combination of medical oil and thymol, our bioinspired biphasic dressing exhibited unique self-adaptive liquid-gating transport, ultralow adhesion, and synergistic antibacterial properties, which was of significance for the treatment of exuding infected wounds. During dressing changes, our dressing exhibited minimal adhesion to the wound surface and could be easily removed, primarily owing to the lubricating effect of the medical oil and the reduced contact area resulting from the concave structure. Furthermore, benefiting from the self-adaptive liquid-gating transport and antibacterial properties, the dressing demonstrated excellent wound healing promotion.

We also consider the practical application and clinical translation of this bioinspired biphasic dressing. In clinical applications, postural changes of patients may induce oil phase migration in oil-infused dressings, representing a critical factor affecting dressing stability and therapeutic efficacy. In this study, the lipophilic PCL nanofiber membrane and inter-fiber capillary pores firmly retain the medical oil within the dressing, preventing oil phase migration during postural changes. Moreover, this dressing is fabricated via a one-step concave array nanofiber membrane preparation followed by simple impregnation with drug-loaded medical oil, rendering it readily amenable to scale-up production. In addition, the raw materials used in this study (medical-grade PCL, medical oil, and thymol) are cost-effective for practical applications.

Although the bioinspired biphasic design proposed in this study offers a novel approach for managing exuding infected wounds, certain limitations remain to be addressed. In future work, we aim to advance the capabilities of the dressing by incorporating functions such as exudate detection and on-demand therapeutic delivery. Specifically, we plan to integrate flexible electronic components into the dressing to enable real-time monitoring of key parameters of the exudates, including its chemical composition, pH, color, and volume, thereby facilitating precise assessment of wound status and healing progression. Additionally, we will investigate electrothermal or photothermal control systems to enable stimulus-responsive drug release tailored to the characteristics of the exudate, thus promoting more efficient wound closure. Furthermore, we intend to incorporate a broad range of therapeutic agents, such as anti-inflammatory, antioxidant, and pro-angiogenic pounds, into the dressing based on the specific exudate profile, allowing for targeted treatment that meets the diverse pharmacological demands of different wound healing stages.

## 4 Conclusions

In summary, the bioinspired biphasic dressing featuring concave array structure integrated with medical antibacterial oil was successfully prepared. Thanks to the dynamic liquid-solid interaction, the dressing exhibited unique self-adaptive liquid-gating transport behavior, enabling the removal of excessive liquid while retaining trace amounts, thereby dynamically regulating moisture balance. The medical antibacterial oil endowed the bioinspired biphasic dressing with synergistic antibacterial properties. In addition, the dressing exhibited excellent anti-adhesion properties due to the lubricating effect of the medical oil and the concave array structure, with peeling energy reduced to only 15% of that of commercial anti-adhesive dressing. The dressing could effectively promote the healing of infected diabetic wounds by maintaining optimal moisture level, alleviating inflammatory responses, and enhancing antibacterial efficacy. The bioinspired biphasic design strategy not only advances the frontiers of biomaterials development in fundamental research but also holds great promise for practical applications in chronic wound repair.

## CRediT authorship contribution statement

**Zizhao Chen:** Writing – original draft, Visualization, Methodology, Investigation. **Changming Wang:** Visualization, Methodology, Investigation. **Yixuan Li:** Visualization, Software, Methodology. **Chunsheng Guan:** Methodology, Formal analysis. **Mingzhang Li:** Methodology. **Hao Shen:** Supervision, Project administration. **Malcolm Xing:** Writing – review & editing, Supervision. **Botao Song:** Writing – review & editing, Supervision, Resources, Funding acquisition, Conceptualization. **Jiang Chang:** Writing – review & editing, Supervision, Conceptualization.

## Ethics approval and consent to participate

The animal procedures were reviewed by the Institutional Animal Care and Use Committee of Shanghai Sixth People's Hospital (Approval No.: DWLL2025-0929), ensuring compliance with national principles of animal protection, animal welfare, and ethics. Every effort was made to minimize animal suffering and reduce the number of animals used.

## Declaration of competing interest

Malcolm Xing is an editorial board member for Bioactive Materials and was not involved in the editorial review or the decision to publish this article. All authors declare that there are no competing interests.

## References

[1] Zhang Z., Li W., Liu Y., Yang Z., Ma L., Zhang H., Wang E., Wu C., Huan Z., Guo F., Chang J. (2021). Design of a biofluid-absorbing bioactive sandwich-structured Zn-Si bioceramic composite wound dressing for hair follicle regeneration and skin burn wound healing. Bioact. Mater..

[2] Sen C.K. (2021). Human wound and its burden: updated 2020 compendium of estimates. Adv. Wound Care.

[3] Harding K., Carville K., Chadwick P., Moore Z., Nicodème M., Percival S., Romanelli M., Schultz G., Tariq G. (2019).

[4] Wang X., Mu R., Zhou Y., Li J., Ma Q., Wang J., Ma Y., Sheng W., Hu X., Zhou F., Li B. (2025). Poly(ectoine)-based adhesive hydrogel with antimicrobial and anti-inflammatory properties for wound treatment. Adv. Funct. Mater..

[5] Falanga V., Isseroff R.R., Soulika A.M., Romanelli M., Margolis D., Kapp S., Granick M., Harding K. (2022). Chronic wounds. Nat. Rev. Dis. Primers.

[6] Li H., Wen H., Zhang H., Cao X., Li L., Hu X., Zhang Y., Shen X., Shubhra Q.T.H., Yang H., Cai X. (2025). A multifunctional dihydromyricetin-loaded hydrogel for the sequential modulation of diabetic wound healing and glycemic control. Burns & Trauma.

[7] Chen J., Mu Z., Chen D., Huang C., Jin T., Li L., Zeng Y., Zhou Q., Zhang Y., Mao H., Deng H., Shen X., Yang H., Cai X. (2023). H_2_S-releasing versatile hydrogel dressing with potent antimicrobial, anti-inflammatory, epithelialization and angiogenic capabilities for diabetic wound healing. Chem. Eng. J..

[8] Yang C., Liu G., Chen J., Zeng B., Shen T., Qiu D., Huang C., Lin L., Chen D., Chen J., Mu Z., Deng H., Cai X. (2022). Chitosan and polyhexamethylene guanidine dual-functionalized cotton gauze as a versatile bandage for the management of chronic wounds. Carbohydr. Polym..

[9] Xu Z., Fan J., Tian W., Ji X., Cui Y., Nan Q., Sun F., Zhang J. (2024). Cellulose-based pH-responsive janus dressing with unidirectional moisture drainage for exudate management and diabetic wounds healing. Adv. Funct. Mater..

[10] Shi L., Liu X., Wang W., Jiang L., Wang S. (2019). A self-pumping dressing for draining excessive biofluid around wounds. Adv. Mater..

[11] Jin X., Wu Y., Chen Z., Chen Z., Zhou F., Jin Q., Wu S., Feng Y., Ma J., Guo X., Chang J., Yang C., Song B. (2025). Bioinspired nanofiber dressings with counter-transport of exudate and drug for treating heavily exuding wounds. Biomaterials.

[12] Winter G.D. (1962). Formation of the scab and the rate of epithelization of superficial wounds in the skin of the young domestic pig. Nature.

[13] Winter G.D. (1963). Effect of air exposure and occlusion on experimental human skin wounds. Nature.

[14] Dyson M., Young S., Pendle C.L., Webster D.F., Lang S.M. (1988). Comparison of the effects of moist and dry conditions on dermal repair. J. Invest. Dermatol..

[15] Qiu Z., Gao Y., Qi D., Wu M., Mao Z., Wu J. (2024). Thermo-responsive trilayered fibrous dressing with liquid gate for dynamical exudate regulation and wound moisture balance. Adv. Funct. Mater..

[16] Chen S., Li A., Wang Y., Zhang Y., Liu X., Ye Z., Gao S., Xu H., Deng L., Dong A., Zhang J. (2023). Janus polyurethane sponge as an antibiofouling, antibacterial, and exudate-managing dressing for accelerated wound healing. Acta Biomater..

[17] Li L., Zhao Q., Feng F., Li C., Wang T., Chen W., Ren X., Huang Y., Li J. (2023). Hydro electroactive janus micro/nano fiber dressing with exudate management ability for wound healing. Adv. Mater. Technol..

[18] Huang X., Zheng L., Zhou Y., Hu S., Ning W., Li S., Lin Z., Huang S. (2024). Controllable adaptive molybdate-oligosaccharide nanoparticles regulate M2 macrophage mitochondrial function and promote angiogenesis via PI3K/HIF-1α/VEGF pathway to accelerate diabetic wound healing. Adv. Healthcare Mater..

[19] Simões D., Miguel S.P., Ribeiro M.P., Coutinho P., Mendonça A.G., Correia I.J. (2018). Recent advances on antimicrobial wound dressing: a review. Eur. J. Pharm. Biopharm..

[20] Li L., Chen D., Chen J., Yang C., Zeng Y., Jin T., Zhang Y., Sun X., Mao H., Mu Z., Shen X., Ruan Z., Cai X. (2023). Gelatin and catechol-modified quaternary chitosan cotton dressings with rapid hemostasis and high-efficiency antimicrobial capacity to manage severe bleeding wounds. Mater. Des..

[21] Epstein A.K., Wong T.S., Belisle R.A., Boggs E.M., Aizenberg J. (2012). Liquid-infused structured surfaces with exceptional anti-biofouling performance. Proc. Natl. Acad. Sci. USA.

[22] Costerton J.W., Stewart P.S., Greenberg E.P. (1999). Bacterial biofilms: a common cause of persistent infections. Science.

[23] Qiu D., Zheng C., Zeng Y., Wu L., Huang C., Ran Y., Ding Y., Shi J., Cai X., Pan Y. (2023). Enzymolysis and photothermal-mediated synergistic antimicrobial nanoplatform with programmed EPS degradation and biofilm penetration capabilities for eradication of biofilm wound infections. Chem. Eng. J..

[24] Xiu W., Wan L., Yang K., Li X., Yuwen L., Dong H., Mou Y., Yang D., Wang L. (2022). Potentiating hypoxic microenvironment for antibiotic activation by photodynamic therapy to combat bacterial biofilm infections. Nat. Commun..

[25] Jarad N.A., Rachwalski K., Bayat F., Khan S., Shakeri A., Maclachlan R., Villegas M., Brown E.D., Hosseinidoust Z., Didar T.F., Soleymani L. (2023). A bifunctional spray coating reduces contamination on surfaces by repelling and killing pathogens. ACS Appl. Mater. Interfaces.

[26] Li J., Kleintschek T., Rieder A., Cheng Y., Baumbach T., Obst U., Schwartz T., Levkin P. (2013). Hydrophobic liquid-infused porous polymer surfaces for antibacterial applications. ACS Appl. Mater. Interfaces.

[27] Chen J., Zhao X., Qiao L., Huang Y., Yang Y., Chu D., Guo B. (2024). Multifunctional on-demand removability hydrogel dressing based on in situ formed AgNPs, silk microfibers and hydrazide hyaluronic acid for burn wound healing. Adv. Healthcare Mater..

[28] Jiang Y., Wu Y., Wang T., Chen X., Zhou M., Ma C., Xi X., Zhang Y., Chen T., Shaw C., Wang L. (2020). Brevinin-1GHd: a novel *Hylarana guentheri* skin secretion-derived Brevinin-1 type peptide with antimicrobial and anticancer therapeutic potential. Biosci. Rep..

[29] Xiao W., Wan X., Shi L., Ye M., Zhang Y., Wang S. (2024). A viscous-biofluid self-pumping organohydrogel dressing to accelerate diabetic wound healing. Adv. Mater..

[30] McColl D., Cartlidge B., Connolly P. (2007). Real-time monitoring of moisture levels in wound dressings *in vitro*: an experimental study. Int. J. Surg..

[31] Villegas M., Zhang Y., Jarad N.A., Soleymani L., Didar T.F. (2019). Liquid-infused surfaces: a review of theory, design, and applications. ACS Nano.

[32] Keiser A., Keiser L., Clanet C., Quéré D. (2017). Drop friction on liquid-infused materials. Soft Matter.

[33] Yao X., Hu Y., Grinthal A., Wong T.S., Mahadevan L., Aizenberg J. (2013). Adaptive fluid-infused porous films with tunable transparency and wettability. Nat. Mater..

[34] Jia Y.W., Zhao X., Fu T., Li D.F., Guo Y., Wang X.L., Wang Y.Z. (2020). Synergy effect between quaternary phosphonium ionic liquid and ammonium polyphosphate toward flame retardant PLA with improved toughness. Compos Part B-Eng..

[35] Bazyar H., Javadpour S., Lammertink R.G.H. (2016). On the gating mechanism of slippery liquid infused porous membranes. Adv. Mater. Interfac..

[36] Hou X., Hu Y., Grinthal A., Khan M., Aizenberg J. (2015). Liquid-based gating mechanism with tunable multiphase selectivity and antifouling behaviour. Nature.

[37] Wong T., Kang S., Tang S., Smythe E., Hatton B., Grinthal A., Aizenberg J. (2011). Bioinspired self-repairing slippery surfaces with pressure-stable omniphobicity. Nature.

[38] Song C., Rutledge G.C. (2022). Electrospun liquid-infused membranes for emulsified oil/water separation. Langmuir.

[39] Hou X., Li J., Tesler A.B., Yao Y., Wang M., Min L., Sheng Z., Aizenberg J. (2018). Dynamic air/liquid pockets for guiding microscale flow. Nat. Commun..

[40] Okan D., Woo K., Ayello E.A., Sibbald G. (2007). The role of moisture balance in wound healing. Adv. Skin Wound Care.

[41] Baier R.E. (2006). Surface behaviour of biomaterials: the theta surface for biocompatibility. J. Mater. Sci. Mater. Med..

[42] Brichacek M., Ning C., Gawaziuk J.P., Liu S., Logsetty S. (2017). *In vitro* measurements of burn dressing adherence and the effect of interventions on reducing adherence. Burns.

[43] Andrews E.H., Kamyab I. (1986). Adhesion of surgical dressings to wounds: a new *in vitro* model. Clin. Mater..

[44] Ning C., Logsetty S., Ghughare S., Liu S. (2014). Effect of hydrogel grafting, water and surfactant wetting on the adherence of pet wound dressings. Burns.

[45] Spear M. (2012). Wound exudate-the good, the bad, and the ugly. Plast. Surg. Nurs..

[46] Gong X., Wang F., Yang J., Du H., Jiang M., Tan M., Chen G., Chen Z. (2024). Engineered composite dressing with exudate management capabilities for the process of entire wound healing. Mater. Today Commun..

[47] Thaarup I.C., Iversen A.K.S., Lichtenberg M., Bjarnsholt T., Jakobsen T.H. (2022). Biofilm survival strategies in chronic wounds. Microorganisms.

[48] James G., Zhao A., Usui M., Underwood R., Nguyen H., Beyenal H., Pulcini E., Hunt A., Bernstein H., Fleckman P., Olerud J., Williamson K., Franklin M., Stewart P. (2016). Microsensor and transcriptomic signatures of oxygen depletion in biofilms associated with chronic wounds. Wound Repair Regen..

[49] Sha Z., Li J., Song Y., Li H., Liu H., Fan J., Li X., Fei X., Zhu M. (2025). Self-pumping, pH-responsive Janus fibrous dressing for enhanced immunomodulation and accelerated diabetic wound healing. Nano Today.

[50] Jones V., Grey J.E., Harding K.G. (2006). Wound dressings. Br. Med. J..

[51] Feng F., Zhao Z., Li J., Huang Y., Chen W. (2024). Multifunctional dressings for wound exudate management. Prog. Mater. Sci..

[52] Li Z., Wang L., Zheng Z., Yin K., Du Z., Liu J., Zhang X., Wang X., Tan Y., Liao L., He W., Zhang C., Lin Q. (2025). Directional pore size-mediated blood hydrodynamics govern hemostasis in cellulose sponges. Small.

[53] Guan C., Li M., Zhou X., Zhou Y., You Y., Shen H., Song B. (2025). 1D nanofiber-2D nanosheet assembled 3D bioinspired dressings for treating exuding infected wounds. ACS Appl. Mater. Interfaces.

[54] Yuan S., Sun X., Yan S., Luan S., Song L., Yin J. (2022). Slippery 3-dimensional porous bioabsorbable membranes with anti-adhesion and bactericidal properties as substitute for vaseline gauze. Colloids Surf., B.

[55] Yue Y., Gong X., Jiao W., Li Y., Yin X., Si Y., Yu J., Ding B. (2021). In-situ electrospinning of thymol-loaded polyurethane fibrous membranes for waterproof, breathable, and antibacterial wound dressing application. J. Colloid Interface Sci..

[56] AlSalem H., Bukhari A. (2023). Biodegradable wound dressing-based collagen/hyaluronic acid loaded antibacterial agents for wound healing application. Int. J. Biol. Macromol..

