# Supplementary material for: Bioinspired “liquid-solid” biphasic dressing with exudate-gating transport, defense-attack antibacterial activity, and anti-adhesion property for exuding infected wound therapy
Source: Bioact Mater. 2026 Apr 6;63:188–205. doi: 10.1016/j.bioactmat.2026.04.001 (PMC13090611; doi:10.1016/j.bioactmat.2026.04.001)
Supplement: Multimedia component 1 [file mmc1.docx]

**Supplementary Information contains:**

Supplementary Figures S1-S37

Supplementary Tables S1-S2

Supplementary Movies S1-S6

Supplementary References

1. **Supplementary Figures**


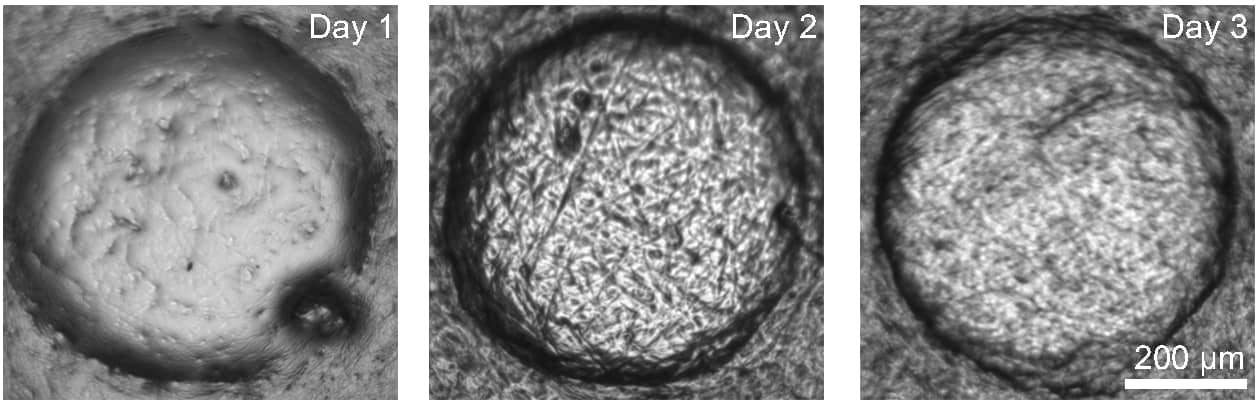


**Fig. S1** The image of the concave structure after different days immersion in PBS.


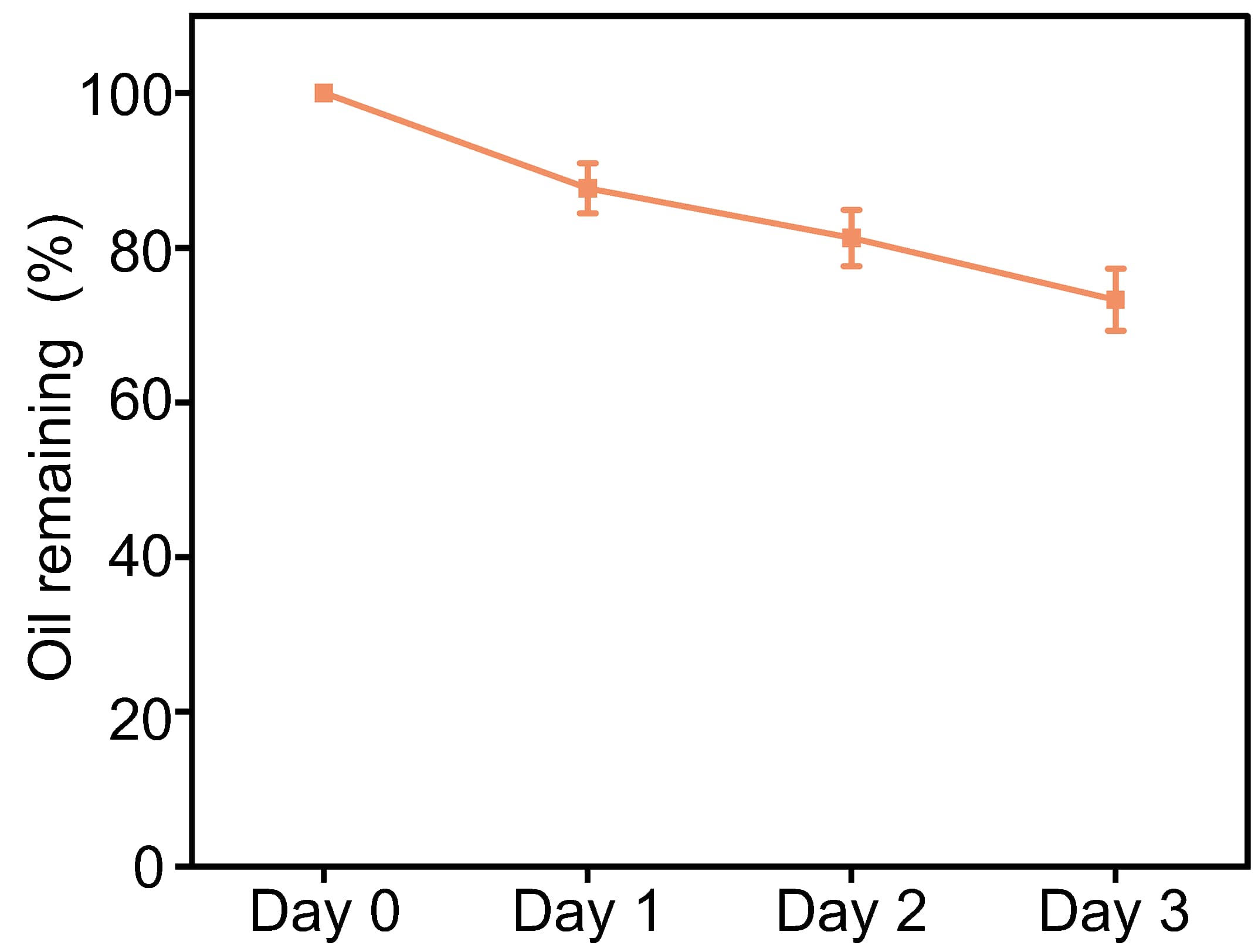


**Fig. S2** Oil remaining of the bioinspired biphasic dressing after 3 days of immersion in PBS.


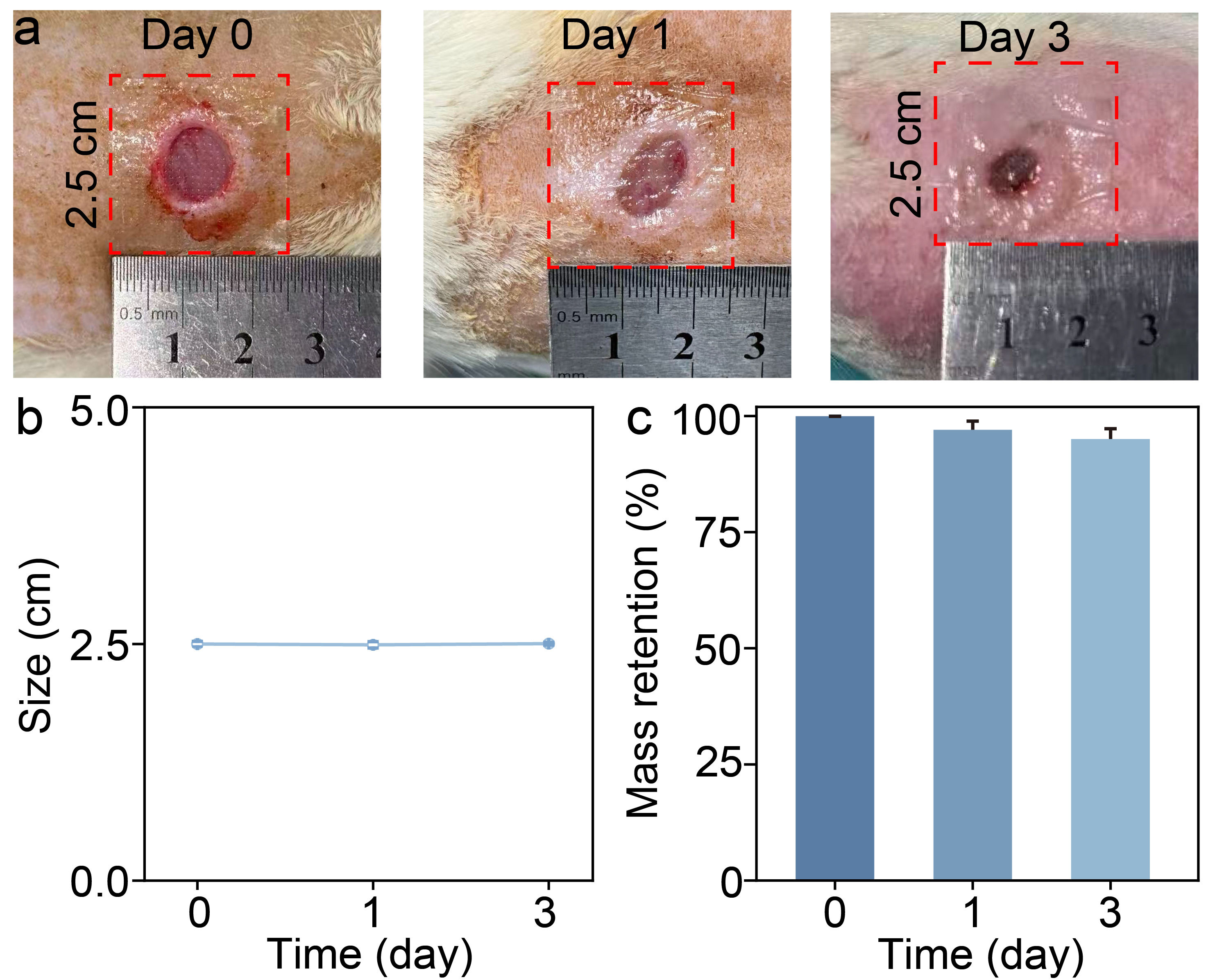


**Fig. S3** (a) Appearance of the bioinspired biphasic dressing on rat wound at different time points. (b) The size change of the dressing at different time points. (c) The mass of the dressing at different time points.


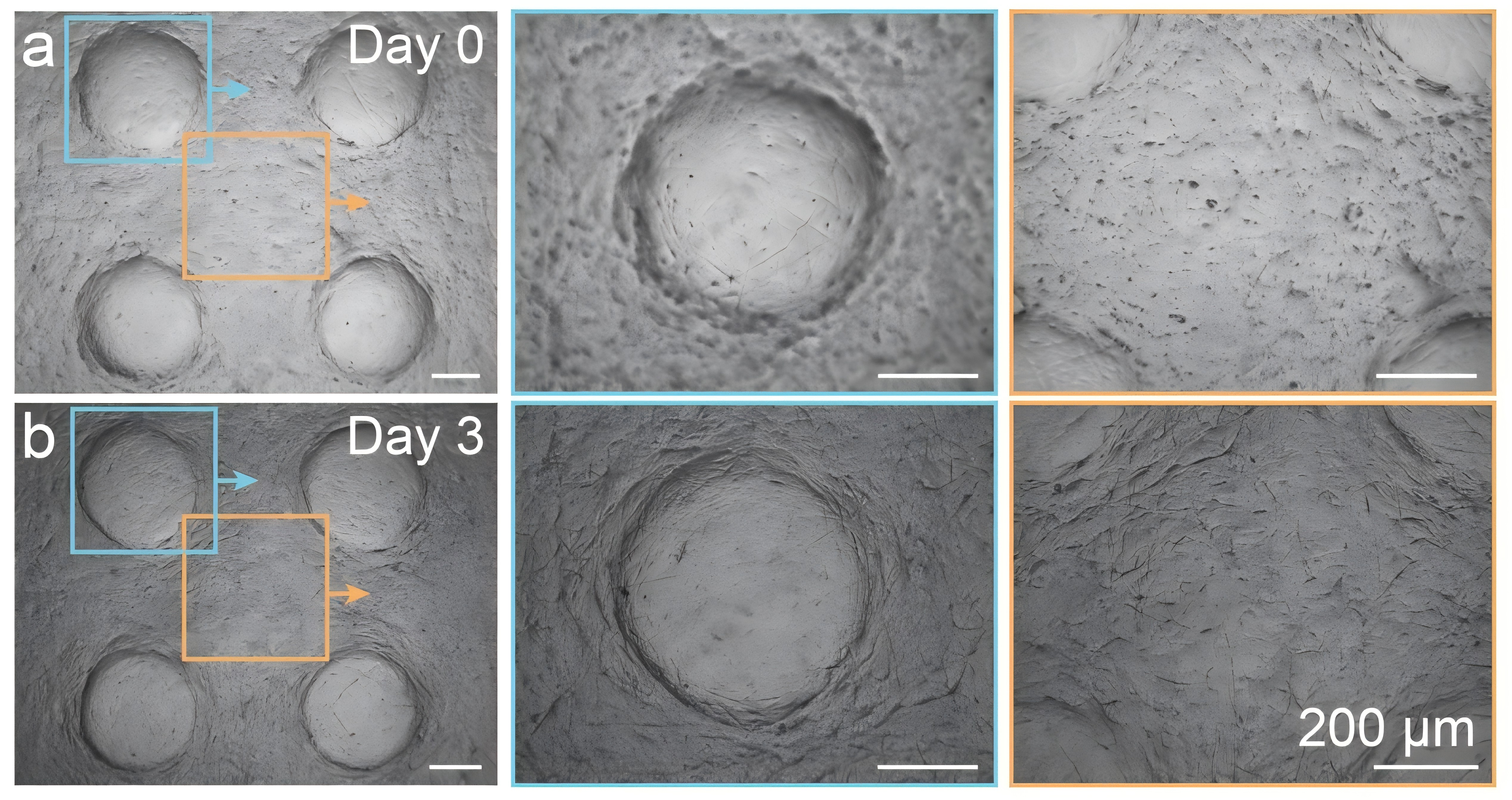


**Fig. S4** Oil retention status (a) before and (b) after 3 days of application on rat wound.


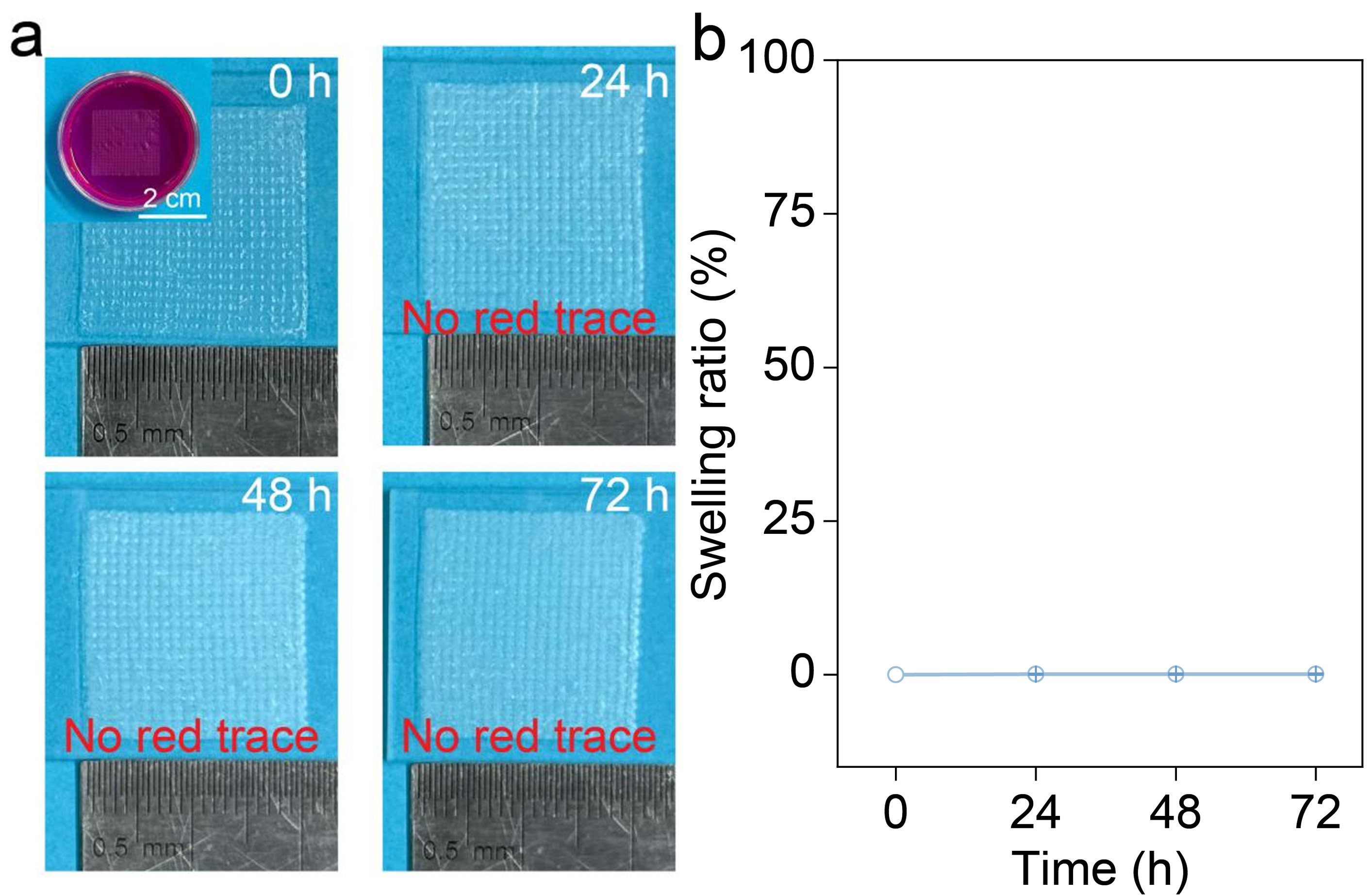


**Fig. S5** (a) The bioinspired biphasic dressing prevented absorption of the red rhodamine B-stained PBS. (The inset image showed the dressing fully immersed in the red solution.) (b) Swelling ratio of the dressing at different time points.


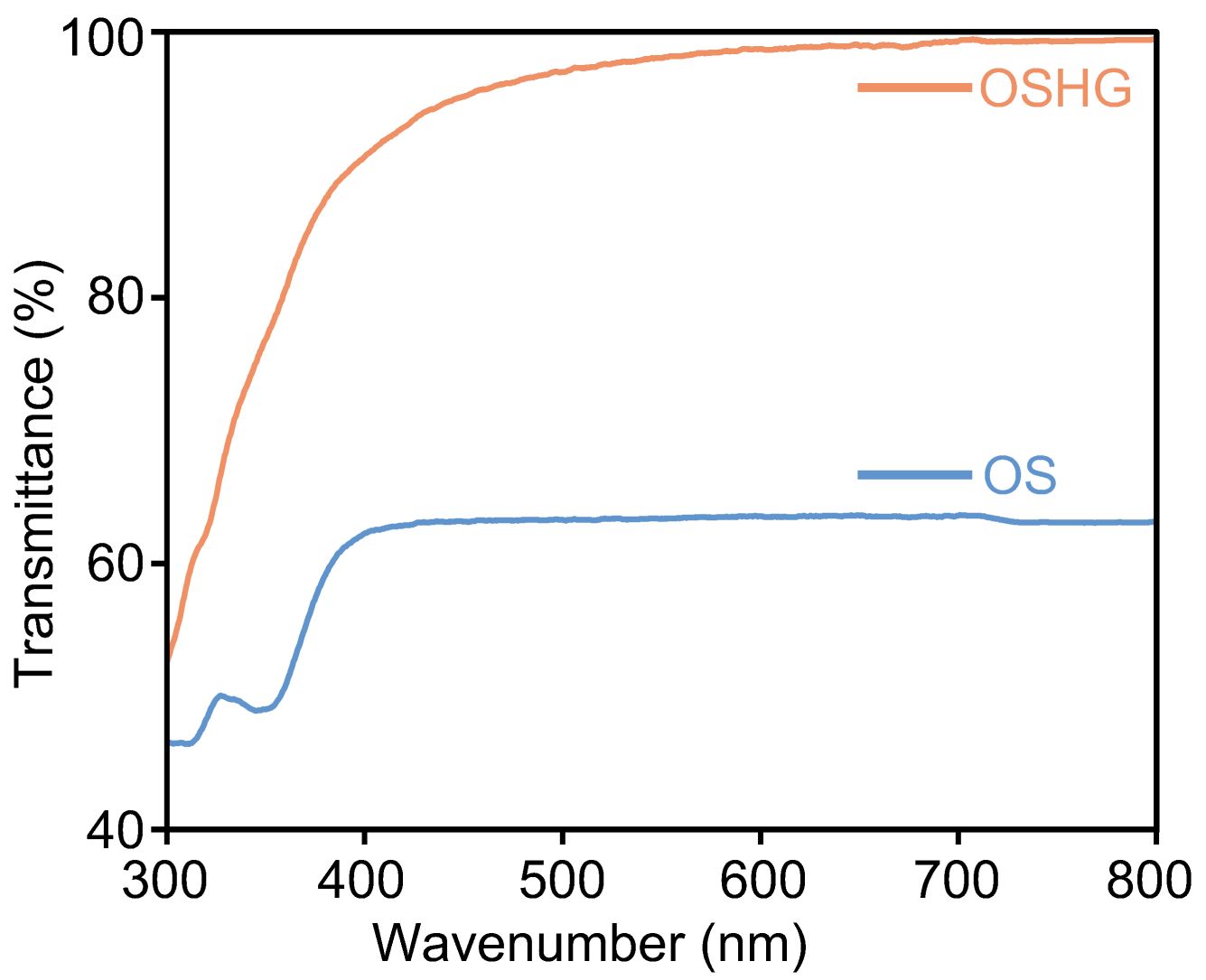


**Fig. S6** The transmittance of the bioinspired OS membrane and the bioinspired biphasic dressing.


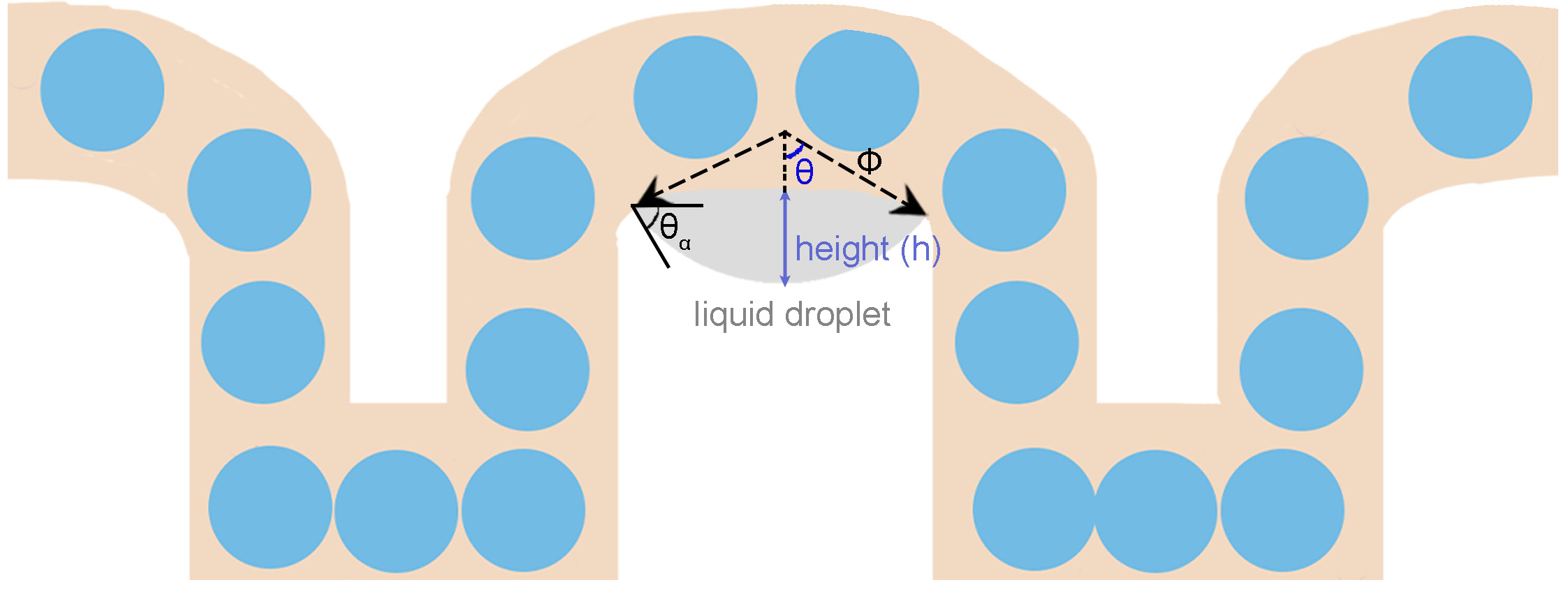


Fig. S7 Interaction of a liquid droplet with the bioinspired biphasic dressing.

Derived from geometric relationships in the spherical cap shaped droplet on the surface of the bioinspired biphasic dressing:

$\text{Φ = }\frac{\text{h}}{\text{1- cosθ}}\text{ }$ (1)

where Φ was the sphere diameter of the liquid droplet, h was the height of the liquid droplet, θ was the cone angle between the central axis and the droplet boundary to the sphere’s center.

Derived from the volume formula:

$\text{V = }\int_{\text{Φ-h}}^{\text{Φ}} \text{dV}\text{ = }\int_{\text{Φ-h}}^{\text{Φ}} \text{π}\text{(}\text{Φ}^{\text{2}}\text{ -}\text{z}^{\text{2}}\text{)}\text{dz}\text{ = π}\text{(}\frac{\text{1}}{\text{1-cosθ}}\text{ }\text{-}\frac{\text{1}}{\text{3}}\text{)}\text{ h}^{\text{3}}$ (2)

Formula 3 could be derived:

$\text{h}\text{ = (}\frac{\text{2 + cosθ}}{\text{3π(1-cosθ)}}\text{)}^{\frac{\text{1}}{\text{3}}}\text{V}^{\frac{\text{1}}{\text{3}}}$ (3)


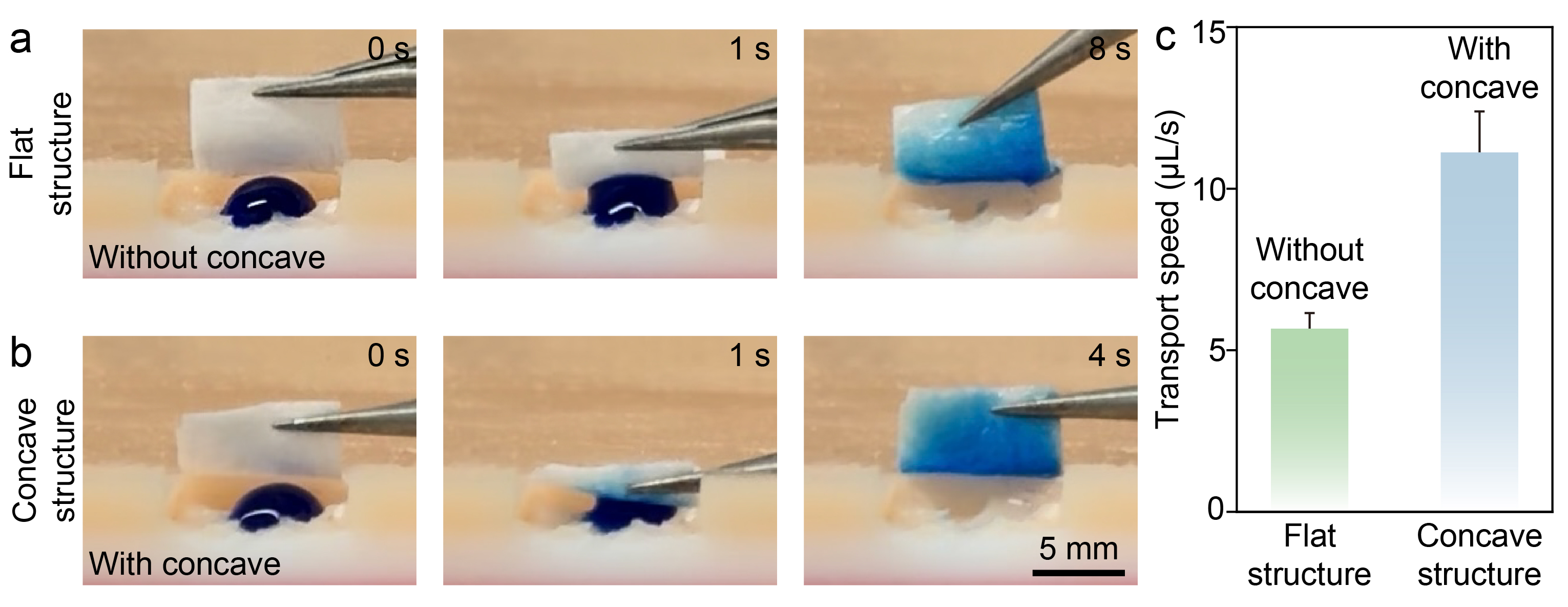


**Fig. S8** (a) Liquid transport behavior of the flat oil-infused dressing without concave structure. (b) Liquid transport behavior of the oil-infused dressing with concave structure. (c) Comparison of liquid transport speed between oil-infused dressings with and without concave structure.

**
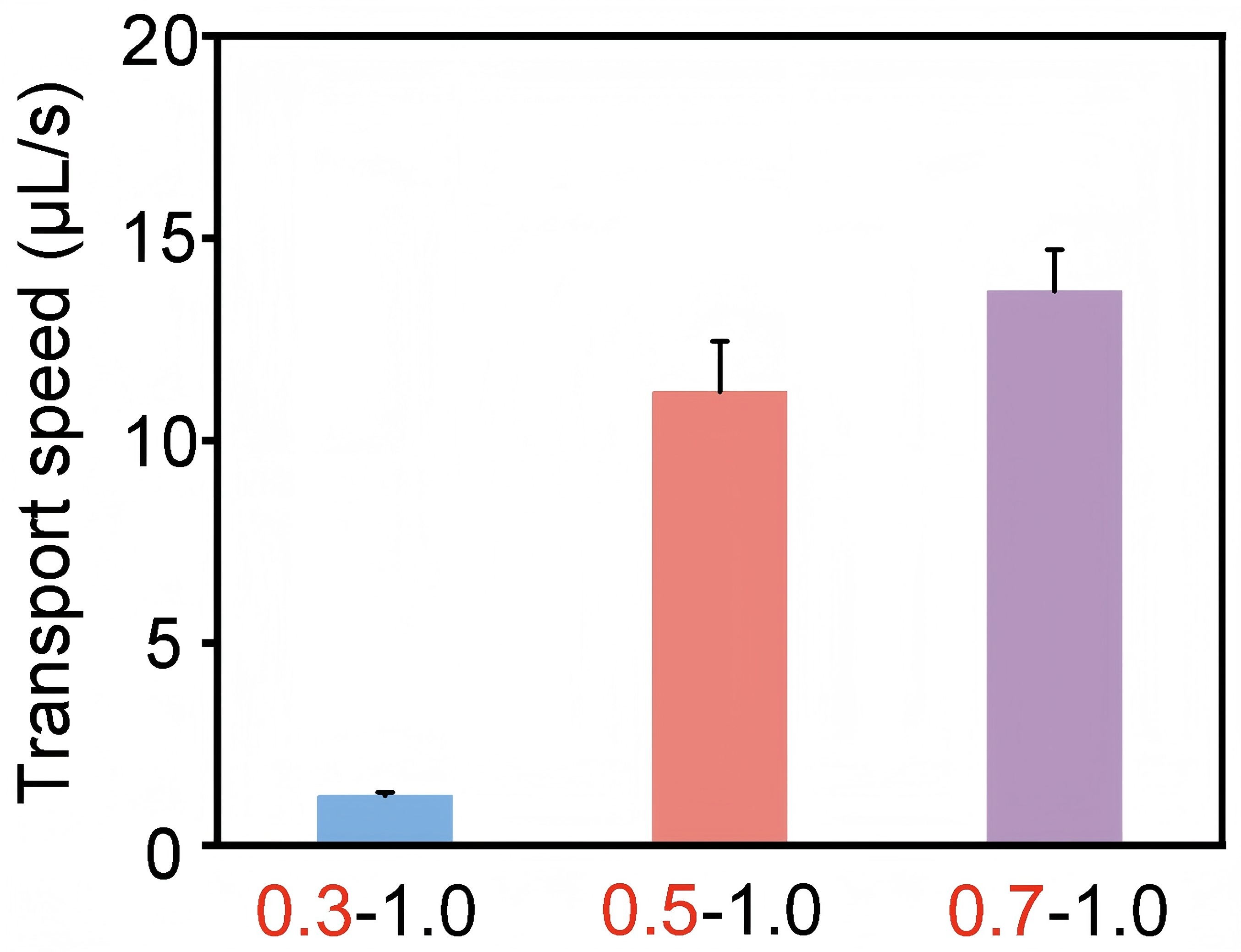
**

**Fig. S9** Liquid transport speeds of the bioinspired biphasic dressings with different concave diameters.


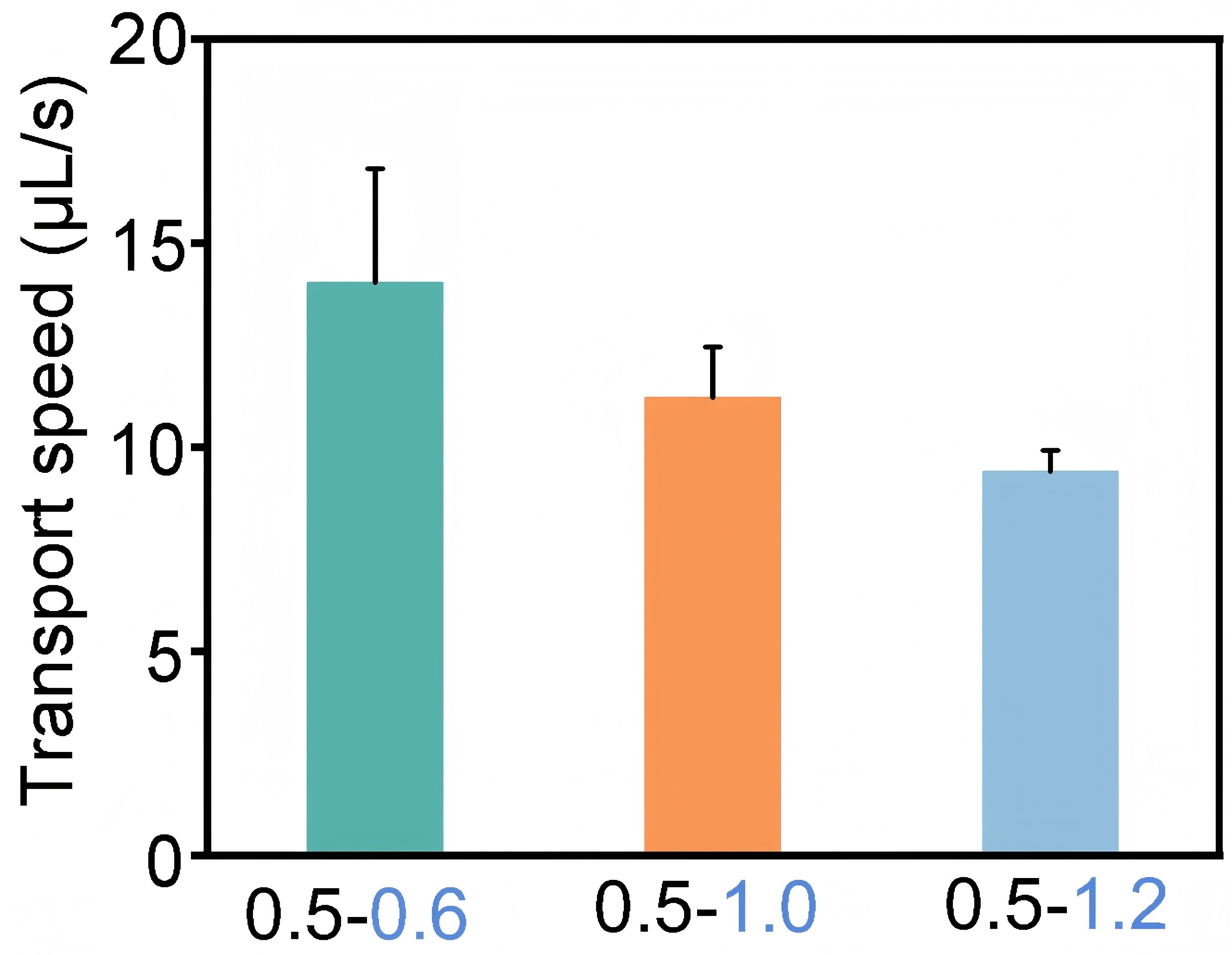


**Fig. S10** Liquid transport speeds of the bioinspired biphasic dressings with different concave spacings.


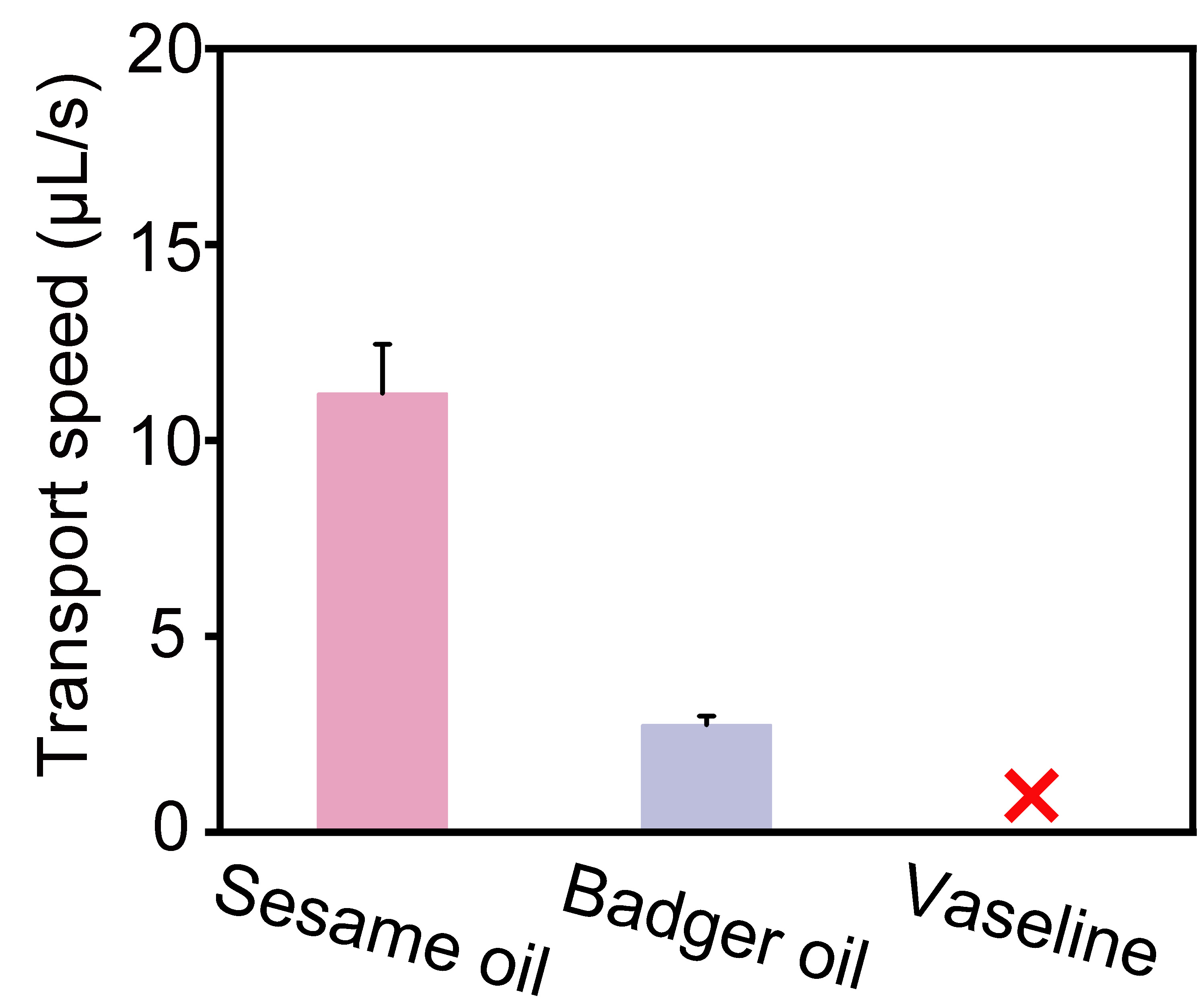


**Fig. S11** Liquid transport speeds of the bioinspired biphasic dressings loading with different medical oils.


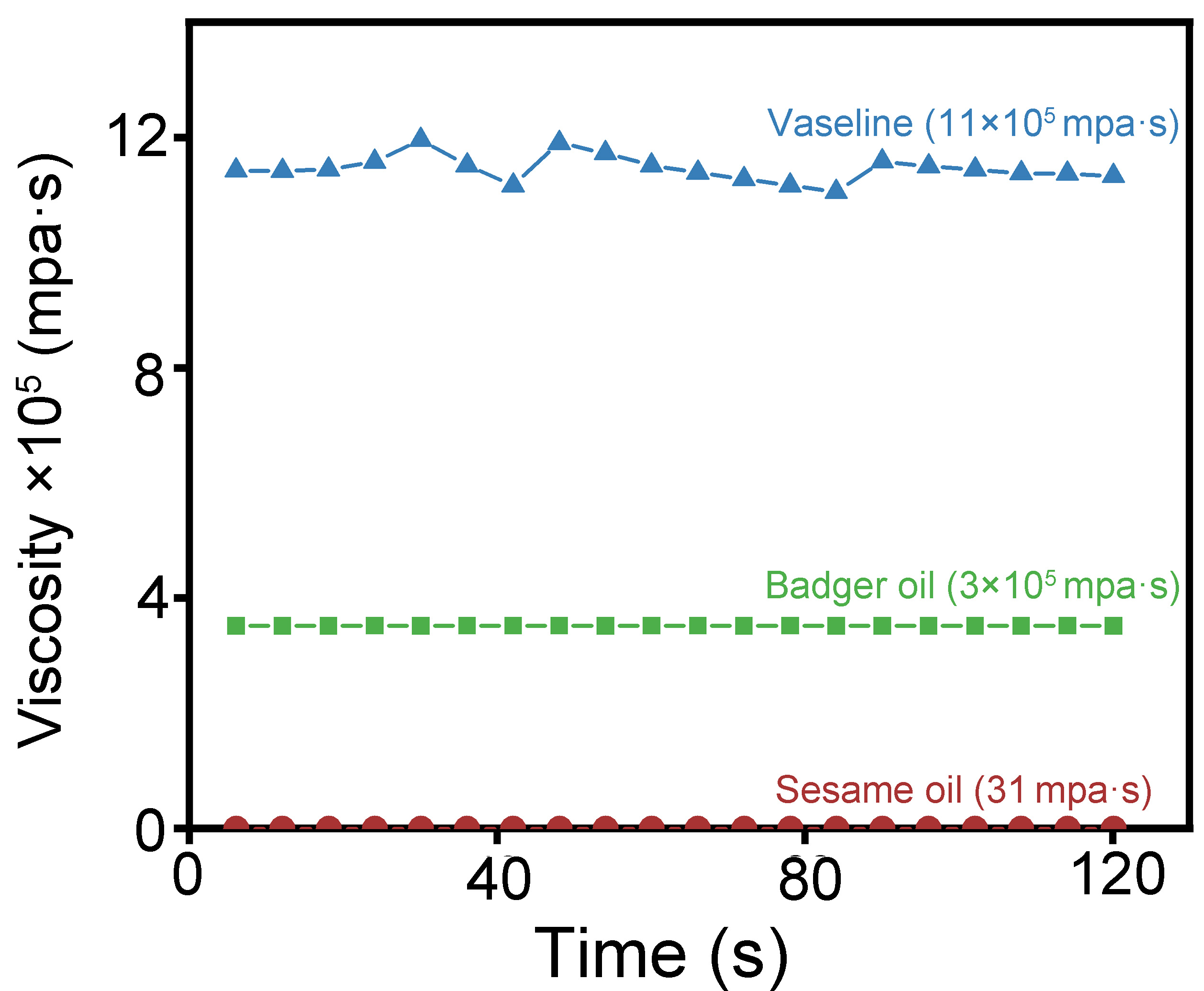


**Fig. S12** The viscosity of different medical oils.


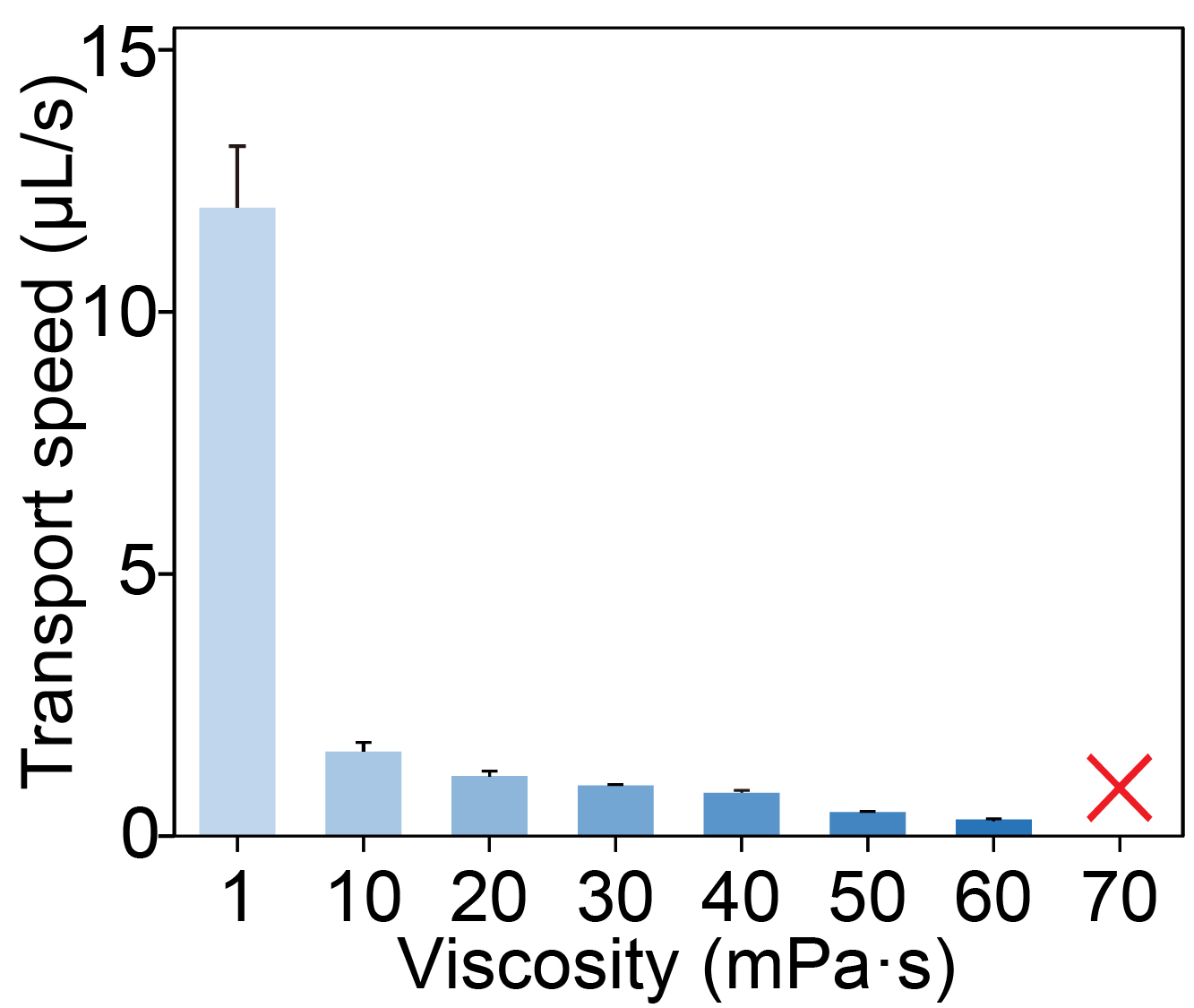


**Fig. S13** Liquid transport speed of the dressing for liquids with different viscosities.


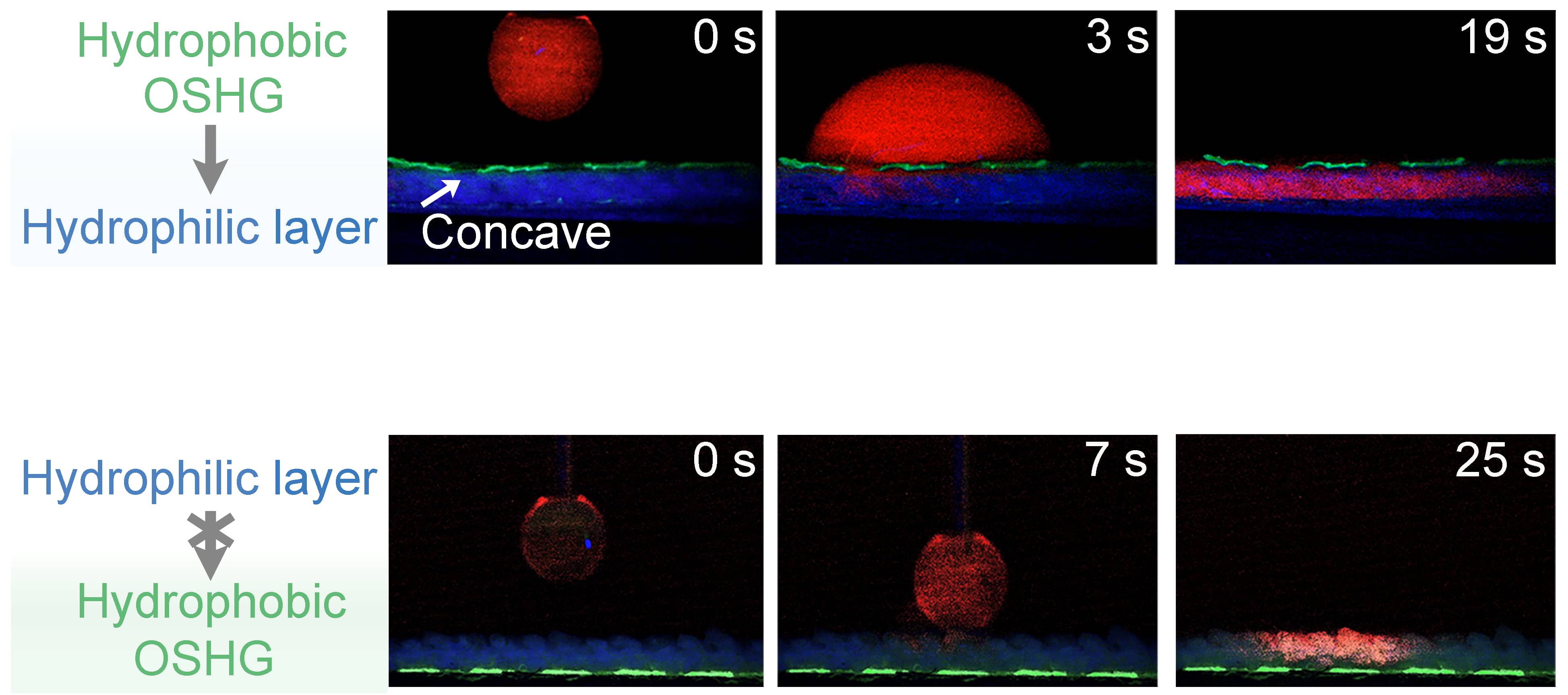


**Fig. S14** Pathway of liquid droplet transported from the OSHG layer.


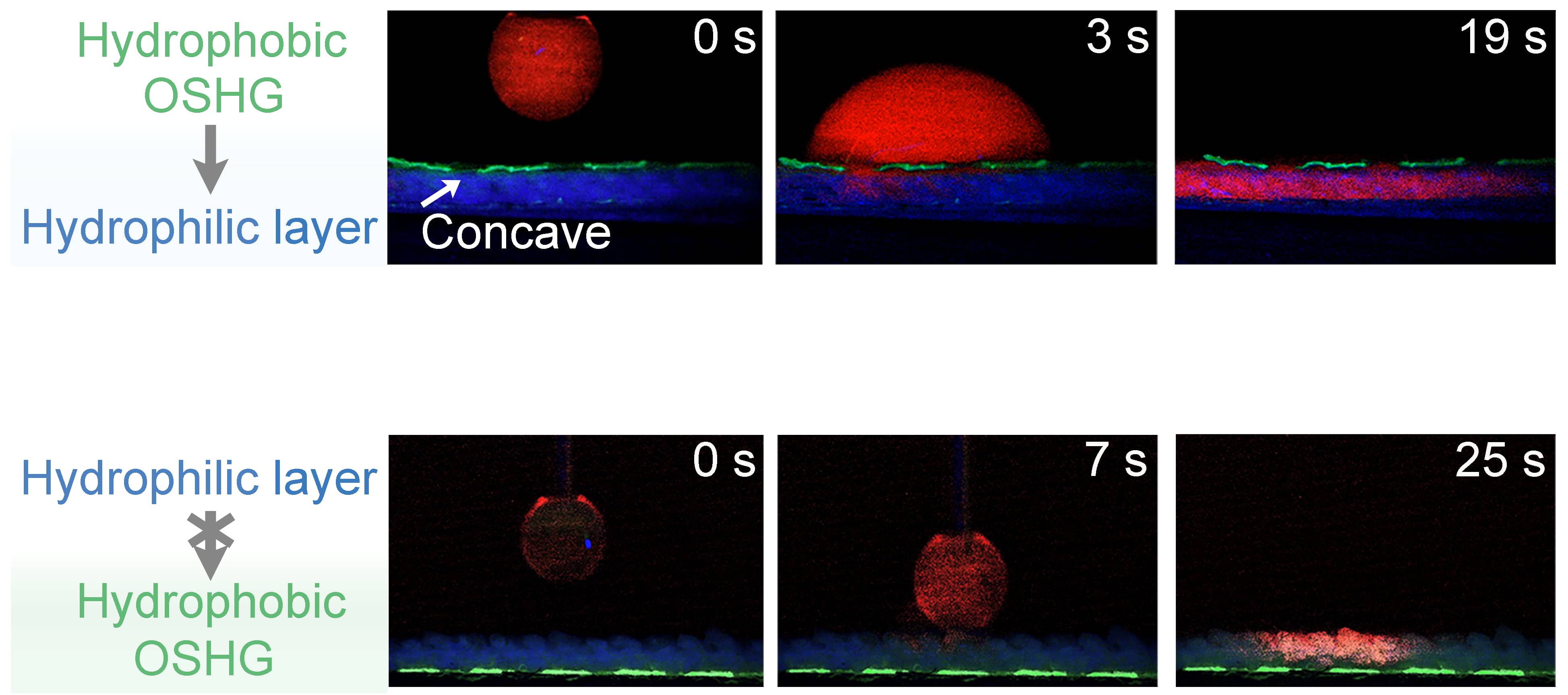


**Fig. S15** Pathway of liquid droplet transported from the hydrophilic layer.


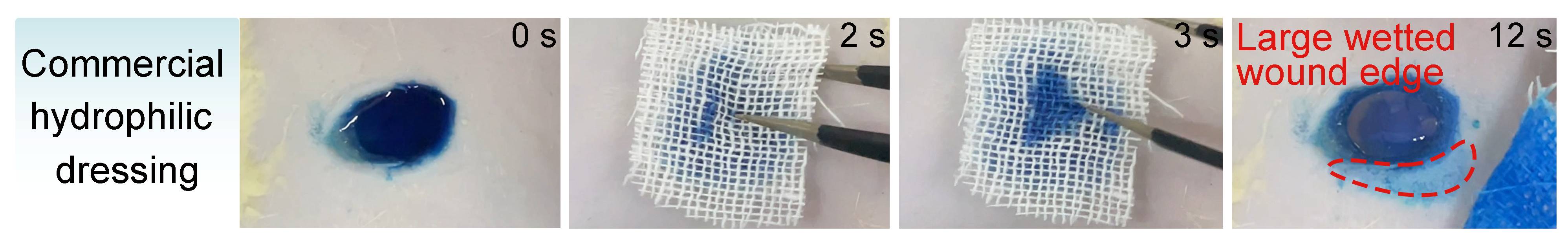


**Fig. S16** The blue imprint around the wound after the liquid transport by the gauze.


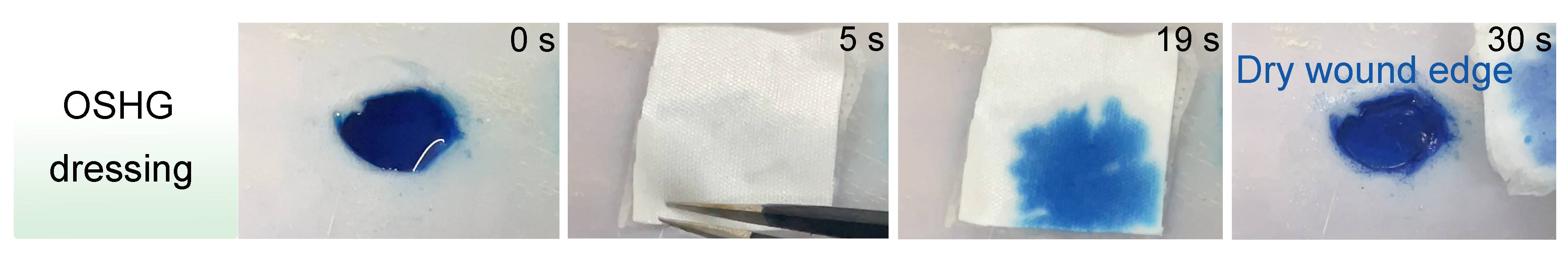


**Fig. S17** No blue imprint around the wound after the liquid transport by the bioinspired biphasic dressing.


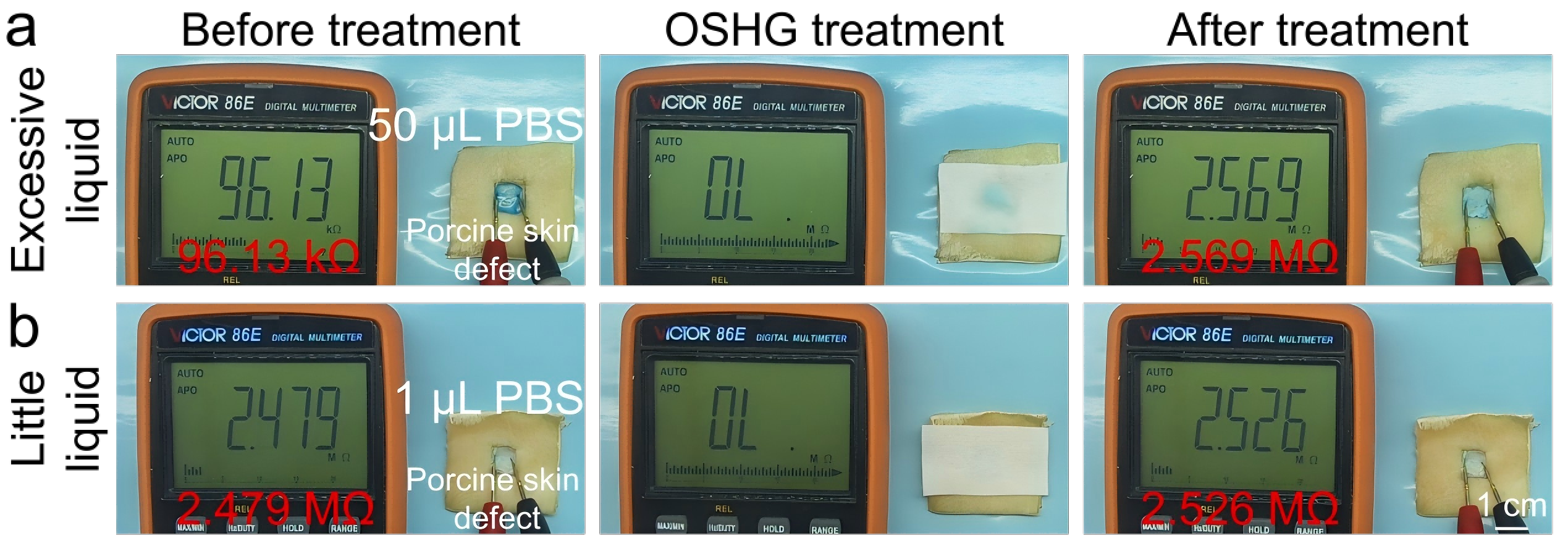


**Fig. S18** The changes in resistance before and after application of OSHG dressing on wounds with (a) high liquid volume and (b) low liquid volume.


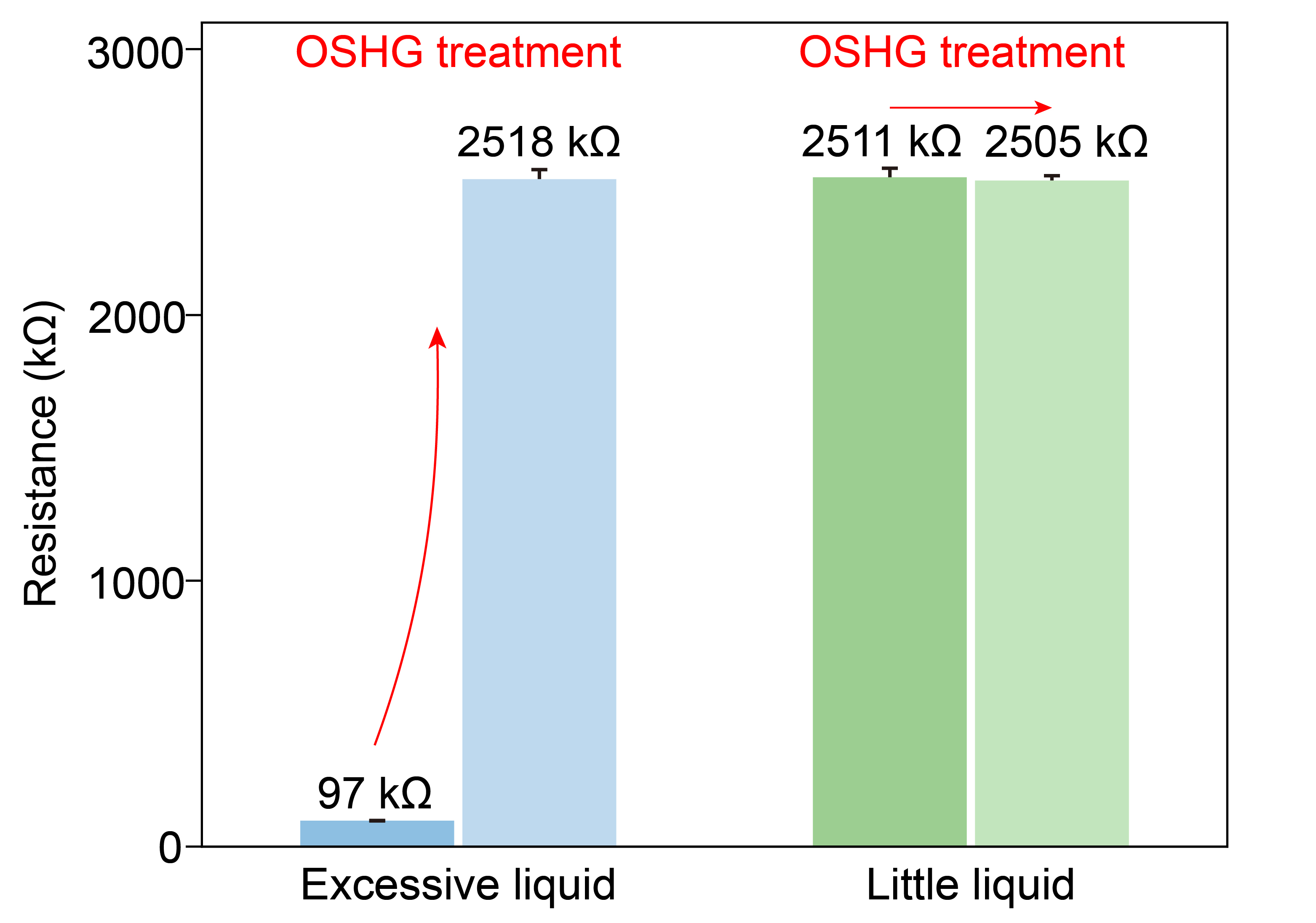


**Fig. S19** The statistical results of resistance in each group.


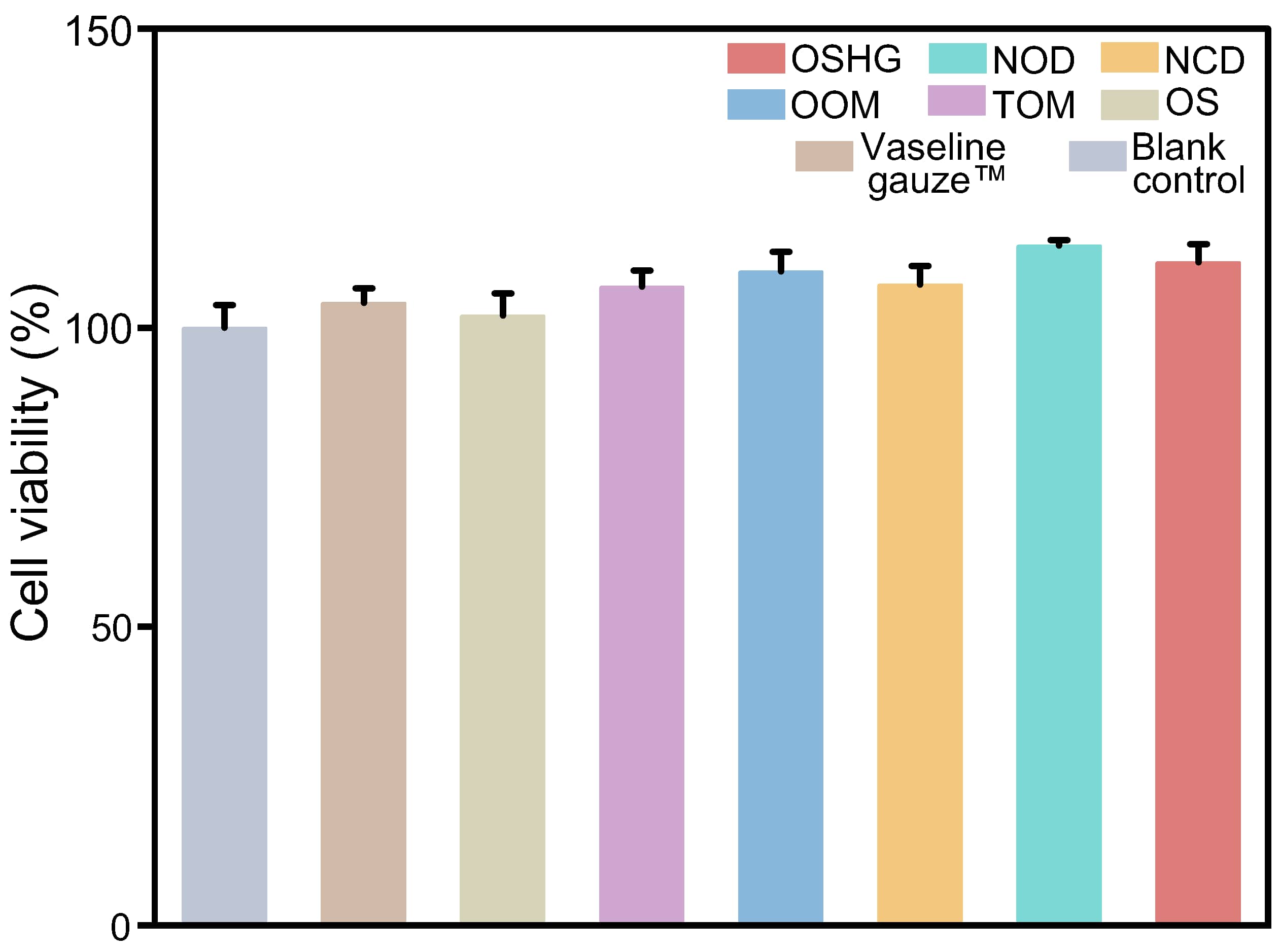


**Fig. S20** Cell viability of the different groups.


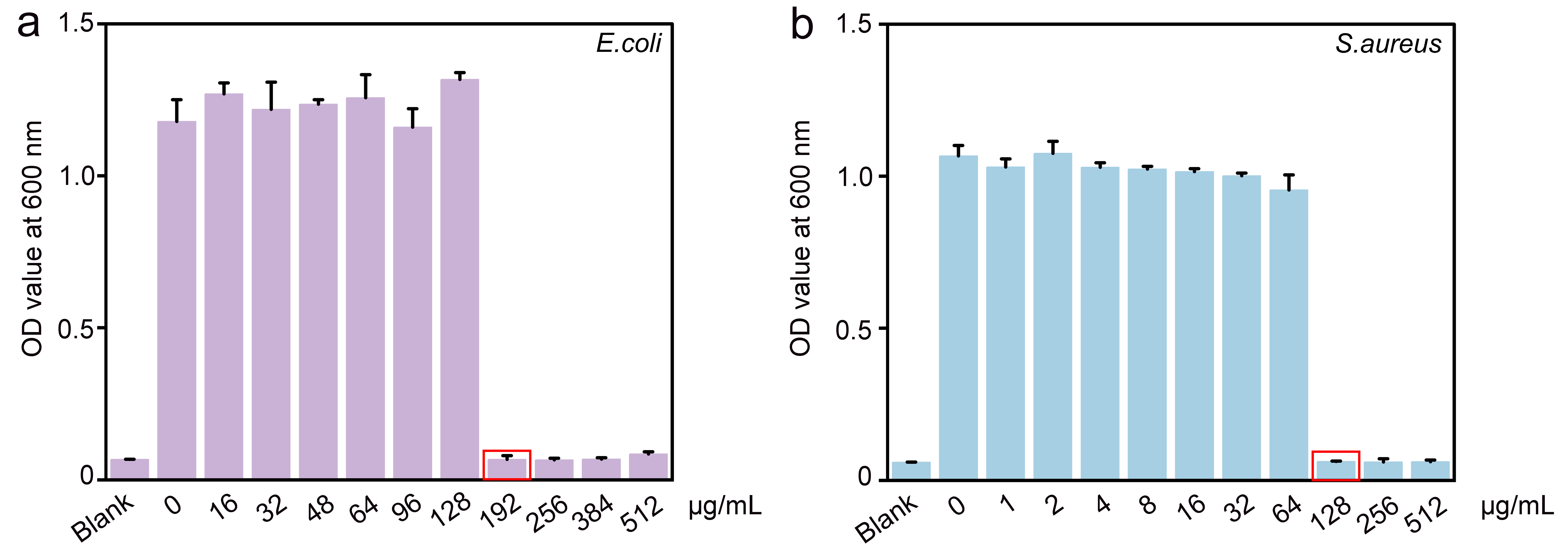


**Fig. S21** The MIC of thymol against *E. coli* and *S. aureus* were determined by OD 600 nm measurement.


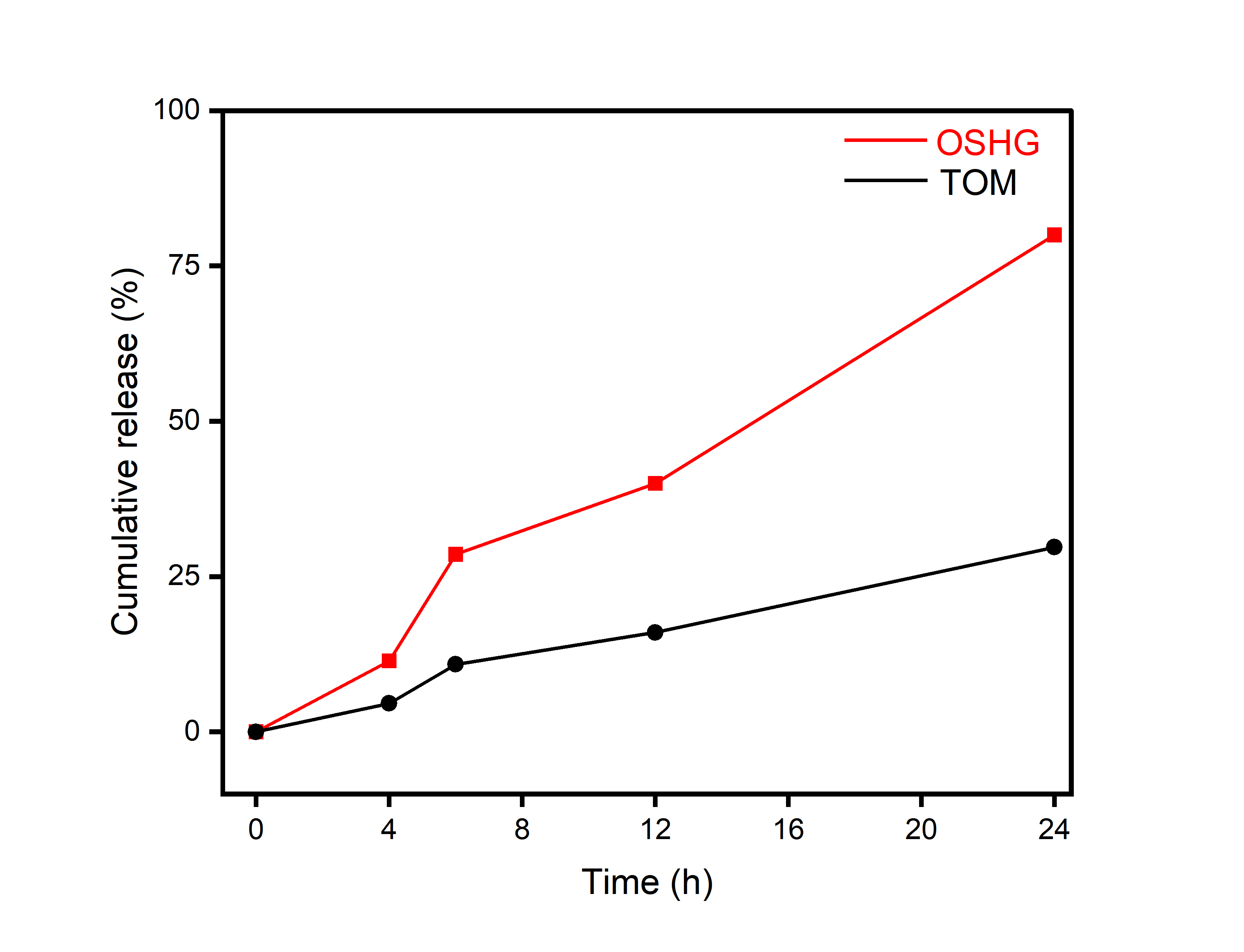


**Fig. S22** Release curves of thymol from the OSHG group (dressing with both medical oil and thymol) and the TOM group (dressing with only thymol but without medical oil).


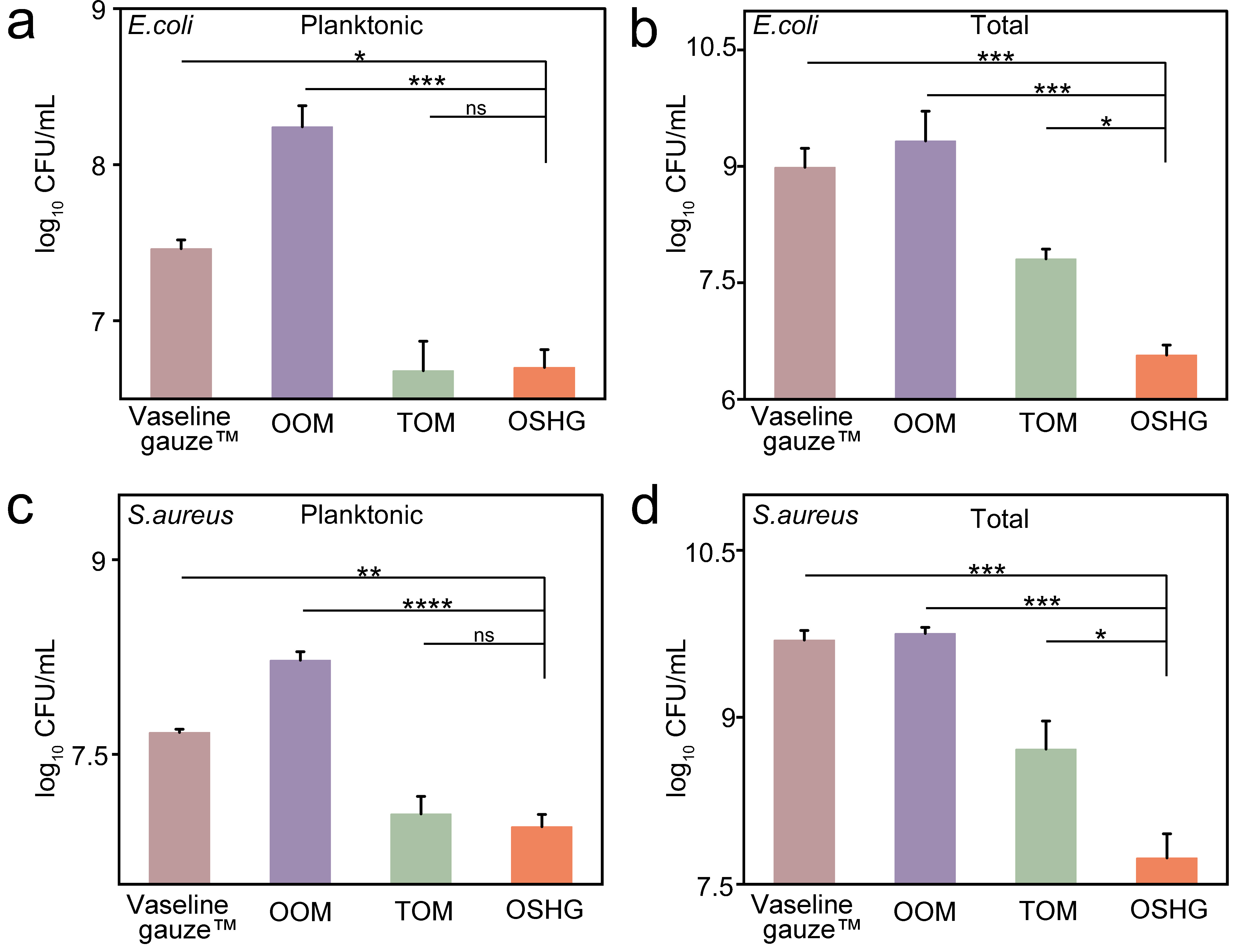


**Fig. S23** Quantification of planktonic and total bacterial count in (a, b) *E. coli* and (c, d) *S. aureus* biofilm systems after different treatments.


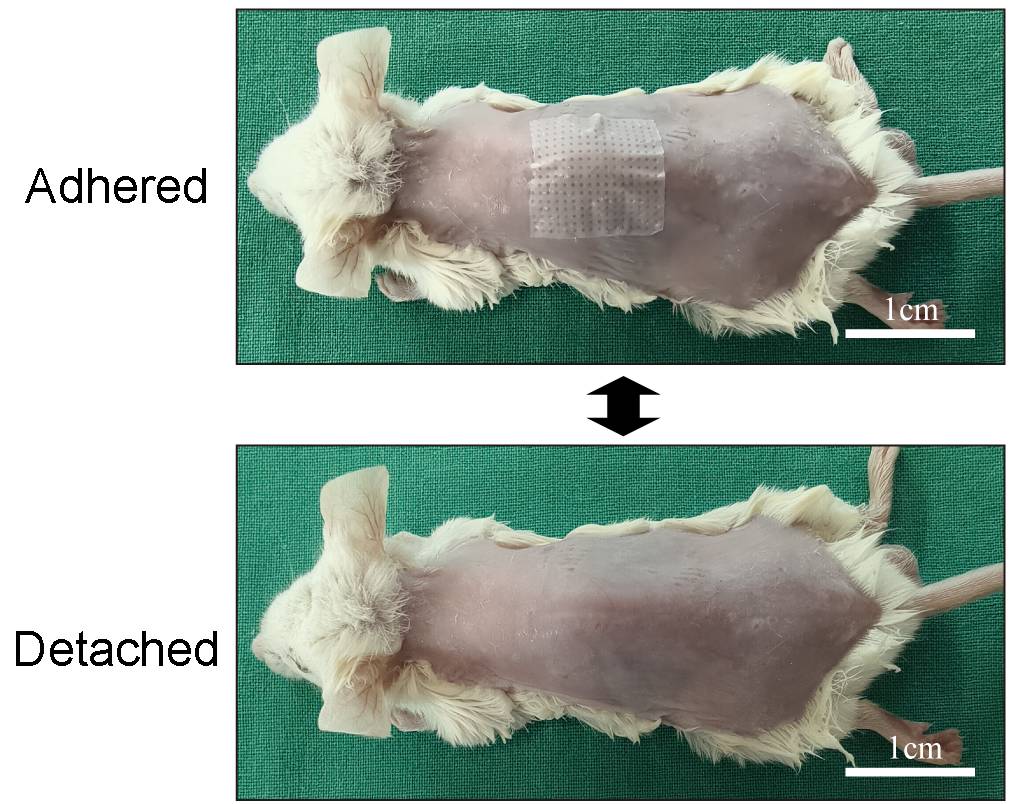


**Fig. S24** Mice skin after being repeatedly peeled 100 times.


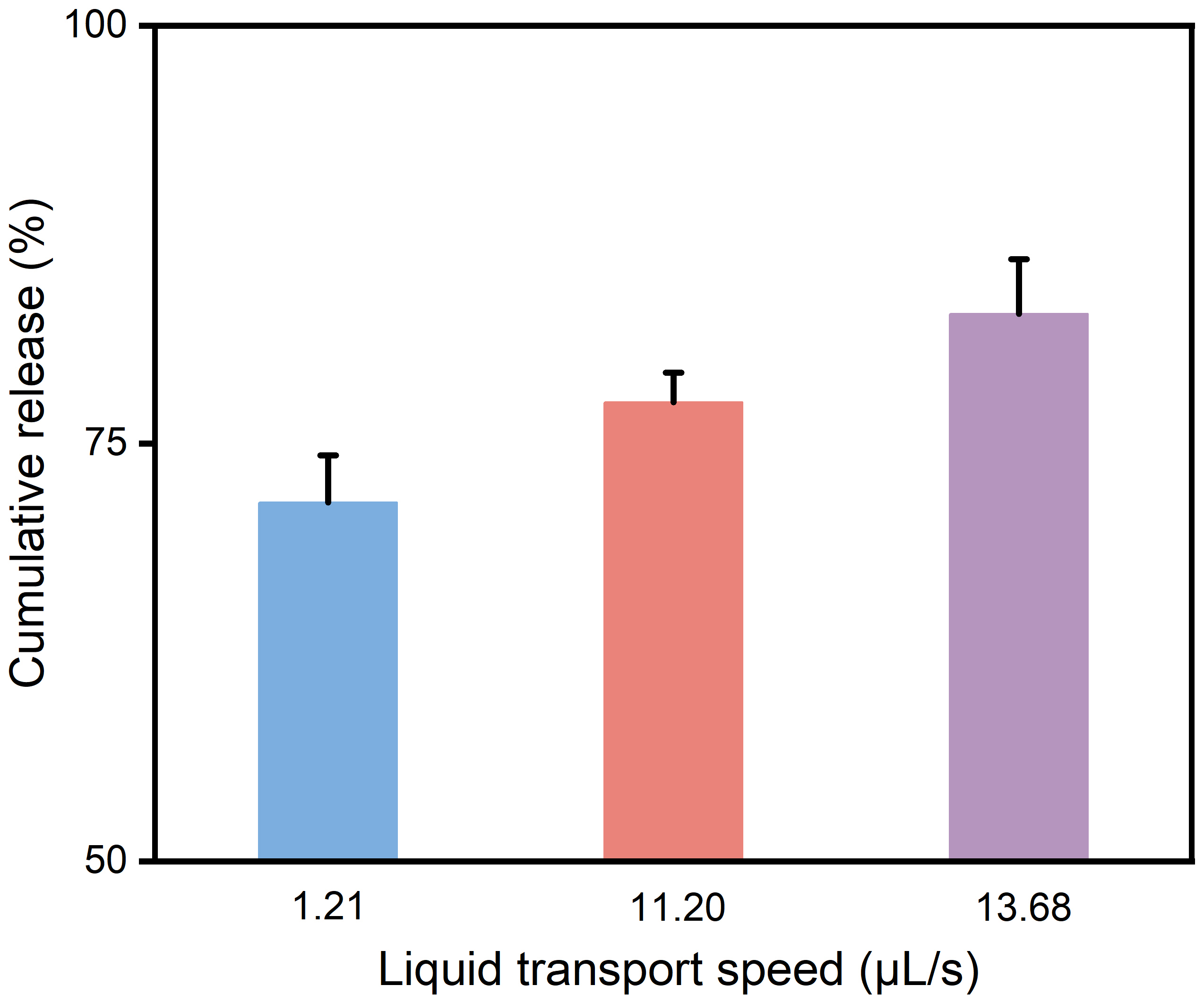


**Fig. S25** Release percentage of the bioinspired biphasic dressings with different liquid transport speeds.


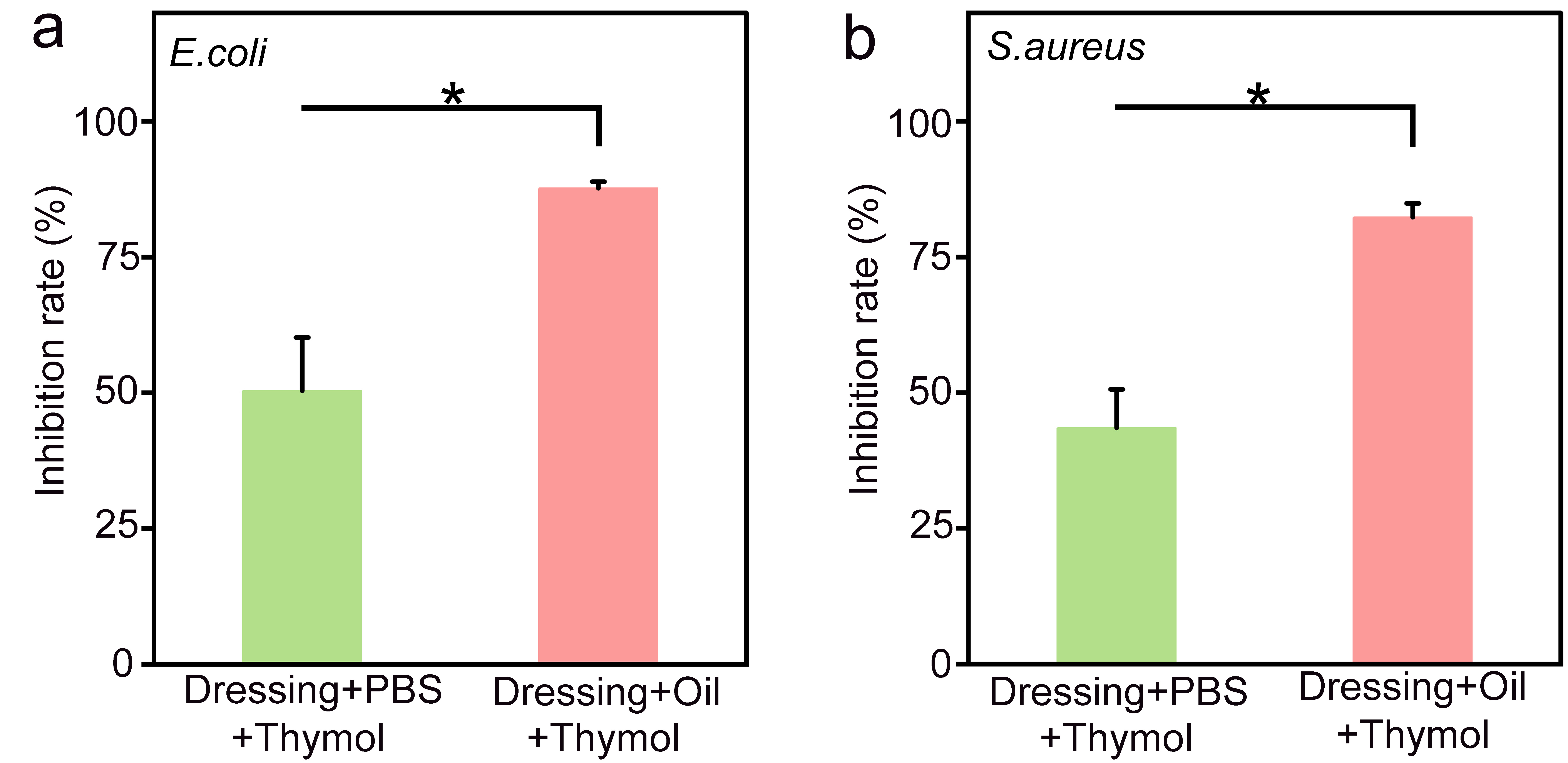


**Fig. S26** Comparison of the antibacterial efficacy of two dressings (the OS membrane loaded with medical oil and thymol, the OS membrane loaded with PBS and thymol) against (a) *E. coli* and (b) *S. aureus*.


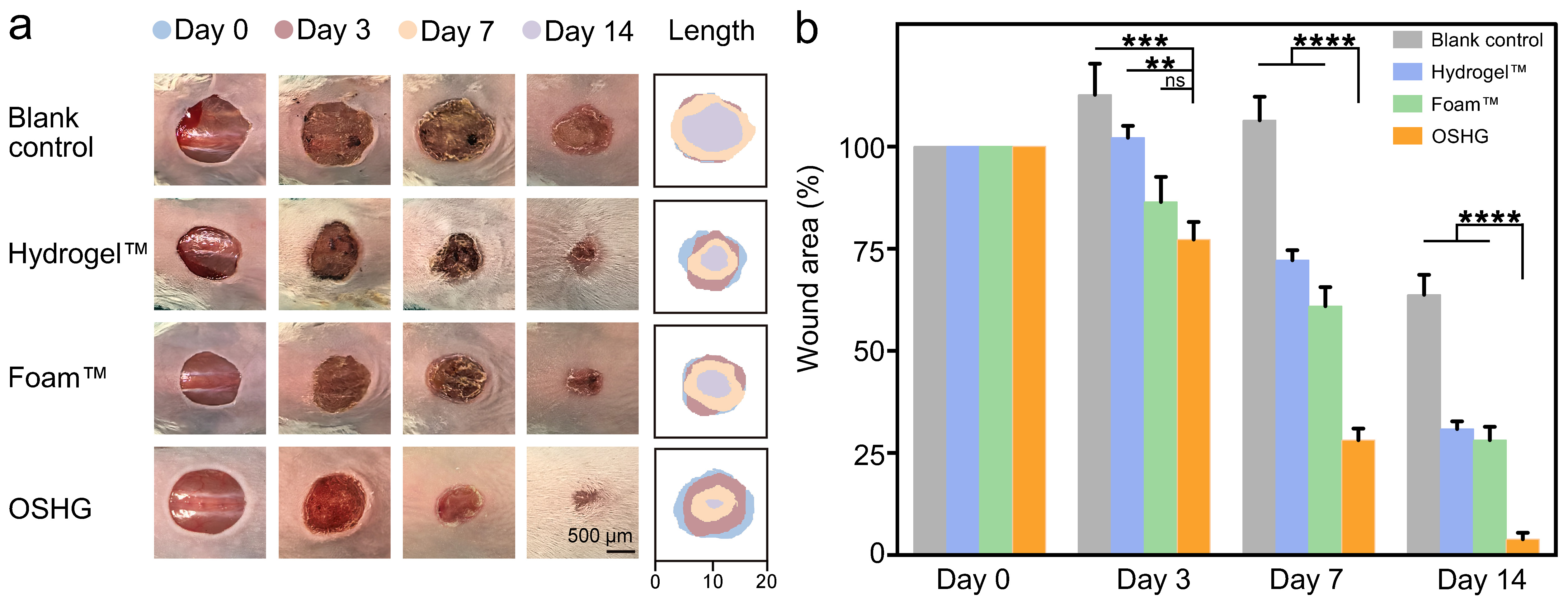


**Fig. S27** Comparison of wound healing process between the bioinspired biphasic OSHG dressing and two commercial exudate management dressings: a hydrogel dressing (releasing moisture to supplement wound hydration) and a foam dressing (absorbing exudate to reduce wound moisture). (a) Representative images of wound healing at different time points. (b) Quantitative analysis of wound closure rates.


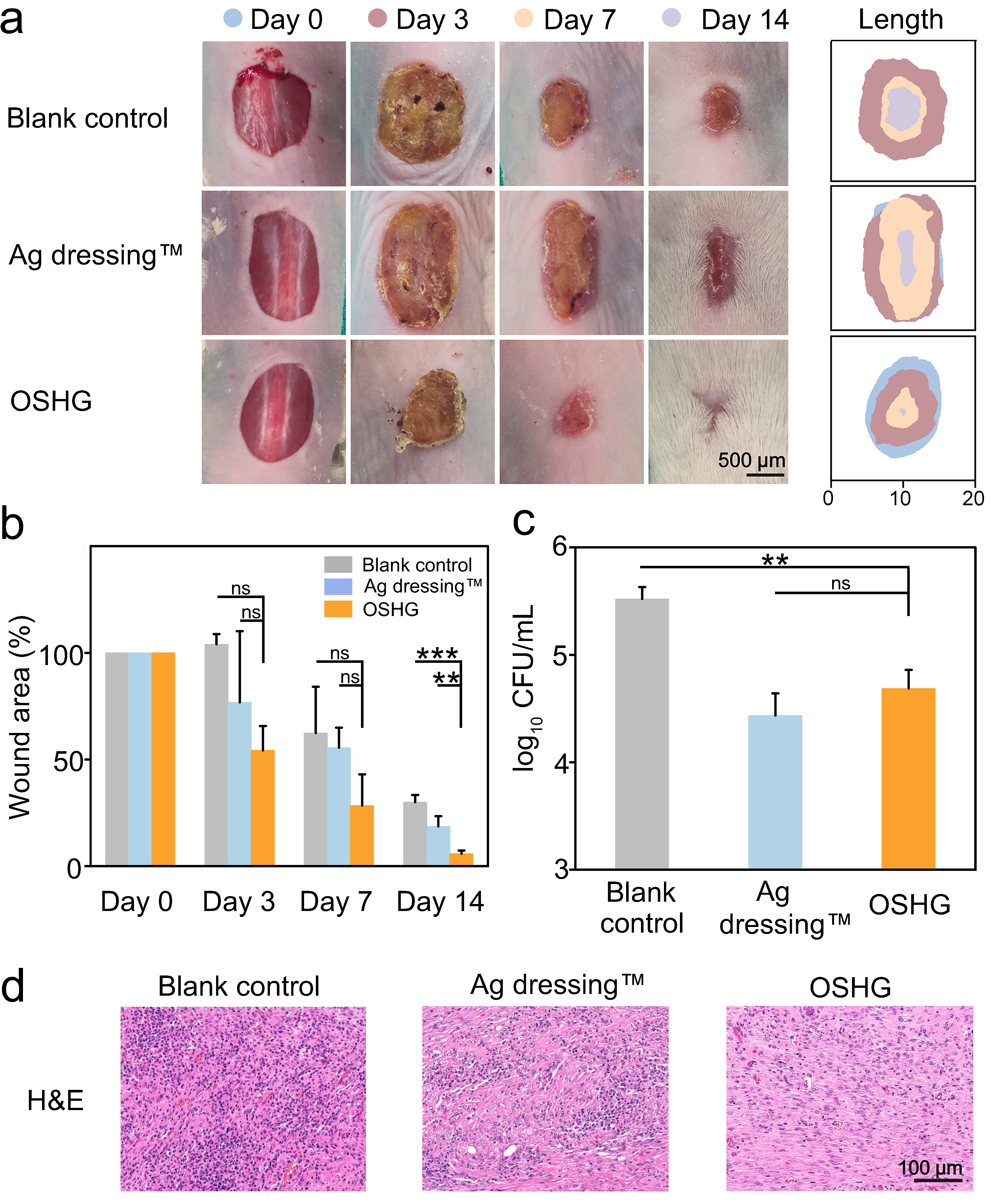


**Fig. S28** Comparison of wound healing process and antibacterial efficacy between the bioinspired biphasic OSHG dressing and commercial silver-containing dressing. (a) Representative images of wound healing at different time points. (b) Quantitative analysis of wound healing rates. (c) Antibacterial activities of the two dressings. (d) H&E staining results.


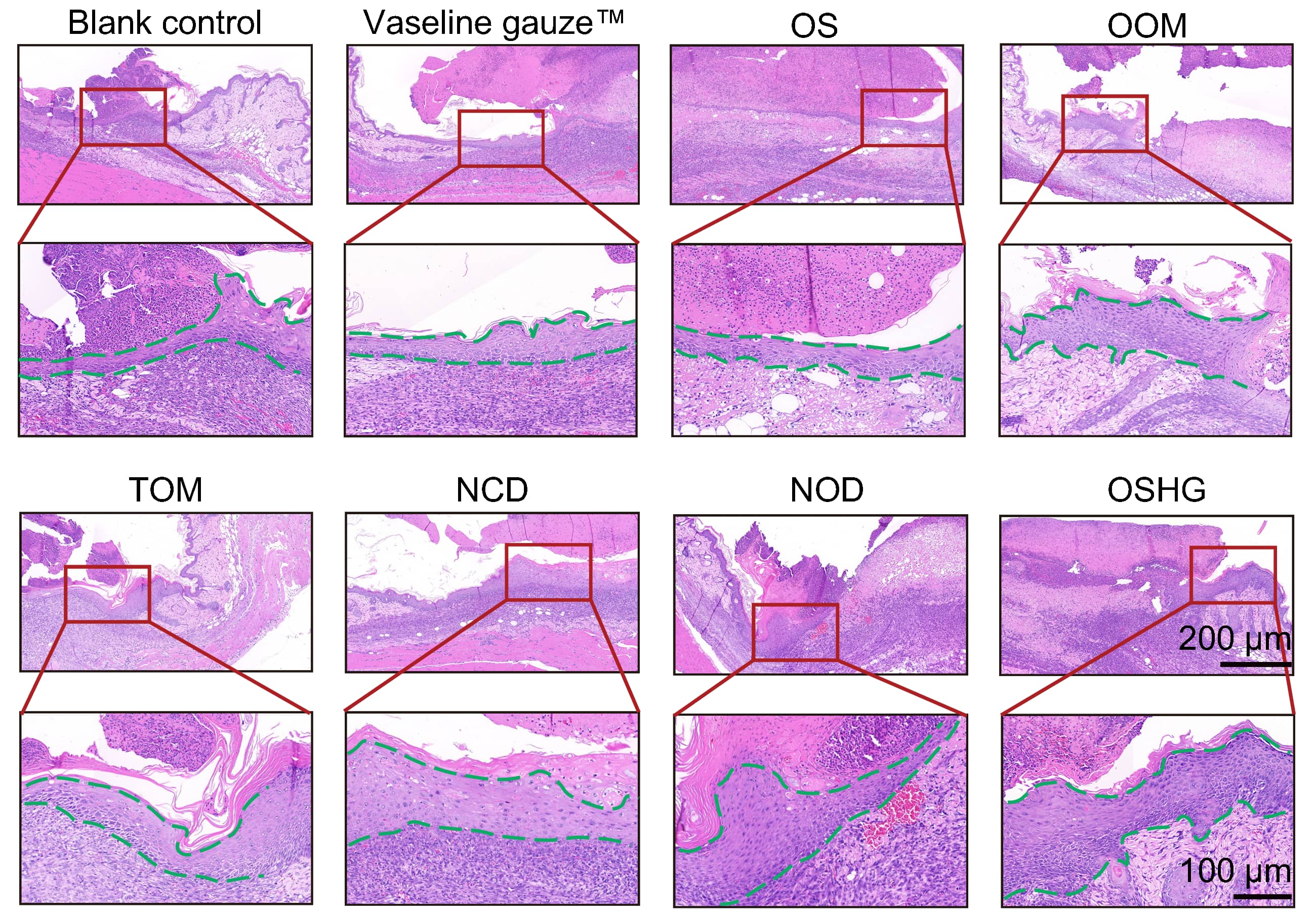


**Fig. S29** H&E staining results of different groups on day 7.


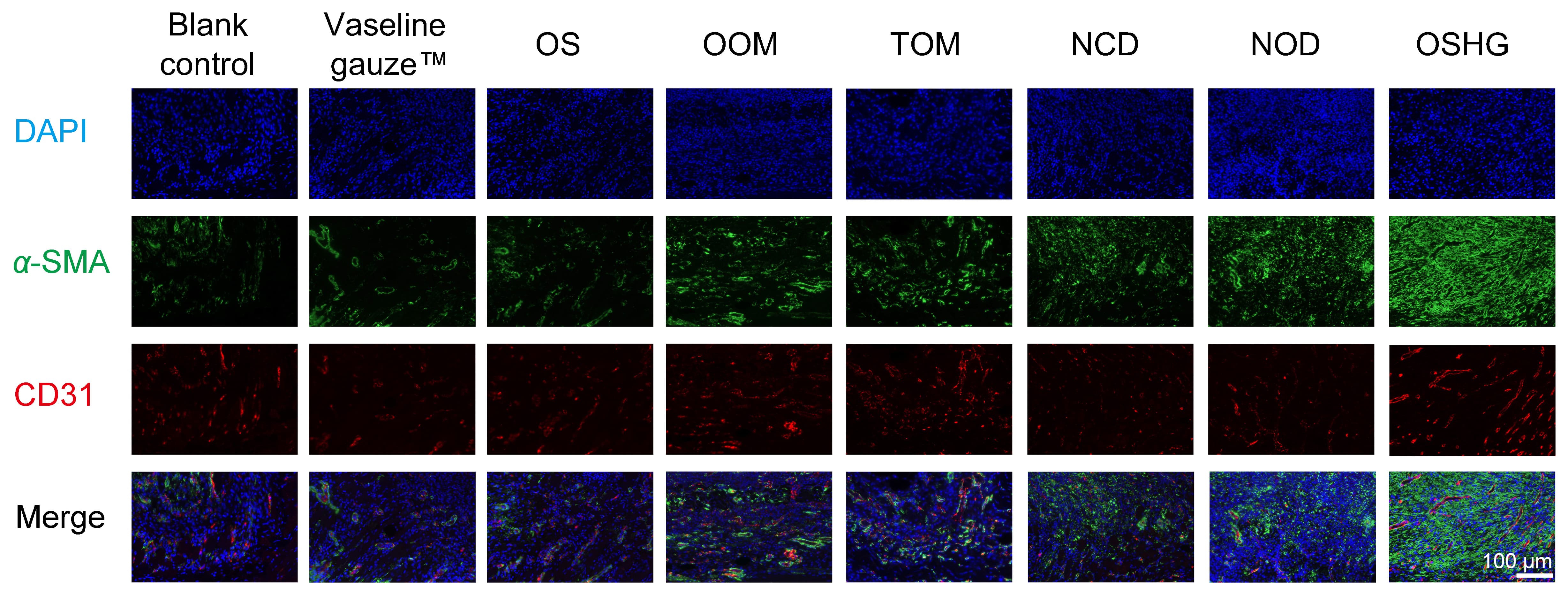


**Fig. S30** Angiogenesis in wound tissues of each group on day 7.


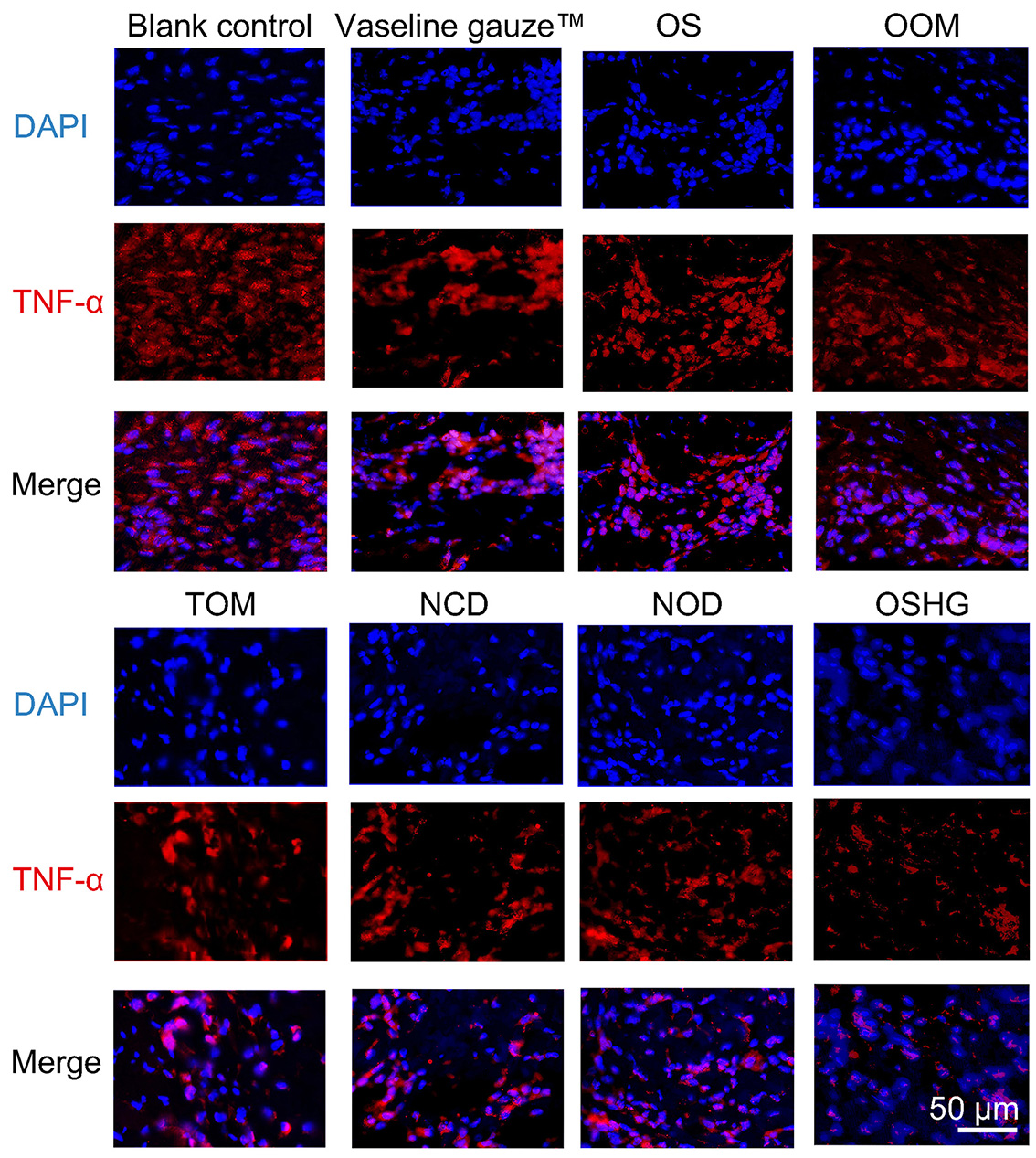


**Fig. S31** The image of the TNF-*α* distribution of different groups on day 14.


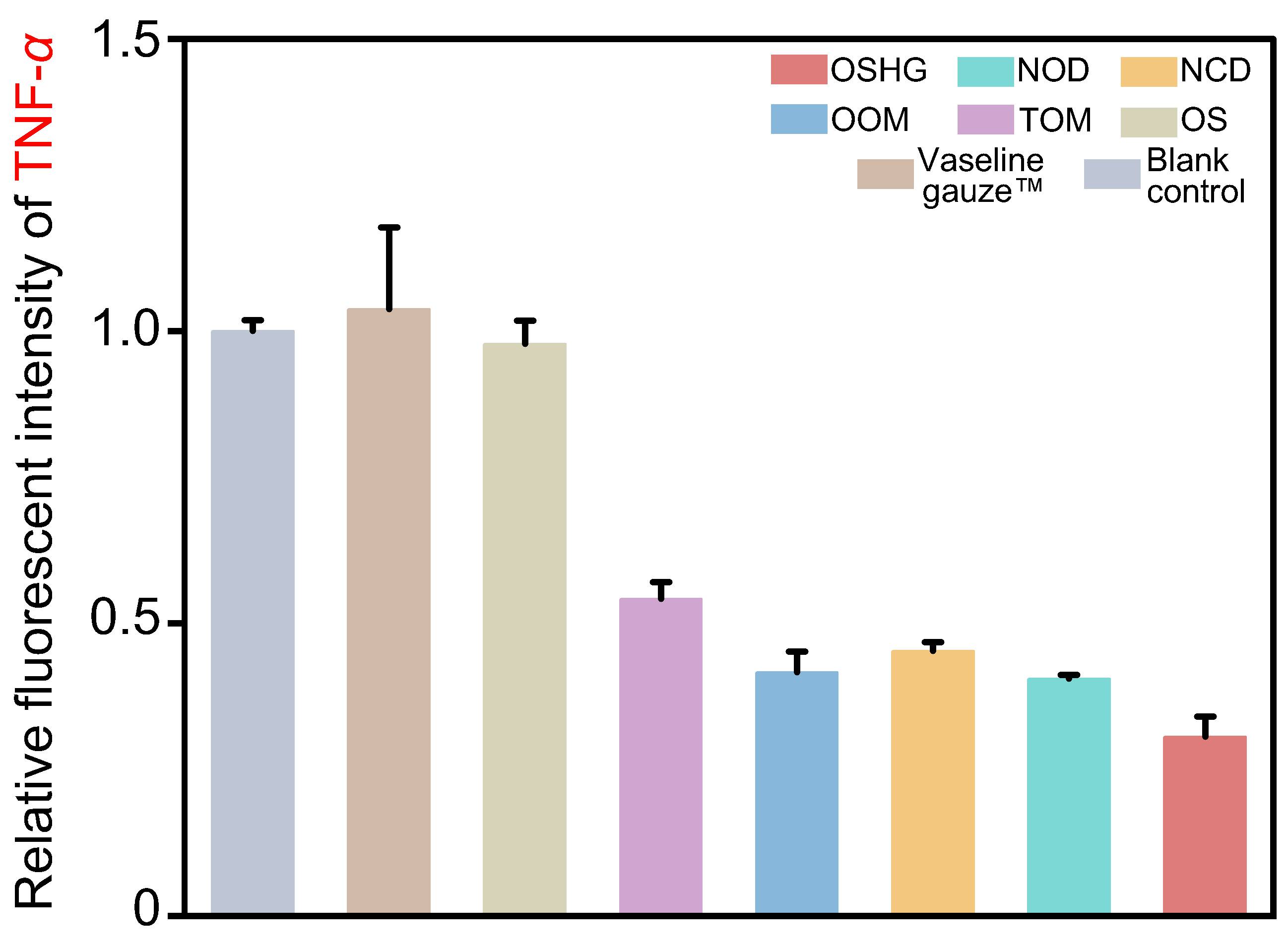


**Fig. S32** Relative fluorescent intensity of TNF-*α* on day 14*.*


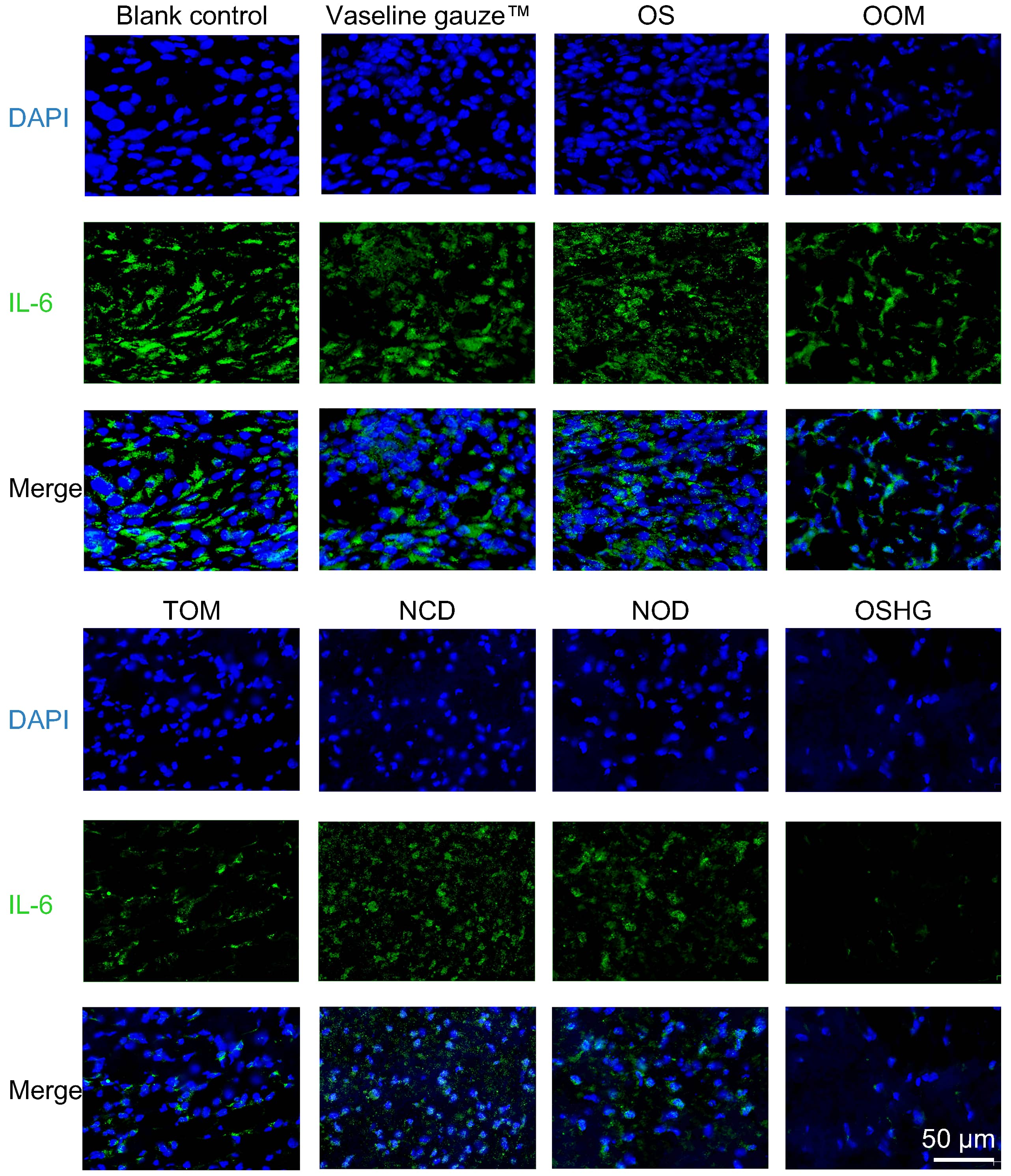


**Fig. S33** The image of the IL-6 distribution of different groups on day 14.


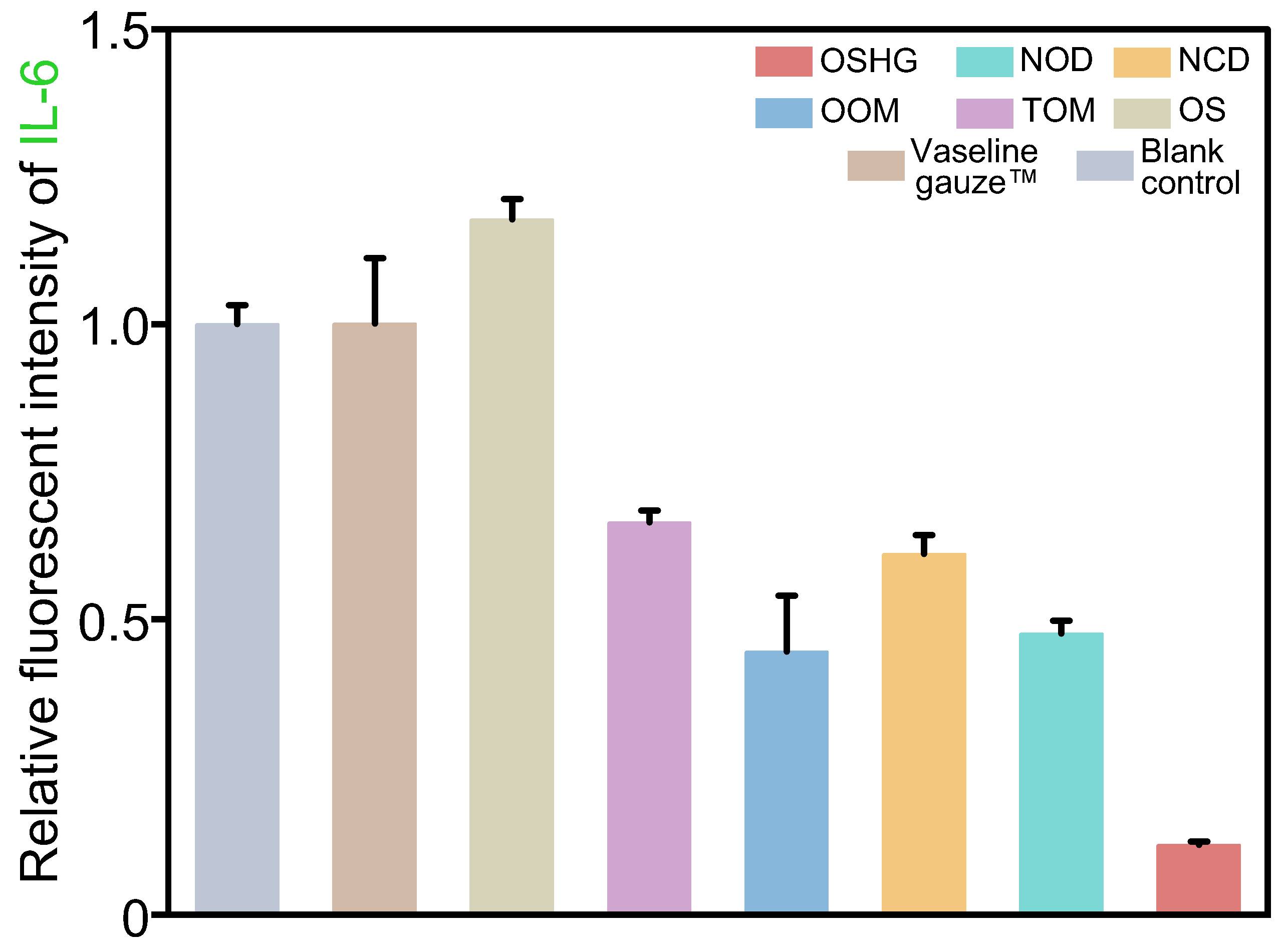


**Fig. S34** Relative fluorescent intensity of IL-6 on day 14*.*


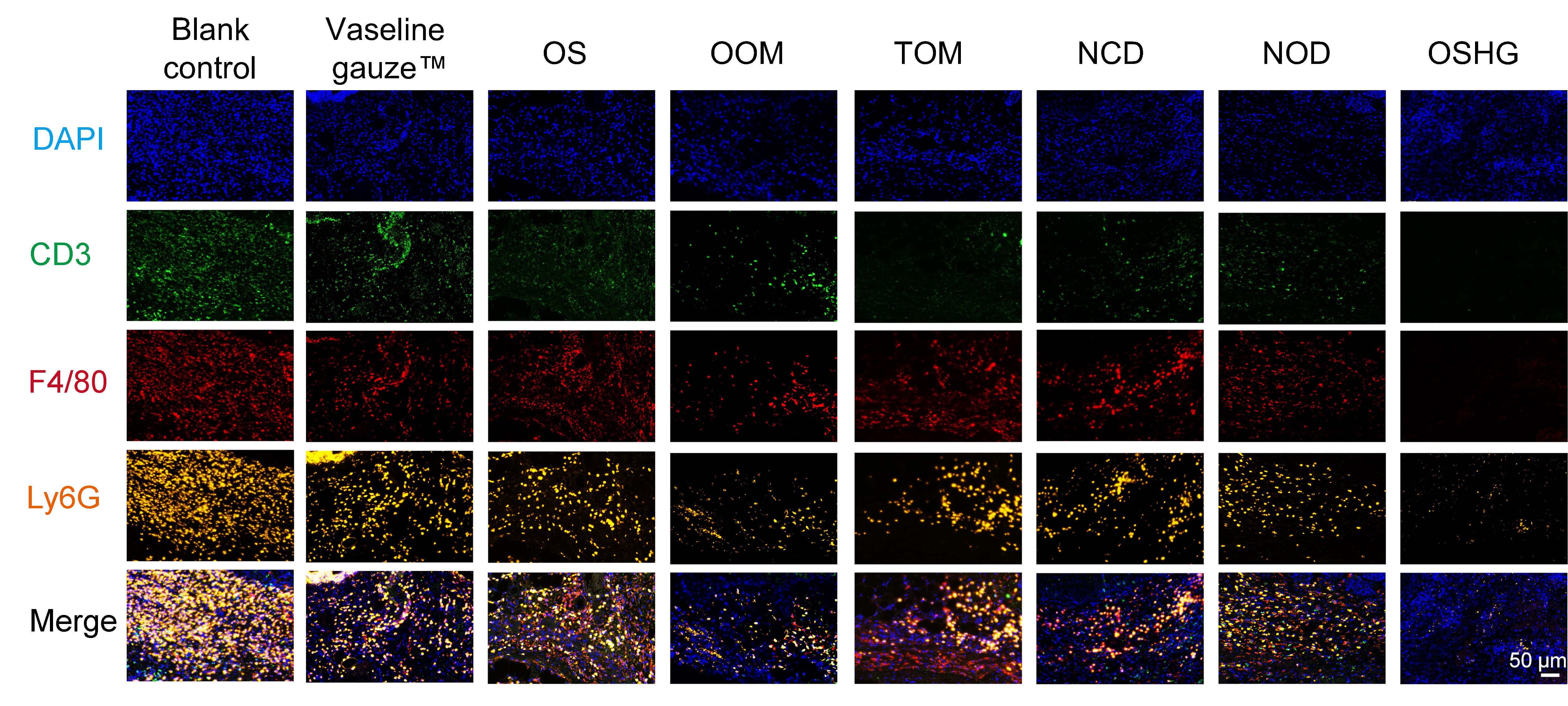


**Fig. S35** Representative immunofluorescence staining images of various immune cells in each group on day 3.


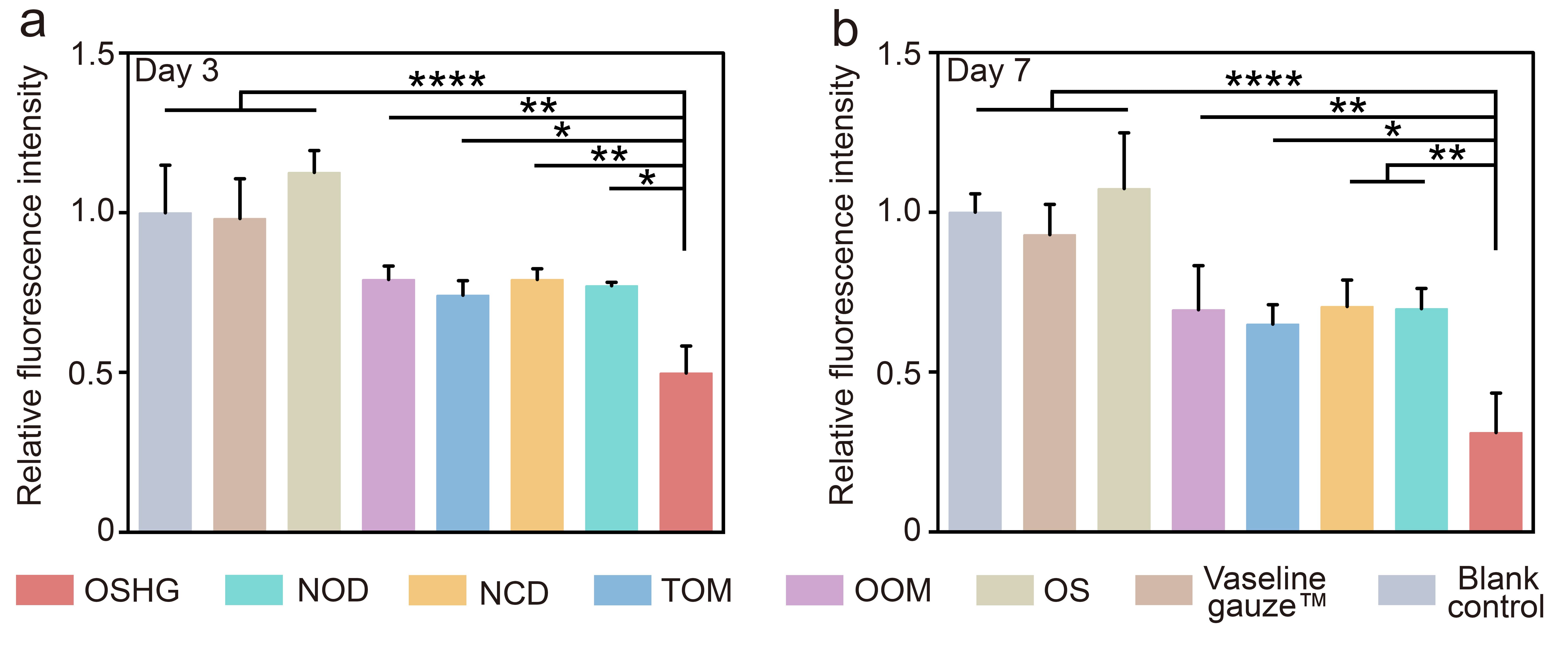


**Fig. S36** The *in vivo* anti-biofilm efficacy in each group.


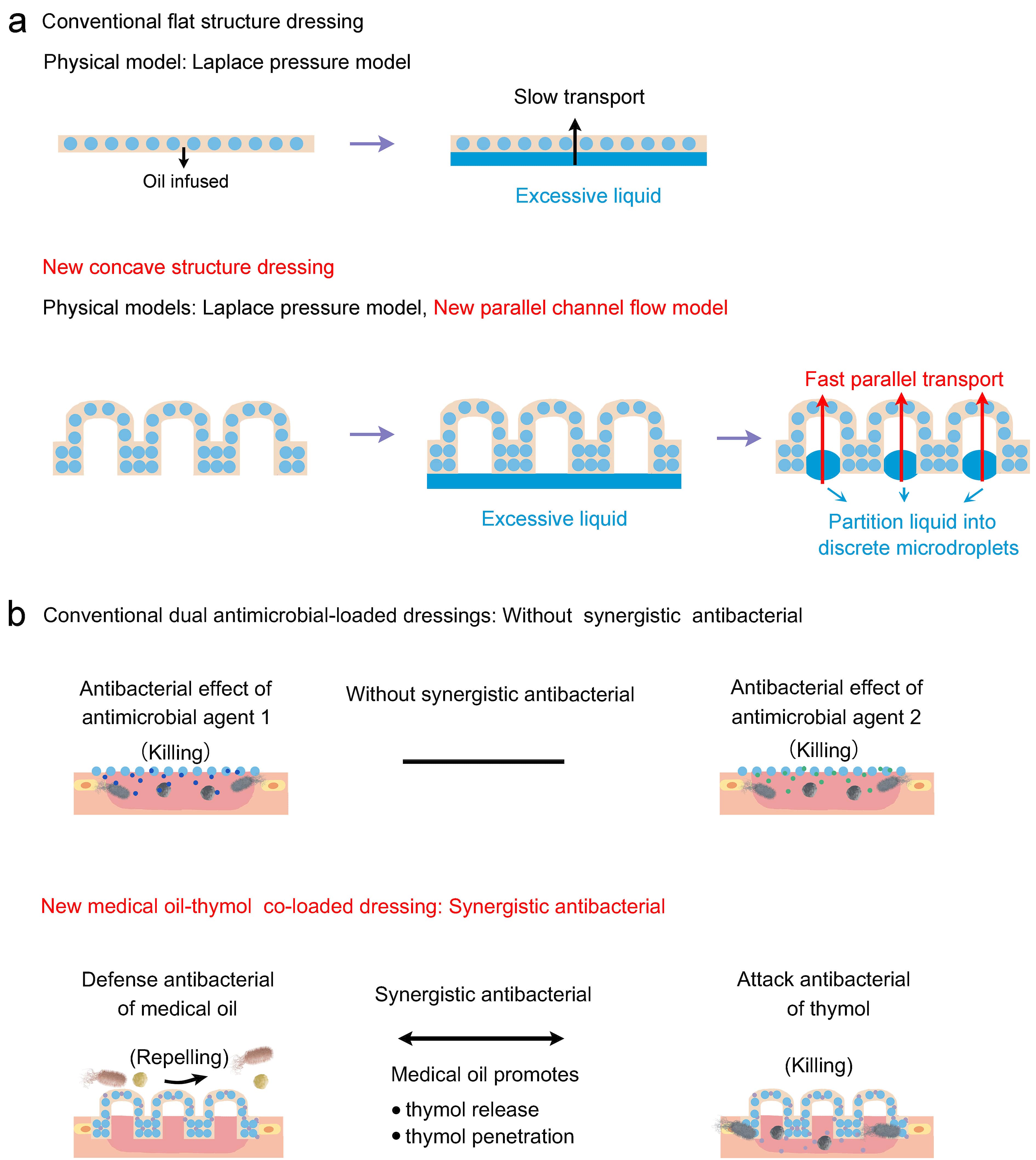


**Fig. S37** (a) Schematic comparison of conventional flat liquid-infused dressings with slow liquid transport versus our concave-mediated parallel channel flow model for fast liquid transport. (b) Schematic comparison of conventional dual antimicrobial-loaded dressings without synergistic antibacterial effect and our medical oil-thymol co-loaded dressing with synergistic antibacterial effect.

1. **Supplementary Tables**

**Table S1.** Comparison of *in vivo* peeling energy of the bioinspired biphasic dressing with reported dressings.

| Dressing types | Peeling energy (J/m^2^) | References |
| --- | --- | --- |
| Bioinspired biphasic dressing | **0.94** | **This work** |
| Bilayered fiber membrane | 43.1 | Surf. Interfaces  2024, 49, 104366. ^[1]^ |
| Foam | 121.67 | Cellulose 2024, 31, 7613. ^[2]^ |
|  | 155 | Burns 2016, 42, 877. ^[3]^ |

**Table S2.** Comparison of functions of the bioinspired biphasic dressing with reported dressings.

| **Dressing types** | **Transport speed (μL/s)** | | **Antibacterial**  **function** | | | **Anti-adhesion**  **function** | **References** |
| --- | --- | --- | --- | --- | --- | --- | --- |
|  |  |  | **Defense** | | **Attact** |  |  |
| **Bioinspired biphasic dressing** | | **11.2** | | **√** | **√** | **√** | **This work** |
| Gated dressing | | 2.5 |  | | √ |  | Adv. Funct. Mater. 2024, 34, 2311997. ^[4]^ |
| Janus dressing | | 10 |  | | √ |  | Adv. Mater. 2019, 31, 1804187. ^[5]^ |
|  |  | 10 |  | |  |  | Small 2022, 18, 2200799. ^[6]^ |
|  |  | 4 | √ | |  |  | Acta Biomater. 2023, 171, 428. ^[7]^ |
|  |  | 3 |  | |  | √ | Chin. Chem. Lett. 2025, 36, 110601. ^[8]^ |

**3.** **Supplementary Movies**

**Movie S1** Transport process of large liquid droplet

**Movie S2** Transport process of small liquid droplet

**Movie S3** The in-situ observation of the transport process of large liquid droplet at 10x speed

**Movie S4** The in-situ observation of the transport process of small liquid droplet at 10x speed

**Movie S5** Liquid droplet transported from the bioinspired biphasic dressing side

**Movie S6** Liquid droplet transported from the hydrophilic side

**4. Supplementary References**

[1] Gu J, Dai X, Liu S, Zhang X, Hu Y, Zhang Y, Zou Z. Preparation of hydrophobic spray-coated spunlace nonwoven fabrics: Possessing the potential for anti-adhesive medical dressings. *Surfaces and Interfaces* **2024**;*49*:104366.

[2] Liu J, Cheng H, Yuan X, Wang L, Gao J. Amphipathic medical composite cotton gauze with unidirectional drainage and anti-adhesion properties for wound healing. *Cellulose* **2024**;*31*:7613.

[3] Asghari S, Logsetty S, Liu S. Imparting commercial antimicrobial dressings with low-adherence to burn wounds. *Burns* **2016**;*42*:877.

[4] Qiu Z, Gao Y, Qi D, Wu M, Mao Z, Wu J. Thermo-responsive trilayered fibrous dressing with liquid gate for dynamical exudate regulation and wound moisture balance. *Adv Funct Mater*. **2024**;*34*:2311997.

[5] Shi L, Liu X, Wang W, Jiang L, Wang S. A self-pumping dressing for draining excessive biofluid around wounds. *Adv Mater.* **2019**;*31*:1804187.

[6] Qian S, Wang J, Liu Z, Mao J, Zhao B, Mao X, Zhang L, Cheng L, Zhang Y, Sun X, Cui W. Secretory fluid-aggregated Janus electrospun short fiber scaffold for wound healing. *Small* **2022**;*18*:2200799.

[7] Chen S, Li A, Wang Y, Zhang Y, Liu X, Ye Z, Gao S, Xu H, Deng L, Dong A, Zhang J. Janus polyurethane sponge as an antibiofouling, antibacterial, and exudate-managing dressing for accelerated wound healing. *Acta Biomater.* **2023**;*171*:428.

[8] Lu B, Wang D, Guo J, Shen Y, Feng Q, Yang J, Han X, Yu H, Li L, Liu J, Luo J, Liu H, Zhang Z, Deng X. High-efficiency exudates drainage of anti-adhesion dressings for chronic wound. *Chin Chem Lett.* **2025**;*36*:110601.
